# Global, regional, and national sex-specific burden and control of the HIV epidemic, 1990–2019, for 204 countries and territories: the Global Burden of Diseases Study 2019

**DOI:** 10.1016/S2352-3018(21)00152-1

**Published:** 2021-09-27

**Authors:** GBD 2019 HIV Collaborators, Deepa Jahagirdar, Magdalene K Walters, Amanda Novotney, Edmond D Brewer, Tahvi D Frank, Austin Carter, Molly H Biehl, Hedayat Abbastabar, E S Abhilash, Eman Abu-Gharbieh, Laith Jamal Abu-Raddad, Victor Adekanmbi, Daniel Adedayo Adeyinka, Qorinah Estiningtyas Sakilah Adnani, Saira Afzal, Soodabeh Aghababaei, Bright Opoku Ahinkorah, Sajjad Ahmad, Keivan Ahmadi, Sepideh Ahmadi, Ehsan Ahmadpour, Muktar Beshir Ahmed, Tarik Ahmed Rashid, Yusra Ahmed Salih, Addis Aklilu, Tayyaba Akram, Chisom Joyqueenet Akunna, Hanadi Al Hamad, Fares Alahdab, Fahad Mashhour Alanezi, Ekaterina A Aleksandrova, Kefyalew Addis Alene, Liaqat Ali, Vahid Alipour, Sami Almustanyir, Nelson Alvis-Guzman, Edward Kwabena Ameyaw, Hubert Amu, Catalina Liliana Andrei, Tudorel Andrei, Davood Anvari, Jalal Arabloo, Olatunde Aremu, Judie Arulappan, Desta Debalkie Atnafu, Beatriz Paulina Ayala Quintanilla, Muluken Altaye Ayza, Samad Azari, Darshan B B, Maciej Banach, Till Winfried Bärnighausen, Fabio Barra, Amadou Barrow, Sanjay Basu, Shahrzad Bazargan-Hejazi, Habtamu Gebrehana Belay, Tezera Moshago Berheto, Woldesellassie Mequanint Bezabhe, Yihienew Mequanint Bezabih, Akshaya Srikanth Bhagavathula, Nikha Bhardwaj, Pankaj Bhardwaj, Krittika Bhattacharyya, Sadia Bibi, Ali Bijani, Catherine Bisignano, Obasanjo Afolabi Bolarinwa, Archith Boloor, Azizbek A Boltaev, Nikolay Ivanovich Briko, Danilo Buonsenso, Katrin Burkart, Zahid A Butt, Chao Cao, Jaykaran Charan, Souranshu Chatterjee, Soosanna Kumary Chattu, Vijay Kumar Chattu, Sonali Gajanan Choudhari, Dinh-Toi Chu, Rosa A S Couto, Richard G Cowden, Berihun Assefa Dachew, Omid Dadras, Amare Belachew Dagnew, Saad M A Dahlawi, Xiaochen Dai, Lalit Dandona, Rakhi Dandona, José das Neves, Louisa Degenhardt, Feleke Mekonnen Demeke, Abebaw Alemayehu Desta, Keshab Deuba, Deepak Dhamnetiya, Govinda Prasad Dhungana, Mostafa Dianatinasab, Daniel Diaz, Shirin Djalalinia, Linh Phuong Doan, Fariba Dorostkar, Hisham Atan Edinur, Andem Effiong, Sahar Eftekharzadeh, Maysaa El Sayed Zaki, Rajesh Elayedath, Muhammed Elhadi, Shaimaa I El-Jaafary, Ziad El-Khatib, Aisha Elsharkawy, Aklilu Endalamaw, Aman Yesuf Endries, Sharareh Eskandarieh, Ifeanyi Jude Ezeonwumelu, Sayeh Ezzikouri, Mohammad Farahmand, Emerito Jose A Faraon, Abidemi Omolara Fasanmi, Simone Ferrero, Lorenzo Ferro Desideri, Irina Filip, Florian Fischer, Morenike Oluwatoyin Folayan, Masoud Foroutan, Takeshi Fukumoto, Mohamed M Gad, Muktar A Gadanya, Abhay Motiramji Gaidhane, Tushar Garg, Reta Tsegaye Gayesa, Eyob Alemayehu Gebreyohannes, Hailay Abrha Gesesew, Abera Getachew Obsa, Keyghobad Ghadiri, Ahmad Ghashghaee, Syed Amir Gilani, Themba G Ginindza, Ionela-Roxana Glavan, Ekaterina Vladimirovna Glushkova, Mahaveer Golechha, Harish Chander Gugnani, Bhawna Gupta, Sapna Gupta, Veer Bala Gupta, Vivek Kumar Gupta, Samer Hamidi, Senad Handanagic, Shafiul Haque, Harapan Harapan, Arief Hargono, Ahmed I Hasaballah, Abdiwahab Hashi, Shoaib Hassan, Soheil Hassanipour, Khezar Hayat, Ileana Heredia-Pi, Kamal Hezam, Ramesh Holla, Praveen Hoogar, Mohammad Enamul Hoque, Mostafa Hosseini, Mehdi Hosseinzadeh, Mohamed Hsairi, Rabia Hussain, Segun Emmanuel Ibitoye, Bulat Idrisov, Kevin S Ikuta, Olayinka Stephen Ilesanmi, Irena M Ilic, Milena D Ilic, Seyed Sina Naghibi Irvani, M Mofizul Islam, Nahlah Elkudssiah Ismail, Ramaiah Itumalla, Ihoghosa Osamuyi Iyamu, Roxana Jabbarinejad, Vardhmaan Jain, Ranil Jayawardena, Ravi Prakash Jha, Nitin Joseph, Ali Kabir, Zubair Kabir, Rohollah Kalhor, Feroze Kaliyadan, Ashwin Kamath, Tanuj Kanchan, Himal Kandel, Getinet Kassahun, Patrick DMC Katoto, Gbenga A Kayode, Ermiyas Mulu Kebede, Hafte Kahsay Kebede, Himanshu Khajuria, Nauman Khalid, Ejaz Ahmad Khan, Gulfaraz Khan, Khaled Khatab, Min Seo Kim, Yun Jin Kim, Adnan Kisa, Sezer Kisa, Sonali Kochhar, Vladimir Andreevich Korshunov, Parvaiz A Koul, Sindhura Lakshmi Koulmane Laxminarayana, Ai Koyanagi, Kewal Krishan, Barthelemy Kuate Defo, G Anil Kumar, Manasi Kumar, Nithin Kumar, Alexander Kwarteng, Dharmesh Kumar Lal, Iván Landires, Savita Lasrado, Zohra S Lassi, Jeffrey V Lazarus, Jane Jean-Hee Lee, Yeong Yeh Lee, Kate E LeGrand, Christine Lin, Xuefeng Liu, Emilie R Maddison, Hassan Magdy Abd El Razek, Phetole Walter Mahasha, Azeem Majeed, Alaa Makki, Ahmad Azam Malik, Wondimu Ayele Manamo, Mohammad Ali Mansournia, Francisco Rogerlândio Martins-Melo, Seyedeh Zahra Masoumi, Ziad A Memish, Ritesh G Menezes, Endalkachew Worku Mengesha, Hayimro Edemealem Merie, Amanual Getnet Mersha, Tomislav Mestrovic, Peter Meylakhs, Nour Mheidly, Ted R Miller, Andreea Mirica, Babak Moazen, Yousef Mohammad, Mokhtar Mohammadi, Arif Mohammed, Salahuddin Mohammed, Shafiu Mohammed, Modhurima Moitra, Ali H Mokdad, Mariam Molokhia, Mohammad Ali Moni, Ghobad Moradi, Yousef Moradi, Christine Mpundu-Kaambwa, Sumaira Mubarik, Sandra B Munro, Lillian Mwanri, Jean B Nachega, Ahamarshan Jayaraman Nagarajan, Aparna Ichalangod Narayana, Muhammad Naveed, Biswa Prakash Nayak, Sabina O Nduaguba, Sandhya Neupane Kandel, Georges Nguefack-Tsague, Trang Huyen Nguyen, Molly R Nixon, Chukwudi A Nnaji, Jean Jacques Noubiap, Virginia Nuñez-Samudio, Thomas Elliot Nyirenda, Onome Bright Oghenetega, Andrew T Olagunju, Babayemi Oluwaseun Olakunde, Oluwatomi Funbi Owopetu, Mahesh P A, Jagadish Rao Padubidri, Smita Pakhale, Tarang Parekh, Fatemeh Pashazadeh Kan, Shrikant Pawar, Veincent Christian Filipino Pepito, Emmanuel K Peprah, Marina Pinheiro, Khem Narayan Pokhrel, Roman V Polibin, Richard Charles G Pollok, Maarten J Postma, Zahiruddin Quazi Syed, Amir Radfar, Raghu Anekal Radhakrishnan, Fakher Rahim, Vafa Rahimi-Movaghar, Shadi Rahimzadeh, Mosiur Rahman, Amir Masoud Rahmani, Pradhum Ram, Chhabi Lal Ranabhat, Priyanga Ranasinghe, Chythra R Rao, Sowmya J Rao, Priya Rathi, David Laith Rawaf, Salman Rawaf, Lemma Demissie Regassa, Inayat ur Rehman, Andre M N Renzaho, Nima Rezaei, Omid Rezahosseini, Mohammad sadegh Rezai, Aziz Rezapour, Rezaul Karim Ripon, Voilet Rodrigues, Denis O Roshchin, Godfrey M Rwegerera, Umar Saeed, Sahar Saeedi Moghaddam, Rajesh Sagar, KM Saif-Ur-Rahman, Marwa Rashad Salem, Mehrnoosh Samaei, Abdallah M Samy, Milena M Santric-Milicevic, Satish Saroshe, Brijesh Sathian, Maheswar Satpathy, Monika Sawhney, Aletta Elisabeth Schutte, Allen Seylani, Masood Ali Shaikh, Mohammed Feyisso Shaka, Hina Shamshad, Morteza Shamsizadeh, Mohammed Shannawaz, Adithi Shetty, Jae Il Shin, K M Shivakumar, Jasvinder A Singh, Valentin Yurievich Skryabin, Anna Aleksandrovna Skryabina, Ranjani Somayaji, Sergey Soshnikov, Emma Elizabeth Spurlock, Dan J Stein, Mu'awiyyah Babale Sufiyan, Hooman Tadbiri, Birkneh Tilahun Tadesse, Eyayou Girma Tadesse, Animut Tagele Tamiru, Elvis Enowbeyang Tarkang, Nuno Taveira, Yohannes Tekalegn, Fisaha Haile Tesfay, Gizachew Assefa Tessema, Rekha Thapar, Marcos Roberto Tovani-Palone, Eugenio Traini, Bach Xuan Tran, Alexander C Tsai, Biruk Shalmeno Tusa, Saif Ullah, Chukwuma David Umeokonkwo, Bhaskaran Unnikrishnan, Sahel Valadan Tahbaz, Jorge Hugo Villafañe, Sergey Konstantinovitch Vladimirov, Bay Vo, Avina Vongpradith, Giang Thu Vu, Yasir Waheed, Richard G Wamai, Guan Wang, Yanzhong Wang, Paul Ward, Ronny Westerman, Andrea Sylvia Winkler, Lalit Yadav, Seyed Hossein Yahyazadeh Jabbari, Taklo Simeneh Yazie, Siyan Yi, Vahit Yigit, Birhanu Wubale Yirdaw, Naohiro Yonemoto, Chuanhua Yu, Ismaeel Yunusa, Mikhail Sergeevich Zastrozhin, Anasthasia Zastrozhina, Zhi-Jiang Zhang, Alimuddin Zumla, Joshua A Salomon, Jeffrey W Eaton, Mohsen Naghavi, Laura Dwyer-Lindgren, Haidong Wang, Stephen S Lim, Simon I Hay, Christopher J L Murray, Hmwe Hmwe Kyu

## Abstract

**Background:**

The sustainable development goals (SDGs) aim to end HIV/AIDS as a public health threat by 2030. Understanding the current state of the HIV epidemic and its change over time is essential to this effort. This study assesses the current sex-specific HIV burden in 204 countries and territories and measures progress in the control of the epidemic.

**Methods:**

To estimate age-specific and sex-specific trends in 48 of 204 countries, we extended the Estimation and Projection Package Age-Sex Model to also implement the spectrum paediatric model. We used this model in cases where age and sex specific HIV-seroprevalence surveys and antenatal care-clinic sentinel surveillance data were available. For the remaining 156 of 204 locations, we developed a cohort-incidence bias adjustment to derive incidence as a function of cause-of-death data from vital registration systems. The incidence was input to a custom Spectrum model. To assess progress, we measured the percentage change in incident cases and deaths between 2010 and 2019 (threshold >75% decline), the ratio of incident cases to number of people living with HIV (incidence-to-prevalence ratio threshold <0·03), and the ratio of incident cases to deaths (incidence-to-mortality ratio threshold <1·0).

**Findings:**

In 2019, there were 36·8 million (95% uncertainty interval [UI] 35·1–38·9) people living with HIV worldwide. There were 0·84 males (95% UI 0·78–0·91) per female living with HIV in 2019, 0·99 male infections (0·91–1·10) for every female infection, and 1·02 male deaths (0·95–1·10) per female death. Global progress in incident cases and deaths between 2010 and 2019 was driven by sub-Saharan Africa (with a 28·52% decrease in incident cases, 95% UI 19·58–35·43, and a 39·66% decrease in deaths, 36·49–42·36). Elsewhere, the incidence remained stable or increased, whereas deaths generally decreased. In 2019, the global incidence-to-prevalence ratio was 0·05 (95% UI 0·05–0·06) and the global incidence-to-mortality ratio was 1·94 (1·76–2·12). No regions met suggested thresholds for progress.

**Interpretation:**

Sub-Saharan Africa had both the highest HIV burden and the greatest progress between 1990 and 2019. The number of incident cases and deaths in males and females approached parity in 2019, although there remained more females with HIV than males with HIV. Globally, the HIV epidemic is far from the UNAIDS benchmarks on progress metrics.

**Funding:**

The Bill & Melinda Gates Foundation, the National Institute of Mental Health of the US National Institutes of Health (NIH), and the National Institute on Aging of the NIH.

## Introduction

Millennium development goal 6 aimed to halt and reverse the spread of HIV/AIDS by 2015 and to achieve universal access to treatment by 2010.^1^ Although the world fell short of these goals, over the past two decades, HIV deaths and incident cases declined substantially.^2^ The sustainable development goals (SDGs) subsequently set a goal of ensuring healthy lives, which includes a promise by member states to end the AIDS epidemic as a public health threat by 2030.^3^ Assessing progress in the control of the HIV epidemic towards this aim, while remaining cognisant of sex-specific trends, is essential. Establishing this baseline before the indirect effects of the COVID-19 pandemic have been fully realised is also crucial, because these effects might bear on the recalibration of future targets.^4^, ^5^

Measurable targets relating to incidence and deaths have been proposed to assess progress in HIV, but they have not been synthesised to provide a comprehensive assessment of HIV burden. The original 90-90-90 targets were proposed in 2014,^6^ and set out that by 2020, 90% of people living with HIV will know their HIV status, 90% of those who are aware of their HIV-positive status will have initiated antiretroviral therapy (ART), and 90% of those on treatment will be virally suppressed. However, improvements in these metrics might not necessarily reflect progress and data might be misreported;^7^ underreporting presents a problem for any modelling effort that relies on treatment coverage to inform disease-burden estimates. Several more measurable metrics were agreed upon at a meeting convened by UNAIDS in 2017.^8^ These metrics include percentage reduction in HIV incidence (75% reduction set as the threshold for 2010–20), percentage reduction in AIDS-related deaths (75% reduction set as the threshold for 2010–20), the ratio of the number of incident cases to prevalent cases (incidence-to-prevalence ratio [IPR] set to a threshold of <0·03), and the ratio of number of incident cases to deaths among people living with HIV (incidence-to-mortality ratio [IMR] set to a threshold of <1·0; appendix, section 2.2, p 4). Together, these metrics can be used to provide a current epidemiological picture of HIV, but to our knowledge they have not been measured for all countries and territories.

**Research in context**

**Evidence before this study**

The Sustainable Development Goals aim to eliminate HIV and AIDS as a public health threat by 2030. HIV burden estimates are produced yearly by the Global Burden of Diseases, Injuries, and Risk Factors Study (GBD) in all countries, and UNAIDS in select countries, to help monitor progress. GBD 2017 assessed HIV and AIDS incidence, prevalence, and mortality, and coverage of antiretroviral therapy for 195 countries and territories. Although there are well documented declines in the generalised sub-Saharan African epidemic, case studies highlight acceleration of HIV incidence in key populations, including men who have sex with men, sex workers, and people who inject drugs. Against this changing epidemic, revised metrics and thresholds to measure progress related to incidence, prevalence, and deaths have been proposed by UNAIDS. These include percentage change in the number of incident cases and deaths since 2010 (>75% decrease), the incidence to prevalence ratio (<0·03), and the incidence to mortality ratio (<1·0).

**Added value of this study**

In the 2019 iteration of the GBD, we added nine new countries and territories, and updated HIV treatment and prevalence data. We also implemented a new model that allowed estimation of a transmission rate from age and sex specific HIV prevalence surveys in high-burden settings instead of relying on aggregate information. Additionally, we implemented a new method to account for bias in underlying antenatal care clinic-sentinel surveillance data that accounts for site location. We describe sex differences, and, for the first time, assess progress according to established metrics. Incident cases and deaths in males and females approached parity in 2019, reflecting declines in the female-dominated epidemic in sub-Saharan Africa. Despite this progress, countries and territories were not on track to meet thresholds related to reductions in incidence, prevalence, incidence-to-mortality ratio (IMR), or incidence-to-prevalence ratio (IPR).

**Implications of all the available evidence**

Sub-Saharan Africa continues to have the greatest burden of HIV, although our findings corroborate increasing concern about trends in HIV incidence outside this region. Although declines in incident cases and deaths in sub-Saharan Africa drove sex parity globally, challenges in engaging at-risk groups, including young women and key populations, are known in sub-Saharan Africa and other regions. These communities could be of increasing relative importance in curbing HIV in the future. Progress was inconsistent across regions and did not meet proposed thresholds. Decomposing the IMR and IPR in future analyses will allow policy makers to better understand the patterns in progress and design more targeted and hopefully increasingly-successful programmes.

In addition to assessing broad trends, understanding changes over time in the sex-specific burden of the HIV epidemic is crucial. Changes in the relative burden for males and females are evident, although research on this has been scarce.^9^, ^10^, ^11^ Although in sub-Saharan Africa women face a disproportionate burden,^11^ males in Europe and the USA faced a concentrated epidemic early on. In southeast Asia, concurrent epidemics among men who have sex with men (MSM), female sex workers, and people who inject drugs emerged.^12^ The HIV burden is also emerging in the super-regions of north Africa and the Middle East, and central Europe, eastern Europe, and central Asia, where incidence is rising among certain populations, such as MSM.^13^, ^14^ Understanding the sex-specific burden will provide better insight into current and future populations at risk.

Renewed focus on understanding differing HIV burden and trends among populations is necessary to achieving global targets for eliminating HIV as a public health threat by 2030. This study uses results from the Global Burden of Diseases, Injuries, and Risk Factors Study (GBD) 2019 to assess HIV burden in 204 countries and territories and across seven GBD super-regions from 1990 to 2019. The research aims to systematically assess trends in the HIV burden by sex and to measure progress towards SDGs. This manuscript was produced as part of the GBD Collaborator Network in accordance with GBD protocol.

## Methods

### Overview

GBD is a systematic, scientific effort to quantify the comparative magnitude of health loss caused by diseases and injuries by age, sex, and location over time. Compared with our previous iteration,^15^ GBD 2019 included nine additional locations, for a total of 204 countries and territories, and provided estimates from 1990 to 2019. The study also included a new model, the estimation and projection package age-sex model (EPP-ASM). The conceptual and analytical framework for GBD 2019, its hierarchy of causes, and detailed methods have been published elsewhere.^16^, ^17^, ^18^ Here we describe the specific methods used in GBD 2019 for analysing the burden of HIV (appendix, section 2.1, p 4). The study is compliant with the Guidelines for Accurate and Transparent Health Estimates Reporting (also known as GATHER);^19^ data and code for GBD 2019 HIV estimation process are available online. As part of GBD, seven super-regions were defined as follows: high income; sub-Saharan Africa; southeast Asia, east Asia, and Oceania; south Asia; north Africa and the Middle East; Latin America and the Caribbean; and central Europe, eastern Europe, and central Asia (appendix, section 2.3, figure S2, p 6).

### Modelling strategy

We grouped countries and territories on the basis of availability of different types of data to use unique modelling strategies that capitalise on the best available data in each country. Group 1 included countries and territories with HIV-prevalence data from antenatal care clinics or representative population-based seroprevalence surveys (ie, 48 countries in total, including several in sub-Saharan Africa and in the Dominican Republic, Haiti, India, Papua New Guinea, and Sudan). Group 2 included the remaining 156 countries, which generally had data on HIV deaths, except for 33 countries with no data on HIV deaths. The groups were further divided on the basis of peak prevalence with or without registration-data completeness (appendix, section 2.3, figure S1, p 6).

To estimate sex-specific HIV burden and trends for countries and territories in group 1, we improved the published version of EPP-ASM.^20^ To augment this model, we first developed and included an offset term applied to antenatal care data that accounts for site location. This term captures the difference between the prevalence in a given antenatal care-site year and the national prevalence in that year (appendix, section 2.5.2, p 10). Second, to obtain results for all ages, we developed and implemented a paediatric model (for those aged <15 years) within EPP-ASM; the published version only provided results for adults. This model was based on the paediatric component originally in Spectrum,^21^ the model used by many national programmes and UNAIDS to estimate the annual status of the HIV epidemic, incorporating child-treatment inputs and demographics. Finally, we derived priors for the incidence-rate ratios that were used to split larger age groups into smaller ones within the population projection. Simple linear models of observed survey prevalence against year informed our priors for the change of ratios over time and intercepts (appendix, section 2.5.2, p 11).

India was a modelling exception to other group 1 countries because we sought to use available data from antenatal care sentinel surveillance and from the sample registration system (SRS) on HIV deaths. We used an in-house version of the original estimation and projection package^22^ to estimate incidence and prevalence on the basis of data on antenatal care. We then used data on SRS deaths to inform age and sex patterns in incidence and deaths via cohort incidence bias adjustment (CIBA). Spectrum was used for final estimates.

For all countries in group 2, we developed CIBA with a modified version of Spectrum to estimate age and sex-specific HIV burden and trends. CIBA is a demographic cohort model that addresses the general scarcity of reliable data on incidence that could provide a gold-standard incidence to use as input for the Spectrum model. CIBA instead aims to derive a plausible incidence from more-reliable data on HIV deaths. We selected the incidence to input from estimates available as part of public-use files or the incidence estimated as part of a past GBD cycle that best minimised the ultimate bias between data from Spectrum deaths and reported deaths. To create an adjustment factor, we determined the ratio of Spectrum deaths to vital statistics deaths for each year. Age and sex specific incidence cohorts were defined by year of infection. The cohort that would have produced those deaths was scaled by this factor using the cohort-survival estimates derived from Spectrum (appendix, section 2.5.4, p 12). We provide a flowchart and full description of models, improvements, and additional adjustments (appendix, section 2.5, figure S3, p 9).

### Input data

Country-specific inputs that are common across the modelling strategies included data on demographic estimates and intervention coverage reported to UNAIDS, such as ART and prevention of mother-to-child transmission. Rates of disease progression and HIV-free mortality (ie, the expected background mortality not caused by HIV), required for both models were also inputs for all countries and territories.^18^ Model-specific inputs for countries and territories in group 1 for which EPP-ASM was used included population-representative HIV seroprevalence surveys and antenatal care data that were also adjusted to be population representative. For countries and territories in group 2, we used adjusted vital registration data (appendix, section 2.4, p 7).

### Estimating uncertainty

All the models were run 1000 times to create 1000 draws from which 95% uncertainty intervals (UIs) were derived, with the 2·5th and 97·5th percentile draws as the upper and lower bounds. On-ART and off-ART mortality were modelled separately. To propagate uncertainty, one draw was randomly selected each time EPP-ASM and Spectrum were run. We drew treatment inputs from a uniform distribution on each draw to ensure adequate uncertainty was captured.

### Assessing epidemic trends and progress

Sex-specific burden was assessed using the ratio of male-to-female incident cases, deaths, and people with HIV. Progress was measured using the UNAIDS metrics.^8^ We derived the percentage change in number of incident cases and deaths using case counts in 2019 versus 2010.

$${\text{Percent change}}_{\text{c}}=\frac{\left({\text{y}}_{2019}{-\text{y}}_{2010}\right)\times 100}{{\text{y}}_{2010}}$$

Y is either the total number of incident cases or the total number of deaths and c is the location. The IPR and IMR for 2010 and 2019 were also derived. IPR was measured using incident cases for all ages and both sexes combined over prevalent cases for all ages and both sexes combined. The IMR for 2010 and 2019 were measured as the number of incident cases over the total number of deaths from any cause among people living with HIV. Uncertainty for each measure was similarly derived using draw-level results from the modelling processes.

### Ethical approval

The University of Washington Institutional Review Board Committee approved GBD 2019 (approval number STUDY00009060). The study is approved until Dec 2, 2021.

### Role of the funding source

The funders of the study had no role in study design, data collection, data analysis, data interpretation, or writing of the report.

## Results

**Figure 1:**
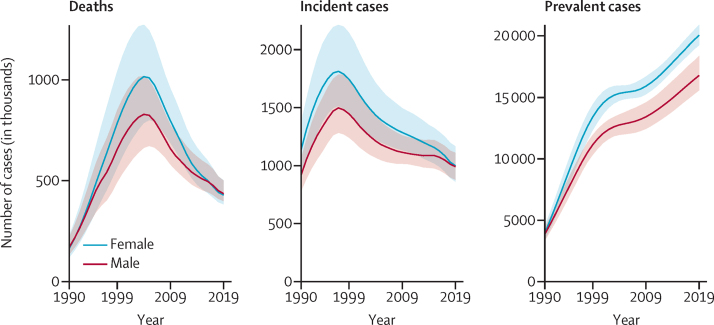
Global HIV burden 1990–2019

36·8 million (95% UI 35·1–38·9) people were living with HIV in 2019. Of these, 16·8 million (15·6–18·4) were male, and 20·1 million (19·3–20·9) were female. Over the 1990–2019 study period, the number of incident cases peaked globally in 1997 at 3·3 million (95% UI 2·8–4·0), whereas deaths peaked later, in 2004, at 1·8 million (1·5–2·2; figure 1). These estimates varied greatly by country and territory (appendix, section 2.7, figures S4A–C, pp 16–17), although sub-Saharan Africa had the highest number of incident cases, deaths, and people living with HIV in every study year. This region had 83·3% (95% UI 77·8–87·5) of the global number of incident cases, 78·5% (73·4–83·5) of the global number of deaths, and 74·1% (67·1–80·0) of the global number of people living with HIV in 1990. These numbers declined to 64·8% (58·6–70·4), 74·0% (70·7–77·2), and 70·7% (66·8–73·8), respectively, by 2019 (appendix, section 2.7, figure S5, p 17).

**Figure 2:**
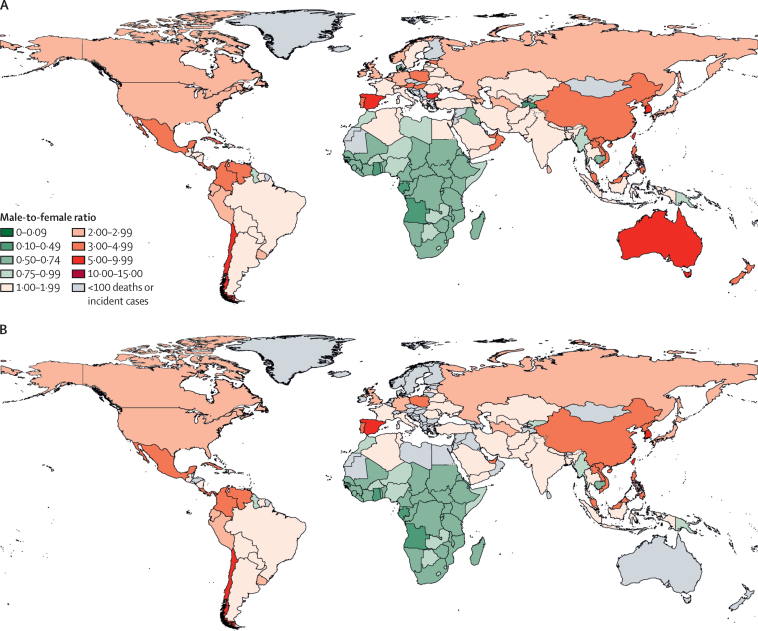
Male-to-female sex ratios for 204 countries and territories in 2019 Incident cases (A). Deaths (B) Countries and territories with fewer than 100 incident cases or deaths in 2019 were excluded from this calculation because of small burdens that drove high ratios that could not easily be interpreted.

As of 2019, prevalent cases were continuing to rise globally, while incident cases and deaths were declining. Between 1990 and 1999, the global number of people living with HIV increased by an annual mean of 13·7% (95% UI 12·6–14·8) per year compared with 2·4% (2·0–2·7) per year in 2010–19. For incident cases, the fastest decline globally occurred in the decade between 2000 and 2009 (annualised rate of decline 2·7%, 95% UI 2·0–3·4), which came after the fastest decade of acceleration, between 1990 and 1999 (annualised rate of increase 5·1%, 3·9–6·5). By 2010–19, the number of incident cases also declined at a slower annual rate than deaths (mean decrease per year of 1·8%, 0·8–2·7, *vs* annual decrease of 5·2%, 4·5–5·7; figure 2).

**Figure 3:**
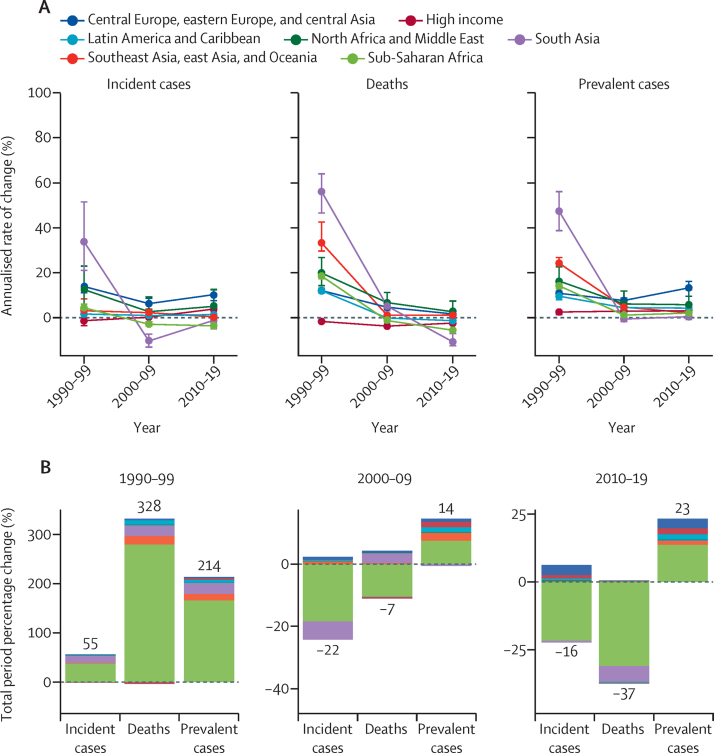
Change in HIV burden by GBD super-region for both sexes combined, by decade, from 1990 to 2019 (A) Annualised rate of change: derived as the mean yearly percentage change averaged over the given time period. (B) Total percentage change: the labelled numbers are the global percentage change in each measure over the given time period. Each bar shows the proportion and direction of change attributable to each super-region. GBD=Global Burden of Diseases, Injuries, and Risk Factors Study.

The global numbers of male and female HIV incident cases and deaths approached parity in 2019, whereas prevalent cases remained disparate. In 2019, there were 0·99 new infections (95% UI 0·91–1·10) among males for every new infection among females, 1·02 HIV deaths (0·95–1·10) among males per HIV death among females (figure 3), and 0·84 males with HIV (0·78–0·91) per female with HIV (appendix, section 2.7, figure S5, p 17).

In 2019, sex differences varied considerably by super-region (appendix, section 2.7, figure S5, p 17). Ratios were the most skewed towards females in sub-Saharan Africa, at 0·68 new infections (95% UI 0·63–0·75) among males per infection among females and 0·84 deaths (95% UI 0·77–0·92) among males per death among females. High-income super-regions had the highest ratios of new infections among males per female infection (2·42, 2·11–2·76) and deaths among males to deaths among females (2·83, 2·79–2·87).

By contrast to deaths and incident cases, the global number of males with HIV remained lower than the number of females with HIV in 2019. The global number of females with HIV increased faster than that of males with HIV between 1990 and 1999, at 14·7% (95% UI 13·4–16·1) per year versus 12·5% (11·5–13·8) per year. Between 2010 and 2019, however, females with HIV increased by a mean of 2·4% (2·0–2·8) per year, which was similar to the increase in males with HIV (increase per year of 2·3%, 2·0–2·7). At the super-region level, patterns in prevalent-case sex ratios were similar to those of incident cases and deaths (appendix, section 2.7, figures S6–8, pp 18–19).

The global number of HIV incident cases declined by 16·10% (95% UI –22·31 to –8·07) between 2010 and 2019, a change from 2·4 million (95% UI 2·2 to 2·7) new infections in 2010 to 2·0 million (1·8 to 2·3) new infections in 2019. Between 2010 and 2019, the number of HIV deaths declined by 36·75% (–39·95 to –32·64), a change from 1·4 million deaths (1·2 to 1·6) in 2010 to 864 000 deaths (786 000 to 996 000) in 2019. Neither incidence nor deaths approached the desired 75% reduction target.

Between 2010 and 2019, the decline in incident cases was driven by trends in sub-Saharan Africa, where incident cases fell by 28·50% (95% UI 19·58 to 35·43). On the other end of the spectrum was central Europe, eastern Europe, and central Asia, where incident cases increased by 107·45% (72·66 to 139·13) from 2010 to 2019, and the high-income super-region, where incident cases increased by 35·65% (3·38 to 55·91; table 1). We present country-level findings in a supplementary table (appendix, section 2.7, table S2, p 31).

**Table 1:** UNAIDS progress metrics by GBD super-region in 2010 and 2019

	**IMR, 2010**	**IMR, 2019**	**IPR, 2010**	**IPR, 2019**	**Percentage change in deaths, 2010–19**	**Percentage change in incidence, 2010–19**
Global	1·59 (1·53 to 1·65)	1·94 (1·76 to 2·12)	0·08 (0·07 to 0·09)	0·05 (0·05 to 0·06)	−36·75% (−39·95 to −32·64)	−16·10% (−22·31 to −8·07)
Central Europe, eastern Europe, and central Asia	2·82 (2·41 to 3·29)	4·76 (3·96 to 5·78)	0·15 (0·12 to 0·19)	0·11 (0·09 to 0·12)	6·85% (5·10 to 8·57)	107·45% (72·66 to 139·13)
High income	3·29 (2·45 to 4·17)	3·91 (2·65 to 4·98)	0·04 (0·03 to 0·05)	0·04 (0·03 to 0·05)	−20·26% (−21·02 to −19·55)	35·65% (3·38 to 55·91)
Latin America and Caribbean	2·25 (2·07 to 2·49)	2·61 (2·31 to 3·04)	0·09 (0·08 to 0·11)	0·07 (0·06 to 0·08)	−12·61% (−18·36 to −6·92)	11·75% (−1·82 to 23·47)
North Africa and Middle East	1·97 (1·27 to 3·18)	2·37 (1·48 to 4·19)	0·13 (0·10 to 0·17)	0·12 (0·08 to 0·17)	24·06% (−14·03 to 81·80)	52·40% (−2·87 to 172·14)
South Asia	0·73 (0·54 to 0·95)	1·40 (0·90 to 1·94)	0·06 (0·04 to 0·08)	0·05 (0·03 to 0·07)	−60·25% (−66·18 to −44·03)	−13·77% (−40·18 to 33·70)
Southeast Asia, east Asia, and Oceania	1·87 (1·62 to 2·23)	1·83 (1·6 to 2·19)	0·08 (0·07 to 0·01)	0·07 (0·06 to 0·08)	5·96% (−8·69 to 18·17)	4·91% (−7·40 to 16·76)
Sub-Saharan Africa	1·51 (1·46 to 1·57)	1·63 (1·44 to 1·82)	0·08 (0·07 to 0·01)	0·05 (0·04 to 0·06)	−39·73% (−42·36 to −36·49)	−28·50% (−35·43 to −19·58)

Data in parentheses are 95% uncertainty intervals. The IPR threshold was set as less than 0·03, and the threshold for the IMR was set to less than 1·0. The incidence and deaths threshold was at least a 75% reduction since 2010.^8^ GBD=Global Burden of Diseases, Injuries, and Risk Factors Study. IMR=incidence-to-mortality ratio. IPR=incidence-to-prevalence ratio.

Almost all super-regions contributed to the global decrease in deaths between 2010 and 2019, or showed minimal change. The largest declines were again in south Asia and sub-Saharan Africa. Deaths in south Asia declined by 60·25% (44·03 to 66·18) from 2010 to 2019, whereas deaths in sub-Saharan Africa declined by 39·73% (36·49 to 42·36). Two super-regions, the high-income super-region and Latin America and the Caribbean, saw smaller declines, whereas the other three super-regions had modest increases (appendix, section 2.7, table S1, p 19, for country and super-region results). We present the number of deaths in each super-region for each decade (table 2).

**Table 2:** Number of deaths, incident cases, and prevalent cases (thousands) globally and by GBD super-region, for both sexes combined, in 1990, 2000, 2010, and 2019

	**1990**		**1999**		**2009**		**2019**	
	Males	Females	Males	Females	Males	Females	Males	Females
**Global**								
Deaths	171 (135–221)	165 (121–230)	656 (493–850)	785 (566–1042)	658 (580–776)	796 (687–956)	435 (399–500)	428 (383–502)
Incident cases	920 (781–1060)	1140 (917–1370)	1450 (1240–1740)	1740 (1460–2140)	1110 (1020–1260)	1290 (1130–1490)	991 (878–1110)	999 (862–1170)
Prevalent cases	3870 (3350–4400)	3960 (3400–4550)	11 100 (10 300–11 900)	13 400 (12 300–14 400)	13 400 (12 400–14 600)	15 900 (15 300–16 700)	16 800 (15 600–18 400)	20 100 (19 300–20 900)
**Central Europe, eastern Europe, and central Asia**								
Deaths	4·56 (4·5–4·62)	1·32 (1·3–1·34)	12·7 (12·5–12·9)	3·16 (3·12–3·2)	18·5 (18·3–18·7)	6·69 (6·59–6·79)	19·3 (19–19·7)	8·39 (8·26–8·52)
Incident cases	11 (8·62–13·7)	2·77 (2·16–3·6)	29·2 (18·8–63·2)	10·8 (6·3–22)	45·8 (38·7–55·3)	22·4 (18·4–27·2)	112 (88·1–141)	53 (42·4–68·7)
Prevalent cases	72·6 (47·9–108)	16·2 (11·1–23·5)	170 (114–252)	51·5 (31·8–83·3)	333 (232–480)	145 (98·1–212)	1020 (837–1270)	520 (420–654)
**High income**								
Deaths	34·8 (34·5–35)	5·22 (5·17–5·28)	21·2 (21·1–21·4)	6·66 (6·61–6·71)	13·6 (13·5–13·6)	4·98 (4·94–5·02)	9·89 (9·79–9·99)	3·5 (3·46–3·54)
Incident cases	76·1 (55·6–98·9)	23·4 (15·7–33·2)	55·4 (39·6–70·2)	26·9 (17·9–35·8)	58·9 (37·8–82·5)	25·6 (16–36·3)	83·7 (49–118)	34·6 (19·9–50·6)
Prevalent cases	1080 (698–1520)	227 (137–338)	1250 (733–1850)	369 (210–555)	1570 (900–2280)	569 (322–827)	2060 (1210–2960)	811 (479–1160)
**Latin America and Caribbean**								
Deaths	12·7 (11·9–14·2)	5·13 (4·31–6·7)	30·1 (27–34·3)	17·5 (14·2–21·5)	29·9 (28·3–32·4)	17·8 (16–20)	27·0 (25·5–29·4)	14·9 (13·4–17)
Incident cases	59·2 (50·8–67·9)	34 (27·3–41·8)	64·1 (54·2–72·1)	43·3 (35·6–49·4)	72·3 (65–79·5)	42·9 (36·5–49·8)	89·9 (78·5–107)	44·8 (38–52·8)
Prevalent cases	247 (188–313)	115 (84·8–149)	505 (401–621)	306 (259–349)	771 (665–889)	483 (426–537)	1200 (1030–1410)	686 (603–772)
**North Africa and Middle East**								
Deaths	0·406 (0·183–0·969)	0·313 (0·145–0·725)	1·87 (0·971–3·92)	1·78 (0·909–3·62)	3·67 (2·04–7·68)	3·6 (2·17–6·72)	4·72 (2·55–9·97)	4·72 (2·88–9·12)
Incident cases	1·98 (0·765–4·88)	2 (0·62–5·2)	5·69 (2·89–12·4)	5·72 (2·82–10·3)	7·7 (4·01–16·5)	7·44 (3·76–13·8)	11·8 (5·12–30)	12·3 (4·51–34)
Prevalent cases	9·23 (5·44–18)	7·16 (3·34–16·8)	31·4 (16–66·5)	31·9 (14·3–62·7)	60·1 (33·8–126)	58 (33–102)	103 (54·9–214)	101 (49·4–206)
**South Asia**								
Deaths	0·812 (0·445–1·71)	0·619 (0·43–1·04)	46·5 (34·3–64·6)	29·8 (22·5–40·7)	81 (69·8–96·9)	64·8 (54·2–77·8)	28·8 (22·8–47·3)	23·2 (18·8–35)
Incident cases	19 (7·51–38·8)	9·64 (3·89–19·4)	187 (148–231)	136 (109–167)	59·8 (38·3–91·2)	44·8 (29·1–68·3)	50·1 (28–94·7)	37·8 (22·1–66·5)
Prevalent cases	41·2 (23·6–74)	19·5 (11·4–34·3)	1100 (970–1250)	729 (644–822)	1100 (978–1290)	805 (718–911)	1130 (975–1440)	805 (700–984)
**Southeast Asia, east Asia, and Oceania**								
Deaths	3·28 (1·84–4·56)	1·73 (1·04–2·23)	41·2 (32·5–46·7)	21·6 (17·4–25·1)	49·1 (44·8–55·3)	26·5 (22·4–32·5)	53·8 (47·5–64·1)	26·0 (21·4–33·2)
Incident cases	69·3 (46·6–95·4)	30·9 (20·7–43·8)	77·4 (66·3–98)	48·5 (38·1–60·1)	110 (94·1–138)	54·6 (46·2–66·6)	120 (92·9–164)	49·2 (38·8–65·9)
Prevalent cases	134 (100–174)	58·4 (43·8–76·8)	769 (616–958)	441 (358–540)	1210 (941–1640)	707 (578–871)	1540 (1170–2260)	839 (675–1100)
**Sub-Saharan Africa**								
Deaths	115 (80–163)	151 (107–214)	502 (363–669)	704 (500–945)	462 (395–566)	672·0 (575–817)	292 (254–353)	348 (304–414)
Incident cases	683 (526–849)	1040 (805–1270)	1030 (835–1300)	1470 (1200–1840)	760 (658–900)	1090 (949–1280)	524 (436–640)	767 (636–930)
Prevalent cases	2290 (1900–2710)	3520 (2930–4070)	7320 (6480–8080)	11500 (10300–12500)	8390 (7950–8930)	13 200 (12700–13700)	9740 (9210–10 300)	16 300 (15 600–16 900)

Data in parentheses are 95% uncertainty intervals. GBD=Global Burden of Diseases, Injuries, and Risk Factors Study.

At the national level, 40 (87%) of 46 countries in sub-Saharan Africa saw declines in deaths. The decline was greatest in Burundi (percentage change in deaths for 2010–19 was –72·77, 95% UI –77·50 to –65·43). In central Europe, eastern Europe, and central Asia, nine of 29 countries had more HIV deaths in 2019 than 2010. Only Georgia had an increase greater than 100%, although this was driven by low absolute numbers of deaths, with an increase of 174·52% (156·54–195·18), from eight deaths (8–9) in 2010 to 23 deaths (22–24) in 2019. In north Africa and the Middle East, 14 (67%) of 21 countries and territories had increases in mean deaths. Most notably in this region, in Iran, deaths increased by 108·04% (52·23–189·15), from 565 deaths (486–646) in 2010 to 1180 deaths (852–1710) in 2019, whereas Turkey saw a 101·95% (60·34–144·22) increase, from 112 deaths (91–140) in 2010 to 226 deaths (182–265) in 2019. In southeast Asia, east Asia, and Oceania, deaths increased faster than incident cases (table 2), and although 12 countries showed declines over the decade, the remaining 22 countries had increases in deaths (appendix, section 2.7, table S1, p 19).

The global HIV IPR (number of new infections per person with HIV) was 0·05 (95% UI 0·05–0·06) in 2019, compared with 0·08 (0·07–0·09) in 2010, thus not reaching the proposed threshold of 0·03. The IMR (number of new infections per death among people with HIV) was 1·94 (1·76–2·12) in 2019 compared with 1·59 (1·53–1·65) in 2010, also not reaching the threshold of 1·0 (table 1).

Progress towards achieving these UNAIDS benchmarks was inconsistent between the different super-regions. In 2010, the high-income super-region had the highest IMR, at 3·29 (2·45–4·17), whereas central Europe, eastern Europe, and central Asia had the highest in 2019 (4·76, 3·96–5·78). The lowest IMR in both 2010 and 2019 occurred in south Asia (0·73, 0·54–0·95, in 2010 and 1·40, 0·90–1·94, in 2019; table 1). By contrast to its high IMR, the high-income super-region had the lowest IPR in 2010 and 2019 (both years 0·04, 0·03–0·05), whereas north Africa and the Middle East (0·12, 0·08–0·17) and central Europe, eastern Europe, and central Asia (0·11, 0·09–0·12) had the highest IPRs in 2019 (table 1).

**Figure 4:**
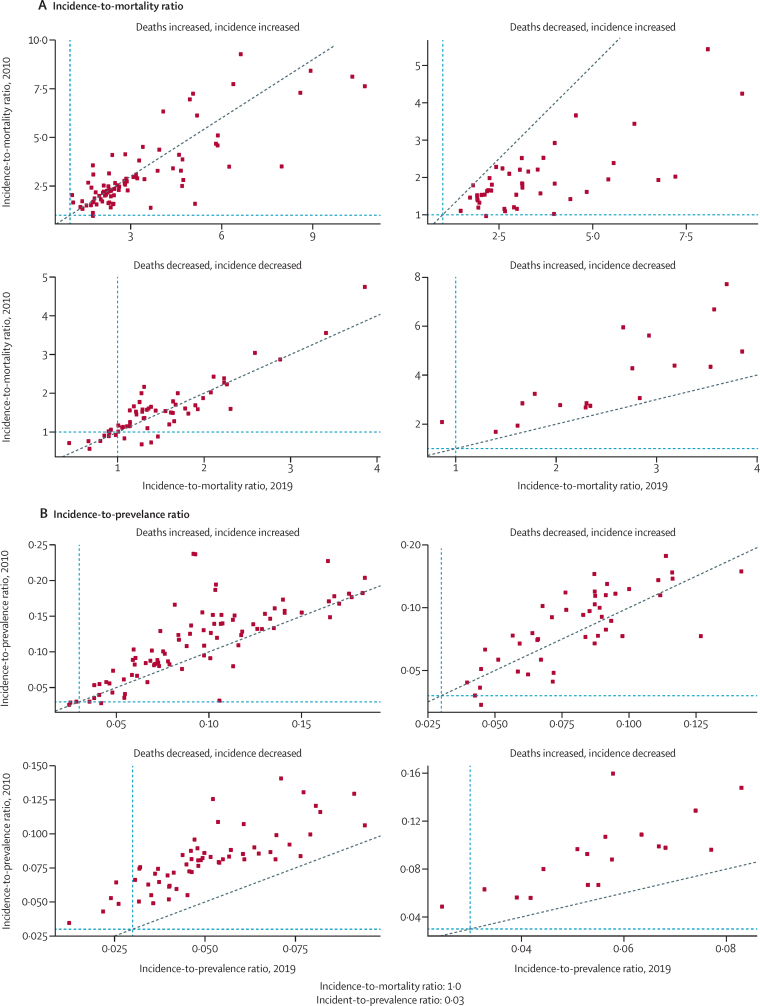
Incidence-to-mortality and incidence-to-prevalence ratios for 204 countries and territories in 2010 versus 2019, and direction of change from 2010 to 2019 The blue dotted lines represent UNAIDS thresholds (<1·0 for incidence-to mortality ratio and <0·03 for incidence-to-prevalence ratio). Points to the left of the vertical line met the threshold in 2019, whereas points below the horizontal line met the threshold in 2010. Countries and territories are sorted into a directional change matrix for deaths and incidence on the basis of changes between 2010 and 2019.

Changes in IMRs and IPRs were driven by different trends in the underlying measures within different countries (figure 4). The highest IMRs in 2019 were found primarily in locations where deaths decreased and incident cases increased since 2010 (nine of the ten countries and territories with the highest IMRs), whereas the lowest IMRs were in locations where both deaths and incident cases decreased (nine out of the ten with the lowest IMRs; appendix, section 2.7, table S5, p 60). Seven of the ten locations with the lowest IPRs in 2019 had both decreasing deaths and incident cases, whereas the remaining three locations had decreasing deaths and increasing incident cases. The top ten locations with the highest IPRs had increases in both deaths and incident cases between 2010 and 2019 (appendix, section 2.7, table S4, p 51).

## Discussion

Globally, the number of HIV incident cases and deaths decreased over the past two decades, while the gap between incident cases and deaths in males and females shrank. Because of the high prevalence of HIV among females in sub-Saharan Africa, the number of people living with HIV remained higher in females than in males globally. The decreases in incident cases and deaths did not achieve a 75% decrease between 2010 and 2019; nevertheless, the IPR decreased between 2010 and 2019, approaching the UNAIDS benchmark of 0·03.^8^ The IMR, however, increased in nearly all super-regions over the same period, and no super-region approached the benchmark of 1·0. At the same time, the changes to IMR and IPR reflected different underlying trends in incidence and mortality. Our findings point towards an evolving epidemic and the need to consider alternative metrics to IMR and IPR for a complete picture of the epidemic to inform policy.

Large declines in incidence and deaths in sub-Saharan Africa drove growing global sex parity in these two measures. Females have a disproportionate HIV burden in sub-Saharan Africa because of vulnerabilities ranging from younger sexual debut, sexual transactions with older men, and sexual violence,^23^ to biology that makes it easier for men to transmit to women, particularly when women are young.^24^ Progress in sub-Saharan Africa coincided with global health funding available for HIV in the 2000s,^25^ ART scale-up,^26^ HIV/AIDS programmes that improved health-service delivery and infrastructure,^27^ and prevention efforts.^28^, ^29^ Despite the progress, sub-Saharan Africa still shares an overwhelming burden of HIV. The region does not meet the thresholds under study, and global investments have plateaued.^25^ More granular work has also revealed heterogeneous patterns and changing HIV demographics within countries^30^, ^31^ and the potential for subnational variation,^32^ and highlights the ongoing dual burden with tuberculosis (unpublished). Elsewhere, males have a higher HIV burden, including in central Europe, eastern Europe, and central Asia and the high-income super-region, where the number of incident cases increased and the IMRs were highest in 2019. Injectable drug use has driven transmission through needles in eastern Europe and central Asia.^33^

The ongoing epidemic in Africa, incidence growth, and failure to meet progress targets corroborate calls for the increasing importance of surveillance and engagement strategies targeting at-risk groups, including key populations. Although we did not seek to model key populations specifically, our finding, for instance, of sex parity in HIV burden in north Africa and the Middle East could be masking a hidden male epidemic thought to exist among MSM related to missing data and low testing.^14^ In sub-Saharan Africa, prevention efforts have not had great success in reaching, in particular, adolescents,^34^ and key populations still face higher HIV risk and prevalence,^35^ like elsewhere.^12^, ^36^, ^37^, ^38^ Pre-exposure prophylaxis (PrEP) was associated with impressive incidence reductions, for example in MSM in Australia,^39^ but PrEP often does not reach the entirety of the population it could target.^40^, ^41^ Reducing the stigma and marginalisation that lower engagement in HIV prevention and treatment are thus essential to achieve global targets.^42^, ^43^ Historically, affected communities initiated or propagated behavioural change that drove down HIV. The Sonagachi HIV/AIDS intervention in India, for instance, demonstrated the key role of brothels in its success among female sex workers.^44^ Targeted government investment and programmes have also successfully supported reducing HIV infections in key populations.^45^ For example, the Thai government almost managed to eliminate mother-to-child transmission through extensive monitoring, prevention, and treatment policy.^46^ Failing to identify and engage at-risk populations inhibits the overall potential for progress.

To assess progress, metrics apart from IMR and IPR that do not rely primarily on changes to people with HIV might become increasingly relevant. The number of people with HIV can increase because of increasing incident cases or decreasing number of deaths as people live longer. Advances in and access to treatment have meant that life expectancies of people with HIV have approached those without HIV.^47^ An IMR of greater than 1·0, indicating a growing number of people with HIV, could reflect progress as early deaths decline. Galvani and colleagues^48^ also underscored scenarios in sub-Saharan Africa in which negative or positive changes could result in non-intuitive IMRs and IPRs. Relying on the size of the population of people with HIV could thus mask needed policy efforts that address the new challenges opened by longer lifespans for people with HIV.^49^ The growing number of people with HIV in the long term also underscores the importance of reducing the number of incident cases to constrain long-term ART costs for decades to come.^50^ These factors are reflected in the US President's Emergency Plan for AIDS Relief (PEPFAR), which suggests that IMR only be used in settings with high ART coverage.^51^ As such, further decomposing changes in prevalence and the numerators and denominators of IMR and IPR becomes essential, rendering the metrics less useful in isolation.

The COVID-19 pandemic has the potential to complicate progress in controlling HIV epidemics, although research on this issue is scarce. People living with HIV do not seem to have higher susceptibility to severe COVID-19, although vulnerable social status in this population might put them at higher risk than the general population.^52^ There are also potential challenges related to treatment services, including timely access to care, continuing on treatment, and HIV testing.^53^ One survey in China in February, 2020, found that about 64% of people living with HIV in Hubei province reported difficulty accessing antiretrovirals because of barriers.^54^ In Kenya, there were concerning trends in turnaround times for HIV testing, numbers of tests, and clinical restructuring for COVID-19 mitigation.^55^ There are, however, important efforts underway to mitigate these disruptions. In China, the Chinese National Centre for AIDS/STD Control and Prevention has worked to ensure people with HIV can collect medications.^56^ In Kenya, the Ministry of Health allowed early refills for an extra 3 month supply of antiretrovirals.^57^ Although these are just case studies, broader efforts through WHO, PEPFAR, UNAIDS, and the Global Network of People Living with HIV are working to this end.^58^, ^59^, ^60^, ^61^

Comparing GBD HIV estimates with those published by UNAIDS is useful. For the year 2019, we estimated 36·8 million (95% UI 35·1–38·9) people with HIV, 1·99 million (1·76–2·26) incident cases, and 864 000 (786 000–996 000) deaths, compared with the UNAIDS 2020 published estimates of 38·0 million (31·6–44·5) people with HIV, 1·7 million (1·2–2·2) incident cases, and 690 000 (500 000–970 000) deaths globally in 2019.^62^ Although the GBD estimate of people with HIV is the most similar, higher incidence and deaths are a reflection of several important differences in GBD methods compared with UNAIDS: we did an independent systematic review and synthesis model for on-treatment mortality and progression parameters that were important inputs in both Spectrum and EPP-ASM (appendix, section 2.5, p 9); we incorporated the CIBA process, which involves estimating incidence with mortality data; we used GBD population, fertility, births, and migration estimates in the population projection; and the modelling strategy across groups of countries for UNAIDS is more heterogeneous, with for example the Asian Epidemic Model in southeast Asia. Further, GBD provides two important advantages unavailable from UNAIDS, which include internally consistent estimates with other causes of death and similar estimates across diseases.

This study has several limitations. The results of the cohort-incidence bias adjustment approach to incorporating vital registration data are sensitive to our initial incidence input to the first stage of Spectrum. Although we aimed to mitigate this problem by choosing the most plausible incidence option that yields deaths closest to more trustworthy vital registration data, there is a general scarcity of reliable data on incidence that could provide a gold-standard incidence input. Future work establishing the most likely incidence in a statistical framework could improve the selection of the incidence input relative to vital registration data and thus improve final burden estimates. The results are also affected by the limitations of the best data sources of countries and territories. Although GBD aims to make use of the best-available and most-recent data in different countries and territories, such data are prone to different biases related to sampling and modelling. Sex differences, for example, can be driven by modelling assumptions for which data are scarce or the dominant data source (case reports, vital statistics, or prevalence surveys) does not capture certain groups. We expect our incidence estimates might be too low in cases in which official data exclude certain population segments. Related to this issue, because of the scarcity of comprehensive data regarding HIV prevalence in gender non-conforming, intersex, and transgender individuals, a male-female binary was used in this research. Despite not having this data, HIV often disproportionately affects these populations, and this work is limited by the inability to provide estimates at a finer granularity of sex and gender identity. In countries and territories without data, our estimates rely on the assumption that regional means provide a good proxy for required treatment and other model inputs.^16^ Because countries and territories without data tend to have smaller HIV epidemics, we believe this assumption has limited consequences for regional or global estimates. Still, we might not be accurately capturing the epidemic in these locations if they are outliers in their region.

HIV incident cases and deaths decreased over the past two decades, while the number of people living with HIV continued to increase through 2019. The difference in the number of incident cases and deaths in males and females shrank whereas the number of females living with HIV remained higher than in males living with HIV in 2019. The largest decrease in incidence and deaths was in sub-Saharan Africa, where females also have a higher burden of HIV. Incidence increased the fastest in regions where HIV incidence concentrates in key populations, central Europe, eastern Europe, and central Asia, and the high-income super-region.

Although global trends in IPRs are decreasing, a sign of progress, IMRs were higher in more regions in 2019 than 2010, and did not meet benchmarks set by the UNAIDS consensus. However, as the number of people with HIV expands because of declining deaths rather than increasing incidence, IMRs and IPRs might not be sufficient for measuring HIV progress. Estimating HIV burden and trends in different populations remains crucially important to provide policy makers with information to design effective control measures.

## Data sharing

To download the data used in these analyses, please visit the Global Health Data Exchange GBD 2019 data-input sources tool at http://ghdx.healthdata.org/gbd-2019/data-input-sources.

## Declaration of interests

S Afzal reports an unpaid leadership role as General Secretary for the Pakistan Society of Community Medicine and Public Health, working for prevention and advocacy of AIDS in families of HIV patients by providing advocacy and counseling services to the families, children, and partners of patients with AIDS and screening them for HIV in Pakistan, outside the submitted work. T Bärnighausen reports research grants from the European Union (Horizon 2020 and EIT Health), German Research Foundation (also known as DFG), US National Institutes of Health (NIH), German Ministry of Education and Research, Alexander von Humboldt Foundation, Else-Kröner-Fresenius-Foundation, the Wellcome Trust, the Bill & Melinda Gates Foundation, KfW, UNAIDS, and WHO; consulting fees for KfW on the OSCAR initiative in Vietnam; and participation on a Data Safety Monitoring Board or Advisory Board through the NIH-funded study Healthy Options (Principle Investigators Smith Fawzi, Kaaya), Chair, Data Safety and Monitoring Board, German National Committee on the Future of Public Health Research and Education, Chair of the scientific advisory board to the EDCTP Evaluation, Member of the UNAIDS Evaluation Expert Advisory Committee, National Institutes of Health Study Section Member on Population and Public Health Approaches to HIV/AIDS, US National Academies of Sciences, Engineering, and Medicine's Committee for the Evaluation of Human Resources for Health in the Republic of Rwanda under the President's Emergency Plan for AIDS Relief (also known as PEPFAR), University of Pennsylvania Population Aging Research Center External Advisory Board Member; and a leadership or fiduciary role in other board, society, committee, or advocacy group, paid or unpaid as Co-Chair of the Global Health Hub Germany (which was initiated by the German Ministry of Health), all outside the submitted work. J Eaton reports institutional support for the manuscript from the Gates Foundation, UNAIDS, and National Institutes of Health; grants or contracts from WHO, Gates foundation, and UNAIDS; consulting fees from WHO; and support for attending meetings or travel from WHO and UNAIDS, all outside the submitted work. I Filip reports financial support from Avicenna Medical and Clinical Research Institute, outside the submitted work. I Iyamu reports support for attending FHI 360 HIV prevention, care, and treatment training events in Nigeria, outside the submitted work. K Krishan reports non-financial support from UGC Centre of Advanced Study, CAS II, Department of Anthropology, Panjab University, Chandigarh, India, outside the submitted work. K LeGrand reports support for the present manuscript through payment to the institution (Institute for Health Metrics and Evaluation) from the Gates Foundation. M Postma reports a leadership or fiduciary role in other board, society, committee, or advocacy group, unpaid as member of UK's JCVI. O Rezahosseini reports grants or contracts from the Research Foundation of Rigshospitalet and the A P Møller Fonden; and support for attending meetings or travel from EACS 2019 Basel, all outside the submitted work. J Salomon reports support for the present manuscript through a research grant paid to the institution (Institute for Health Metrics and Evaluation) from the Gates Foundation. J Singh reports consulting fees from Crealta/Horizon, Medisys, Fidia, Two labs, Adept Field Solutions, Clinical Care Options, Clearview Health-Care Partners, Putnam Associates, Focus Forward, Navigant Consulting, Spherix, MedIQ, UBM LLC, Trio Health, Medscape, WebMD, and Practice Point Communications, the NIH, and the American College of Rheumatology; payment or honoraria for lectures, presentations, speakers bureaus, manuscript writing, or educational events from Simply Speaking; support for attending meetings or travel from OMERACT, an international organisation that develops measures for clinical trials and receives arm's-length funding from 12 pharmaceutical companies, when traveling biannually to OMERACT meetings; participation on a Data Safety Monitoring Board or Advisory Board as a member of the FDA Arthritis Advisory Committee; a leadership or fiduciary role in other board, society, committee, or advocacy group, paid or unpaid, with OMERACT as a member of the steering committee, with the Veterans Affairs Rheumatology Field Advisory Committee as a member, and with the UAB Cochrane Musculoskeletal Group Satellite Center on Network Meta-analysis as a director and editor; and stock or stock options in TPT Global Tech, Vaxart pharmaceuticals, Charlotte's Web Holdings, and previously owned stock options in Amarin, Viking, and Moderna pharmaceuticals, all outside the submitted work. D Stein reports personal fees from Lundbeck, Takeda, Johnson & Johnson and Servier, all outside the submitted work. A Tsai reports stipend for work as Editor-in-Chief of Social Science and Medicine, Mental Health from Elsevier. All other authors declare no competing interests.
