# Correction to *Lancet HIV* 2021; 8: e633–51

**DOI:** 10.1016/S2352-3018(22)00042-X

**Published:** 2022-02-21

*GBD 2019 HIV Collaborators. Global, regional, and national sex-specific burden and control of the HIV epidemic, 1990–2019, for 204 countries and territories: the Global Burden of Diseases Study 2019.* Lancet HIV *2021; **8:** e633–51*—In this Article, the original figure 2, titled “Male-to-female sex ratios for 204 countries and territories in 2019” is in fact figure 3, and the original figure 3, titled “Change in HIV burden by GBD super-region for both sexes combined, by decade, from 1990 to 2019” is in fact figure 2. These corrections have been made to the online version as of Feb 21, 2022.

