# Supplementary material for: Global, regional, and national sex-specific burden and control of the HIV epidemic, 1990–2019, for 204 countries and territories: the Global Burden of Diseases Study 2019
Source: Lancet HIV. 2021 Sep 27;8(10):e633–51. doi: 10.1016/S2352-3018(21)00152-1 (PMC8491452; doi:10.1016/S2352-3018(21)00152-1)
Supplement: Supplementary appendix [file mmc1.pdf]

THE LANCET
HIV

Supplementary appendix

This appendix formed part of the original submission and has been peer reviewed. We post it as supplied by the authors.

Supplement to: GBD 2019 HIV Collaborators. Global, regional, and national sex-specific burden and control of the HIV epidemic, 1990–2019, for 204 countries and territories: the Global Burden of Diseases Study 2019. *Lancet HIV* 2021; **8:** e633–51.

Supplementary Appendix to “Global, regional, and national sex-specific burden and control of the HIV epidemic, 1990-2019: results from the Global Burden of Disease Study 2019”

This appendix provides further methodological detail and results for “Global, regional, and national sex-specific burden and control of the HIV epidemic, 1990-2019: results from the Global Burden of Disease Study 2019”

Portions of this appendix have been reproduced or adapted from previous Global Burden of Diseases (GBD) efforts.[1,2] References are provided for reproduced sections.

Contents

List of figures and tables 2
Figures 2
Tables 3
Section 1. Statement of GATHER compliance 3
Section 2. Methods 4
Section 2.1. Case definition 4
Section 2.2. Progress Metrics 5
Section 2.3. Country Groupings 5
Section 2.4. Input Data 7
Section 2.4.1. Common inputs 7
Section 2.4.2. Group 1 countries and territories 7
Section 2.4.3. Group 2 countries and territories 8
Section 2.5. Modeling HIV 9
Section 2.5.1. On–ART, off–ART mortality and CD4 progression parameters 9
Section 2.5.2. EPP–ASM 9
Section 2.5.3. Spectrum 11
Section 2.5.4. Cohort incidence bias adjustment (CIBA) 12
Section 2.5.5. Spatiotemporal Gaussian Process Regression (ST–GPR) 14
Section 2.5.6. Additional Adjustments 14
Section 2.6. References 15
Section 2.7. Figures and tables 16
Authors’s Contributions 61

List of figures and tables

Figures and tables are available in section 2.7.

Figures

Figure S1. HIV specific country groupings based on data availability.

Figure S2. Global Burden of Disease 2019 super regions.

Figure S3: Flow chart of modelling process

Figure S4A. Deaths due to HIV per 1000 in 2019

Figure S4B. Incident cases of HIV per 1000 in 2019

Figure S4C. Prevalent cases of HIV per 1000 in 2019

Figure S5. Percentage share of global burden of HIV incident cases (A), deaths (B) and prevalent cases (C) in each GBD super-region, by sex, 2019. Dots represent the percentage share of the global population in each super-region. GBD=Global Burden of Diseases, Injuries, and Risk Factors Study.

Figure S6. Deaths due to HIV (in thousands) from 1990 to 2019 by super region

Figure S7. Incident cases of HIV (in thousands) from 1990 to 2019 by super region

Figure S8. Prevalent cases of HIV (in thousands) from 1990 to 2019 by super region

Tables

Table S1. Number of deaths due to HIV and percent change between 2010 and 2019 for GBD super-regions and 204 countries and territories

Table S2. Number of HIV incident cases and percent change between 2010 and 2019 for GBD super-regions and 204 countries and territories

Table S3. Number of prevalent cases of HIV in 2019 for GBD super-regions and 204 countries and territories

Table S4. Incidence to prevalence ratio in 2019 for GBD super-regions and 204 countries and territories

Table S5. Incidence to mortality ratio in 2019 for GBD super-regions and 204 countries and territories

Section 1. Statement of GATHER compliance

Table 1. Checklist of information that should be included in reports of global health estimates, with description of compliance and location of information for " Global, regional, and national sex-specific burden and control of the HIV epidemic, 1990-2019: results from the Global Burden of Disease Study 2019"

#	GATHER checklist item	Description of compliance	Reference
**Objectives and funding**			
1	Define the indicators, populations, and time periods for which estimates were made.	Narrative provided in paper and methods appendix describing indicators, definitions, and populations	Main text (Methods) and methods appendix
2	List the funding sources for the work.	Funding sources listed in paper	Summary (Funding)
**Data Inputs**			
For all data inputs from multiple sources that are synthesized as part of the study:			

3	Describe how the data were identified and how the data were accessed.	Narrative description of data seeking methods provided	Main text (Methods) and methods appendix
4	Specify the inclusion and exclusion criteria. Identify all ad-hoc exclusions.	Narrative about inclusion and exclusion criteria by data type provided	Main text (Methods) and methods appendix
5	Provide information on all included data sources and their main characteristics. For each data source used, report reference information or contact name/institution, population represented, data collection method, year(s) of data collection, sex and age range, diagnostic criteria or measurement method, and sample size, as relevant.	An interactive, online data source tool that provides metadata for data sources by component, geography, cause, risk, or impairment has been developed	Online data citation tool, http://ghdx.healthdata.org/gbd-2019
6	Identify and describe any categories of input data that have potentially important biases (e.g., based on characteristics listed in item 5).	Summary of known biases by cause included in methods appendix	Main text (Methods) and Methods appendix
For data inputs that contribute to the analysis but were not synthesized as part of the study:			
7	Describe and give sources for any other data inputs.	Included in online data source tool, http://ghdx.healthdata.org/gbd-2019	Online data citation tools
For all data inputs:			
8	Provide all data inputs in a file format from which data can be efficiently extracted (e.g., a spreadsheet as opposed to a PDF), including all relevant meta-data listed in item 5. For any data inputs that cannot be shared due to ethical or legal	Downloads of input data available through online tools, including data visualization tools	Online data visualization tool, http://ghdx.healthdata.org/gbd-2019

Section 2. Methods

Section 2.1. Case definition[1]

Infection with HIV causes influenza-like symptoms during the acute period following infection and can lead to AIDS if untreated. HIV attacks the immune system of its host, leaving infected individuals more susceptible to opportunistic infections like tuberculosis. Although there are two different subtypes of HIV, HIV-1 and HIV-2, no distinction is made in our estimation process or presentation of results. For HIV, ICD 10 codes are B20-B24, C46-C469, D84.9; ICD 9 codes are 042-044, 112-118 (after 1980), 130 (after 1980), 136.3-136.8 (after 1980), 176.0-176.9 (after 1980), 279 (after 1980); and ICD9 BTL codes are B184-B185.

Section 2.2. Progress Metrics

The incidence:prevalence ratio threshold was set as 0·03, while the threshold for the incidence:mortality ratio was set to 1.0. This value assumes that people with incident cases will live approximately 33 years, thus 1/33 reflects an equilibrium where there are, “fewer than one new infection per person living with HIV over the course their infection.” Time lived with HIV varies by and within countries and social groups, however 1/33 was chosen as representative despite these disparities. The threshold for the IMR was set to one because below this, the number of PLHIV shrinks.[3]

Section 2.3. Country Groupings

Group 1 included countries and territories with HIV prevalence data from antenatal care (ANC) clinics or representative population-based seroprevalence surveys (i.e., 48 countries and territories in sub-Saharan Africa, North Africa and Middle East, South Asia, Latin America and Caribbean and Southeast Asia, East Asia, and Oceania). Group 1 was further subdivided into Group 1A with a peak of at least 0·5% prevalence, and Group 1B for countries with a peak prevalence of at least 0·25% or vital registration data completeness of greater than 65% and available ANC data (including only India, Somalia, and Sudan). Group 2 included all other countries and territories, which were further classified as Groups 2A, 2B, and 2C based on the availability and quality of vital registration data. These locations had high-quality data (Group 2A), at least some data (Group 2B), or no data on HIV-specific mortality at all (Group 2C), respectively. Quality was defined based on a star system described elsewhere.[4] We did not use ANC data in group 2 countries. Results were aggregated by super region as defined by the Global Burden of Disease study. These super regions are depicted in figure S2.

Figure S1. HIV specific country groupings based on data availability.

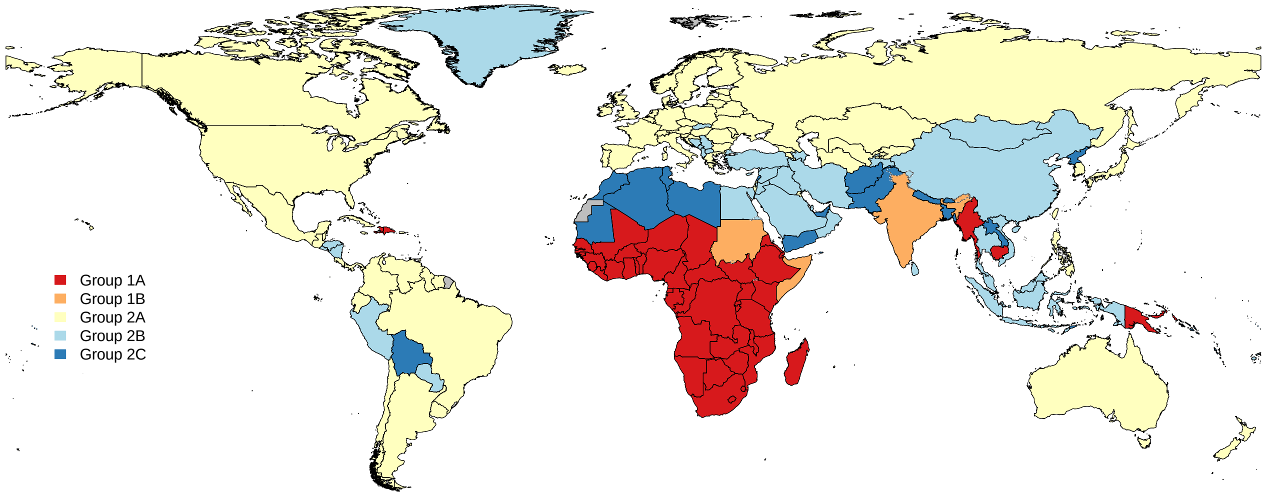

Figure S2. Global Burden of Disease 2019 super regions.

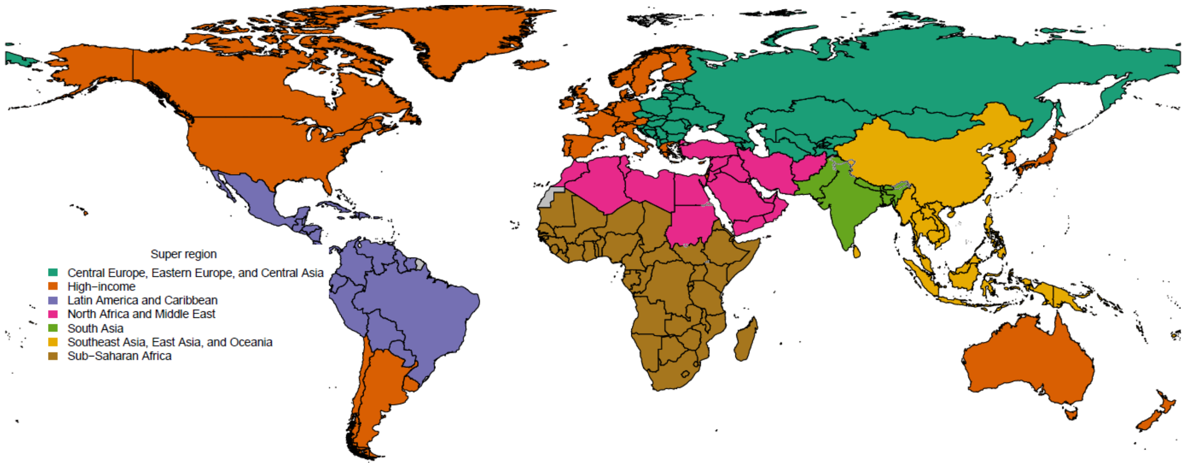

Section 2.4. Input Data

Section 2.4.1. Common inputs

Country–specific inputs common across the country groupings included demographic estimates (population, fertility, migration, and HIV–free survival rates from GBD 2019) which are detailed in the recent GBD publication.[1]

Country–specific inputs also included HIV–specific information such as intervention coverage data including ART, prevention of mother–to–child transmission (PMTCT), and ART eligibility. The files compiled by UNAIDS for their HIV/AIDS estimation process were our main source for this data. Spectrum files are often built by within–country experts with the support of UNAIDS, who publishes estimates annually on behalf of countries and only shares their Spectrum files when permission is granted. Observed treatment data were unavailable in 40 countries, in which case we used regional averages of treatment inputs. A total of 20 of these were in southeast Asia, east Asia and Oceania; nine in Latin America and Caribbean; five in north Africa and Middle East; five in the high–income super–region; and one in Central Europe, Eastern Europe, and Central Asia.

HIV mortality rates and disease progression rates were also inputs for all countries and territories, but not country–specific; mortality rates among those on ART were common to countries and territories within a region, while off–ART and CD4 progression parameters were common across all countries and territories. On–ART and off–ART data were identified through a literature searches described elsewhere and not updated for GBD 2019. Briefly, for on–ART, we used search terms "HIV," "mortality," and "antiretroviral therapy" in PubMed searches across the literature. To be included, studies must have had HIV–positive people who receive antiretroviral therapy (ART) but who were ART–naïve prior to the study. In addition, studies must report either a duration–specific (time since initiation of ART) mortality proportion or a hazard ratio across age or sex. For off–ART, we identified 13 cohort studies that included the cohorts, from which we extracted survival at each one–year point after infection. For full details please see Frank et al.[2]

Section 2.4.2. Group 1 countries and territories

Model–specific inputs for Group 1 countries and territories where EPP–ASM was used included population–based seroprevalence surveys and antenatal care (ANC) clinic data. HIV prevalence surveys were obtained from different series including the Demographic and Health Surveys (DHS), Multiple Indicator Cluster Surveys (MICS), and Population–based HIV Impact Assessment (PHIA), where HIV

biomarker testing was conducted. We used surveys sex–stratified to 5–year age groups or sex–age aggregate surveys depending on the most granular information available. ANC clinic data were obtained from public use country files released by UNAIDS. The data contained results of HIV testing in females attending antenatal care sentinel surveillance (ANC-SS).

Section 2.4.3. Group 2 countries and territories

In CIBA, we used vital registration data. These data were adjusted for incompleteness and misclassification of causes of death. Misclassification adjustments corrected the data through identifying causes of death deviating from expected age patterns of mortality in years with known HIV epidemics, and excess deaths were attributed to HIV. There are two different cause of death data sources for HIV/AIDS in China: the Disease Surveillance Point (DSP) system and the Notifiable Infectious Disease Reporting (NIDR) system. Both systems are administered by the Chinese Center for Disease Control and Prevention, but the reported number of deaths due to HIV is significantly lower in DSP. Therefore, we have used the provincial–level ratio of deaths due to HIV/AIDS from NIDR to those from DSP, choosing the larger ratio between years 2013 and 2014, and scaled the reported deaths in the DSP system.

We identified 121 countries – as well as 632 subnational locations from China, Japan, Indonesia, India, Mexico, Sweden, Philippines, Poland, Italy, the United Kingdom, Ukraine, Russia, New Zealand, Iran, Norway and the United States – with usable points of vital registration data, verbal autopsy (VA) data, or sample registration system (SRS) data. In India, Vietnam and Indonesia, we used SRS and VA data, respectively, as input mortality for CIBA. For India we extracted the resulting age–sex distribution of incidence but scaled the level to match the adult incidence rate estimated from EPP for each state.

We also used case reports from group 2 countries believed to have high quality data for case notifications, mainly countries in our high–income super region and with 4 or 5-star vital registration data. These reports were extracted from country-level reports.

Section 2.5. Modeling HIV

Figure S3: Flow chart of modelling process[1]

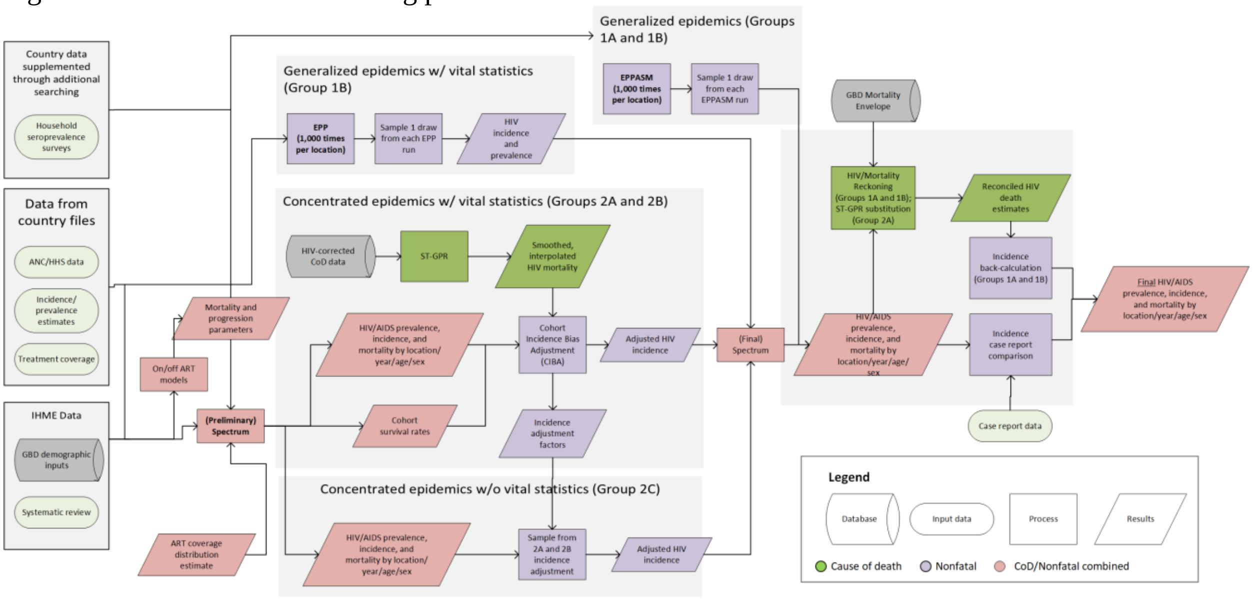

Section 2.5.1. On–ART, off–ART mortality and CD4 progression parameters

We continued to estimate on-ART and off-ART mortality by CD4 count as in the Global Burden of Disease study 2017 which is detailed elsewhere.[2] Briefly, for on ART mortality, we conducted a meta-analysis of mortality rates to synthesize age-sex-split study-level data into estimates of conditional probability of death over initial CD4 count. We modelled the data separately by duration, age, sex, and region and added a fixed effect on whether the study was conducted prior to 2002. Finally, to ensure off ART mortality rates were never lower than on-ART mortality rates, we substituted our on-ART mortality rates with those estimated off treatment in the situation that they were estimated to be higher.

Section 2.5.2. EPP–ASM[1]

The EPP–ASM is a statistical model that infers the most likely HIV transmission rate in the population from trends in the ANC and survey prevalence data.[5] A major methodological improvement in EPP-ASM is the ability to incorporate age-sex specific HIV prevalence data from household surveys in the model calibration to more accurately capture the changing demographics of the HIV epidemic. The transmission rate was assumed to follow a logistic model until 2003 and a random walk process thereafter through the last year of data (r-hybrid). Projection to 2019 was done using a random walk model based on the most likely transmission rate parameters, which were estimated before the end of the data. A population

demographic population projection model similar to Spectrum is built into EPP-ASM to produce final results.

We implemented a new approach within EPP-ASM to address selection bias resulting from temporal and geographic variation in ANC reporting. EPP-ASM has embedded approaches to adjust for the bias associated with using prevalence among ANC–site attending pregnant women to estimate prevalence among the both–sexes population. However, the embedded approach does not explicitly account for the fact that the location of the clinic in space may also drive its HIV prevalence level. For example, we might expect rural sites to be more correlated than urban sites.

To further adjust for this bias, we used an offset term that represents the difference in the prevalence among the national population and the prevalence among the female, pregnant population associated with an ANC site location. The national prevalence was adjusted for covariates including access to health facilities, malaria incidence and male circumcision, as well as a spatial random effect accounting for factors influencing prevalence that are associated with the site's geographic location. Our final strategy for estimating the likelihood of the observed ANC data was:

$$\boldsymbol{W_{st} = \varphi^{-1}(\rho_t) + \vartheta_{st} + u_s + e_{st}}$$

$$\boldsymbol{e_{st} \sim N(0, \sigma_{st}^2)}$$

$$\boldsymbol{u_s \sim N(0, \sigma_s^2)}$$

Where:

W_{st} = the probit transformed prevalence at site *s* and time *t*

ρ_t= The national prevalence adjusted to represent prevalence among pregnant women from the model simulation

ϑ_{st} = The offset term representing the difference between the adjusted prevalence in a given site–year and the adjusted national prevalence in that year

φ^{-1} = probit transformation

e_{st} = Site–specific error term

u_s = Site specific intercept

We also implemented a replica of the paediatric model that is built into Spectrum to obtain estimates for all ages because the published version of EPP–ASM was limited to adults. There are well-recognized

limitations to paediatric modelling that we were not able to address in this addition but do provide an important future direction for research. These include, for example, limiting incidence to occur only through birth and breastfeeding thus assuming no transmission until age 15. Another ongoing area of exploration as has been treatment rates for the paediatric population, for example, the variation in estimates across sources.[6]

Finally, we derived priors for the incidence rate ratios that were used to split the coarser age groups into higher resolution results within the population projection. The priors were derived using observed prevalence data from 11 countries and territories with three or more available DHS or PHIA HIV prevalence surveys since the year 2000. Three sex–stratified simple linear models were fit against year. The three outcomes were: the ratio of prevalence in the 15-24 relative to 25-34 year age group, the ratio of 35+ age group relative to 25-34, and the ratio of prevalence in 15-24 to the 25+ age group. The change over time and intercept were used as priors on the incidence rate ratios.

Section 2.5.3. Spectrum[1]

Previously, we created an exact replica of Spectrum in Python which enabled us to run thousands of iterations of the model at once on our computing cluster and allowed for more flexible input data structures. Additionally, we scaled all input values by a uniformly sampled factor between 0.9 and 1.1 to generate estimates with realistic ranges of uncertainty. For example, if treatment retention rates across CD4 categories were 0.906, 0.759, 0.787, 0.795, 0.785, 0.756, 0.813, and 0.700, we multiplied each number by an array of equivalent size that contained factors ranging from .9 and 1.1.

To allocate people initiating ART treatment across CD4 category, Spectrum determines the number of people initiating ART treatment across each CD4 category. We previously improved the basis for this distribution using survey microdata and country–level wealth information[2]. Three relevant surveys were available: Uganda AIS 2011 and Kenya AIS 2007 and 2012. These surveys conducted CD4 count measurements and include a question regarding the amount of time that an individual receiving ART had been enrolled in treatment. We built a model to translate an individual's current CD4 count and duration on treatment into CD4 count at initiation of treatment required by Spectrum. The functional form for changes in CD4 count as a function of duration on treatment was a natural spline on duration with knots at 3, 12, 24 and 36 months, and an interaction between initial CD4 count and duration. Using this model, we could predict the probability of being on treatment as a function of individual income (measured through an asset–based index), stratified by CD4 count, age, and sex. The results of this prediction were translated

into country–specific age–sex–year–CD4 count probabilities of coverage using a conversion factor between individual income and lagged distributed GDP per capita. We used stochastic frontier analysis to constrain the maximum possible coverage for a given degree of income and CD4 count. Predicted probabilities of coverage were input to Spectrum to inform the distribution, and not the overall level, of ART treatment by CD4 count.

Additional improvements to Spectrum that were made for the GBD are described elsewhere[2] and were not changed for GBD 2019.

Section 2.5.4. Cohort incidence bias adjustment (CIBA)[2]

CIBA is a demographic cohort model that allows us a way to "fit" incidence data to vital registration data in the absence of an optimisable framework. To improve the fit of this process, in GBD 2015, we restructured Spectrum to track cohorts by year of HIV infection. With this version of Spectrum we can output, among many other metrics, HIV deaths by year, age, sex, and infection cohort (defined by a common year of infection). The incidence input to this first stage of Spectrum was either the incidence estimates available from countries and territories as part of public use files or the incidence estimated as part of a past GBD cycle. The choice minimised the ultimate bias between Spectrum deaths and reported deaths data.

For every year-, age-, and sex-specific infection cohort, we calculated the share of all HIV deaths output from Spectrum observed that would occur in each year after the year of infection. For example, projecting from 1970 through 2019, we identified the cohort of men infected in 1992 at the age of 16, calculated the total number of HIV deaths in that cohort in all subsequent years through the end of 2019, and divided the annual number of deaths by that total. This showed us the distribution of deaths among that cohort over the projection period. In the most extreme case (infections in 2018), we could only produce one point of that distribution (2019), so that single value is exactly 1·0; 100% of the deaths observed in that cohort occurred in 2019.

To create an adjustment factor, we determined the ratio of Spectrum deaths to vital statistics deaths for each year. We then used these distributions of death to weigh the ratio of VR deaths to Spectrum deaths, meaning that ratios in the years where we expect the largest share of deaths were weighed most heavily. The initial size of that cohort from the normal run of Spectrum was multiplied by the sum of the

combined ratios to get a new estimate of new cases in that year/age/sex combination. We can write this method mathematically in the following way:

$$r_t = \frac{VR_t}{D_t}$$

$$\rho_t^{t-i} = \frac{d_t^{t-i}}{\sum_{k=t-i+1}^{n} d_k^{t-i}}$$

$$\alpha^{t-i} = \sum_{k=t-i+1}^{n} r_k * \rho_k^{t-i}$$

$$n_{\text{adjusted}}^{t-i} = \alpha^{t-i} * n^{t-i}$$

VR_t is the number of HIV/AIDS deaths in year t from ST–GPR, and D_t is the number of HIV/AIDS deaths from the first run of Spectrum. In the second equation, d_t^{t-i} is the number of HIV/AIDS deaths among members of infection cohort $t - i$ in year t, with $i \geq 1$, from the new, duration–tracking version of Spectrum, and n is final year of the projection. Therefore, ρ_t^{t-i} is the share of observed deaths in cohort $t - i$ that we expect to occur in year t. It follows that α^{t-i} is the weighted adjustment ratio described above, which we multiply by the estimated initial size of infection cohort $t - i$ as calculated in the first-stage Spectrum run to get the adjusted number of new cases, $n_{\text{adjusted}}^{t-i}$. This process is run separately for every sex, single–age, and draw.

CIBA allows ratios in each year after a given infection year to influence the final adjustment to incidence. The size of that influence is determined by the relative importance of that year in the cohort-year's distribution of deaths over time. The result is a new set of 1,000 draws of incidence and a set of 1,000 ratios of post–adjustment incidence to pre–adjustment incidence. We perform this adjustment using mean durations from the new version of Spectrum in order to try to shift the mean of the regular distribution of deaths.

To produce final location-, year-, age-, and sex-specific estimates of HIV incidence, prevalence, and mortality, we ran the new estimates of incidence and all previously input data through Spectrum. For group 2A, final deaths estimates were produced through ST-GPR, as described below, as vital registration data was deemed the most reliable source of deaths information.

Section 2.5.5. Spatiotemporal Gaussian Process Regression (ST–GPR)[1]

We imputed missing years of vital registration data to generate a complete time series for HIV from the estimated start year of the epidemic using ST-GPR. We analysed mortality trends using ST-GPR starting in 1981, the year that HIV was first clinically recognized the United States.[7] For ST-GPR, we adjusted the lambda (time weight) and GPR scale according to the completeness of vital registration data, with 4- and 5-star quality VR using parameters designed to follow the data more closely. We produced separate splines by country/age group, up to the peak year of death rate. We then ran a linear regression with fixed effects on region, age, and sex. Following this, we ran space-time residual smoothing, in which time, age, and space weights are used to inform smoothing of the residuals between data points and the linear regression estimate. From this process, we generated space-time estimates with the applied weights, along with the median absolute deviation (MAD) of the space-time estimates from the data. The MAD was calculated at various levels of the geographic hierarchy (eg, subnational and national), and was added into the data variance term. The data variance and space-time estimates were then analysed using Gaussian process regression to return a final estimate of mortality along with uncertainty. ST-GPR estimates comprised final deaths estimates for Group 2A.

Section 2.5.6. Additional Adjustments

Additional adjustments enabled us to use case surveillance data and HIV mortality estimated as part of the GBD all-cause mortality life table process. In countries and territories with high-quality case notification data, we scaled incidence results to align with case reports after accounting for an assumed average of five years' lag to diagnosis. For Group 1 countries and territories, we used an ensemble approach to reconcile the differences between HIV mortality estimated by EPP-ASM and by HIV mortality estimated as a part of the GBD all-cause mortality life table process to generate final HIV mortality. For ages 15 and over, the ensemble model averaged HIV mortality estimates from the two processes with equal weights. For under age 15, we applied the fraction of deaths due to HIV in Spectrum to estimate all-cause mortality to generate HIV-specific mortality and mortality from all other causes (HIV-free mortality).

Since EPP-ASM produces HIV incidence, prevalence, and deaths that are consistent with one another over time, the reckoning process results in death numbers that are no longer consistent with the incidence and prevalence produced in Spectrum. In order to recreate this consistency, we recalculated incidence for all Group 1 locations using reckoned deaths and prevalence produced by EPP-ASM. The updated incidence is calculated by aggregating counts of new infections, HIV deaths from EPP-ASM, and HIV deaths after reckoning at the year-sex level. The difference between reckoned HIV deaths and HIV deaths

from EPP-ASM is added to EPP-ASM incidence, and we calculate the ratio between updated incidence and EPP-ASM incidence. Age–specific counts of new infections are then scaled by their corresponding sex–year ratios.

For countries with high quality case reports data we then took an additional step of scaling Spectrum incidence to the case reports. We assumed a five-year lag to diagnosis, meaning, for example, that case reports from 2008 were assumed to be incident cases in year 2003. We applied the scalar from the first year of case reports data to years prior to case reports data. For years after the five-year lag on the most recent case reports data, we applied the same scalar from the last year with case reports data, resulting in an adjustment on the full incidence time series. Importantly, we only scaled upwards. In years where the case reports reported lower incident cases than the Spectrum estimates, we did not scale the incidence.

Section 2.6. References

1 Vos T, Lim SS, Abbafati C, *et al.* Global burden of 369 diseases and injuries in 204 countries and territories, 1990–2019: a systematic analysis for the Global Burden of Disease Study 2019. *The Lancet* 2020; **396**: 1204–22.

2 Frank TD, Carter A, Jahagirdar D, *et al.* Global, regional, and national incidence, prevalence, and mortality of HIV, 1980–2017, and forecasts to 2030, for 195 countries and territories: a systematic analysis for the Global Burden of Diseases, Injuries, and Risk Factors Study 2017. *The Lancet HIV* 2019; **6**: e831–59.

3 Ghys PD, Williams BG, Over M, Hallett TB, Godfrey-Faussett P. Epidemiological metrics and benchmarks for a transition in the HIV epidemic. *PLOS Medicine* 2018; **15**: e1002678.

4 Naghavi M, Abajobir AA, Abbafati C, *et al.* Global, regional, and national age-sex specific mortality for 264 causes of death, 1980–2016: a systematic analysis for the Global Burden of Disease Study 2016. *The Lancet* 2017; **390**: 1151–210.

5 Eaton JW, Brown T, Puckett R, *et al.* The Estimation and Projection Package Age-Sex Model and the r-hybrid model: new tools for estimating HIV incidence trends in sub-Saharan Africa. *AIDS* 2019; **33**: S235.

6 Saito S, Chung H, Mahy M, *et al.* Pediatric HIV Treatment Gaps in 7 East and Southern African Countries: Examination of Modeled, Survey, and Routine Program Data. *J Acquir Immune Defic Syndr* 2018; **78 Suppl 2**: S134–41.

7 CDC. Pneumocystis Pneumonia --- Los Angeles. MMWR Wkly. 1981; published online June 5. http://www.cdc.gov/mmwr/preview/mmwrhtml/june_5.htm (accessed February 4, 2021).

Section 2.7. Figures and tables

Figure S4A. Deaths due to HIV per 1000 in 2019

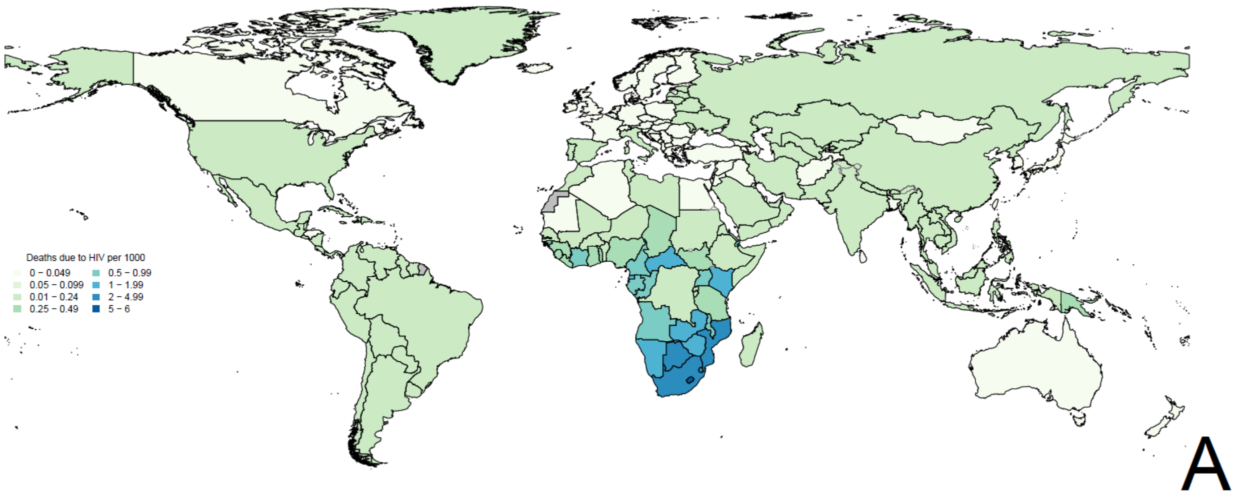

Figure S4B. Incident cases of HIV per 1000 in 2019

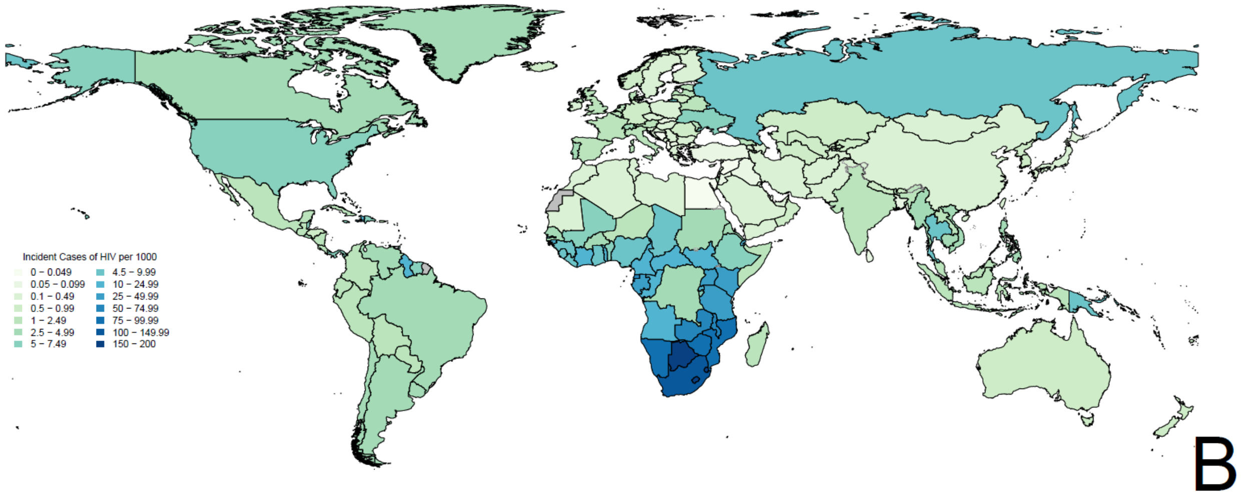

Figure S4C. Prevalent cases of HIV per 1000 in 2019

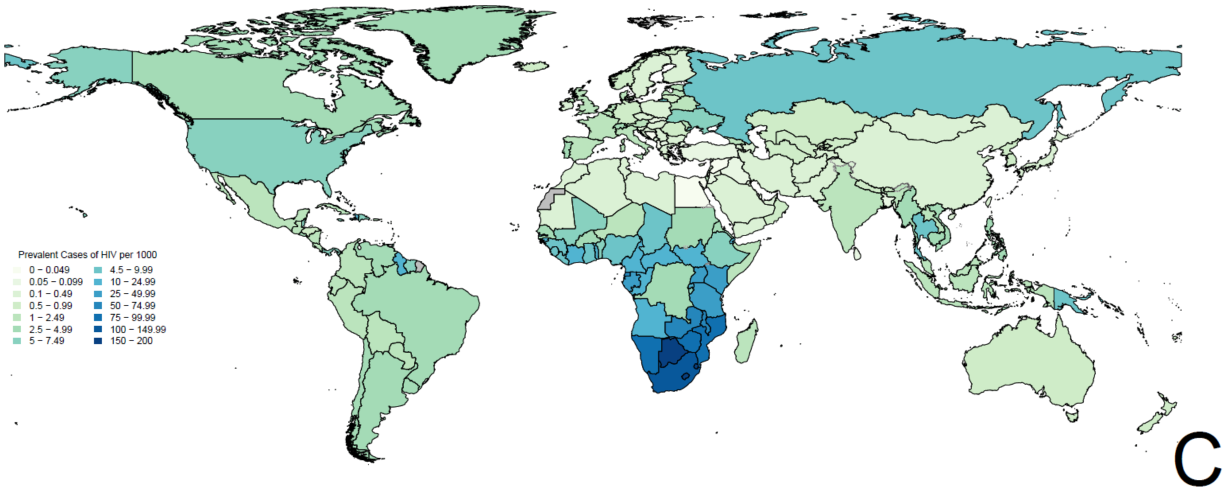

C

Figure S5. Percentage share of global burden of HIV incident cases (A), deaths (B) and prevalent cases (C) in each GBD super-region, by sex, 2019. Dots represent the percentage share of the global population in each super-region. GBD=Global Burden of Diseases, Injuries, and Risk Factors Study.

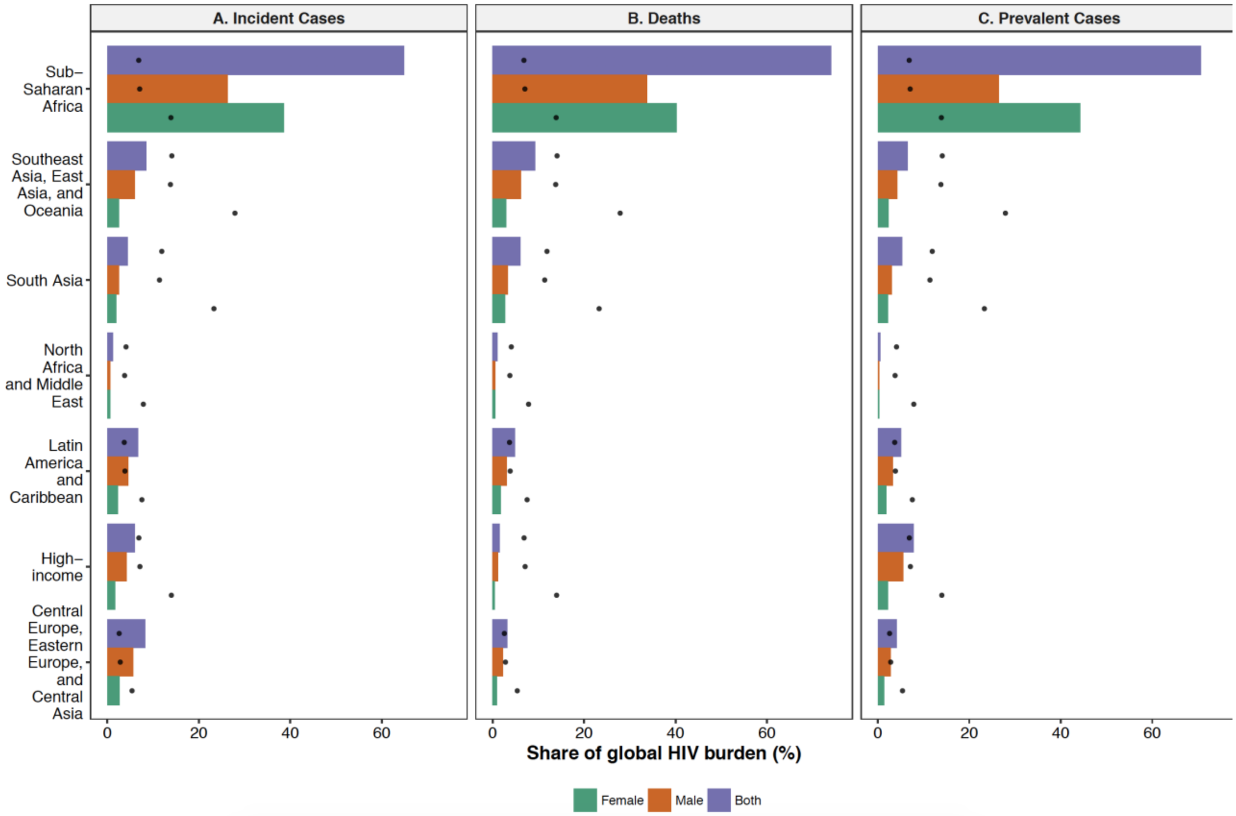

Figure S6. Deaths due to HIV (in thousands) from 1990 to 2019 by super region

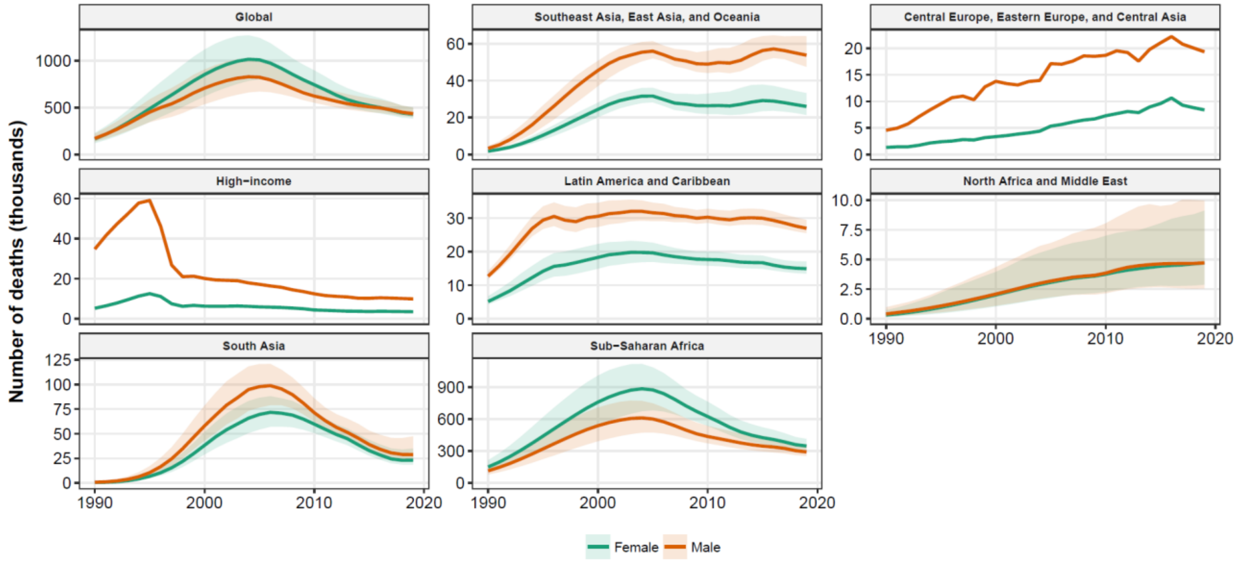

Figure S7. Incident cases of HIV (in thousands) from 1990 to 2019 by super region

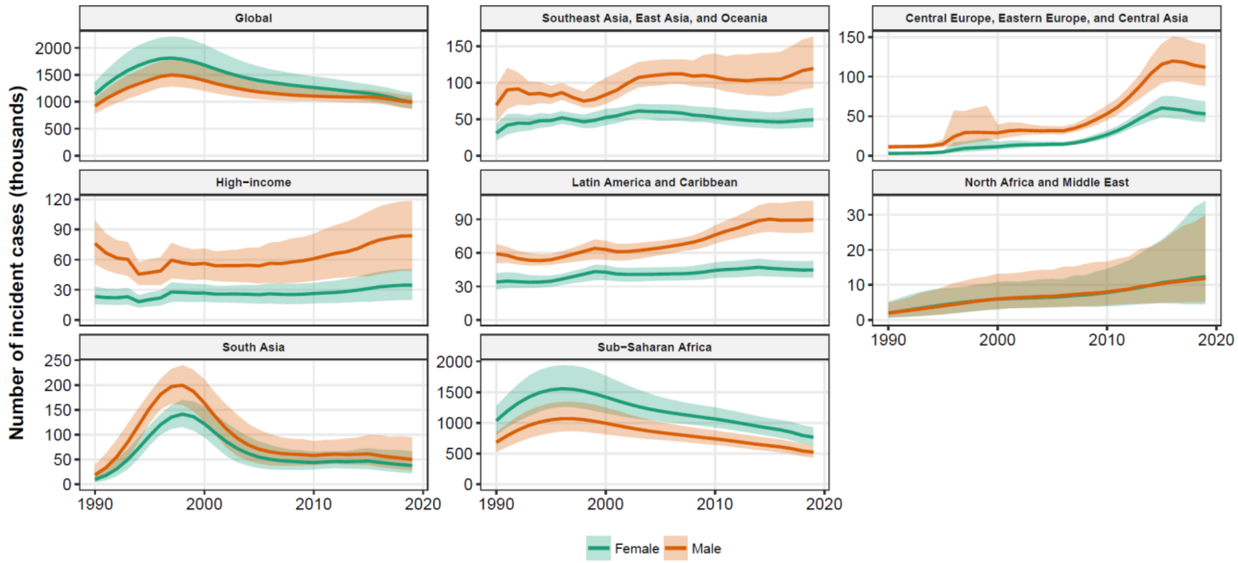

Figure S8. Prevalent cases of HIV (in thousands) from 1990 to 2019 by super region

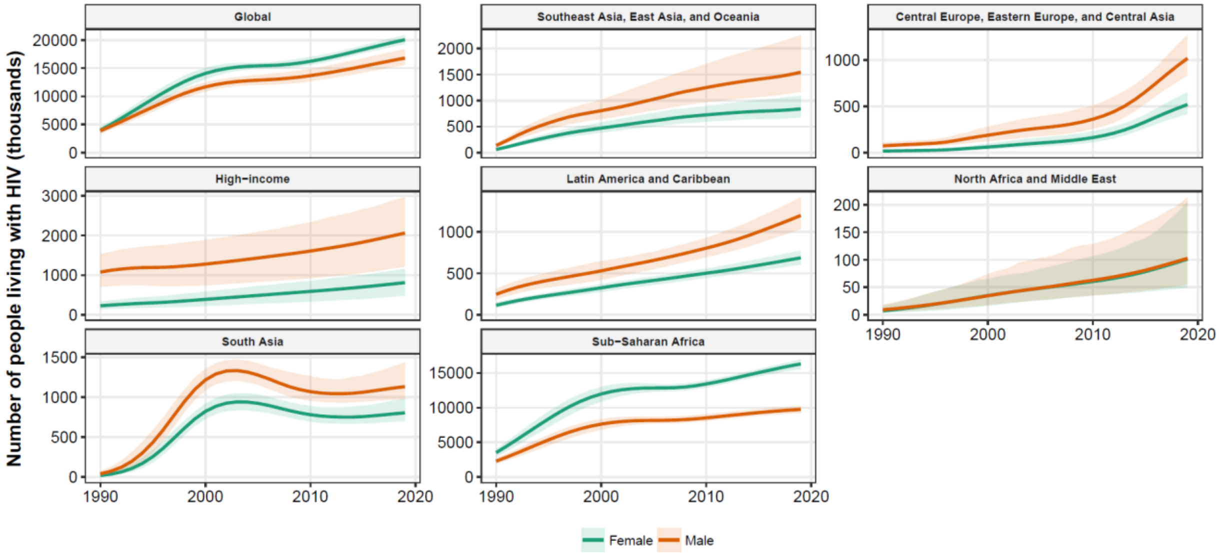

Table S1. Number of deaths due to HIV and percent change between 2010 and 2019 for GBD super-regions and 204 countries and territories

**Location**	**2010 (95% UI)**	**2019 (95% UI)**	**Percent Change (%)**
**Global**	**1365783.06 (1200970.96 – 1627979.02)**	**863837.35 (786074.86 – 996044.87)**	**-36.75 (-39.95 – -32.64)**
**Central Europe, eastern Europe, and central Asia**	**25938.72 (25692.93 – 26208.49)**	**27715.47 (27352.19 – 28093.88)**	**6.85 (5.1 – 8.57)**
Armenia	10.45 (9.85 – 11.08)	19.08 (18.11 – 20.13)	82.47 (68.05 – 96.27)
Azerbaijan	42.07 (32.4 – 52.75)	37.11 (29.86 – 42.38)	-11.79 (-36.92 – 23.53)
Georgia	8.48 (8.1 – 8.92)	23.28 (22.19 – 24.5)	174.52 (156.54 – 195.18)
Kazakhstan	311 (299.48 – 322.57)	260.54 (246.37 – 276)	-16.22 (-21.58 – -10.22)
Kyrgyzstan	124.59 (120.17 – 128.93)	170.04 (162.07 – 177.8)	36.48 (28.76 – 45.01)
Mongolia	8.12 (3.7 – 14.86)	15.03 (1.78 – 38.02)	85.09 (-54.71 – 210.81)

Tajikistan	119.89<br>(107.1 – 133.45)	60.02<br>(45.02 – 87.39)	-49.93<br>(-64.42 – -21.92)
Turkmenistan	146.57<br>(140.36 – 153.1)	105.37<br>(99.05 – 112.62)	-28.11<br>(-33.31 – -22.26)
Uzbekistan	544.15<br>(526.01 – 561.84)	584.56<br>(553.98 – 617.1)	7.43<br>(0.85 – 14.27)
Albania	1.96<br>(1.86 – 2.07)	1.65<br>(1.47 – 1.9)	-16.13<br>(-23.72 – -6.8)
Bosnia and Herzegovina	1.87<br>(1.46 – 2.56)	1.71<br>(0.93 – 3.65)	-8.58<br>(-45.39 – 65.62)
Bulgaria	70.46<br>(67.41 – 73.81)	45.91<br>(43.26 – 48.94)	-34.84<br>(-39.29 – -29.74)
Croatia	12.15<br>(11.53 – 12.78)	7.51<br>(7.14 – 7.91)	-38.19<br>(-42.58 – -33.43)
Czech Republic	15.78<br>(14.97 – 16.61)	18.86<br>(17.85 – 19.88)	19.53<br>(10.74 – 28.66)
Hungary	67.37<br>(64.02 – 70.94)	37.25<br>(34.89 – 39.73)	-44.7<br>(-49.15 – -40.07)
Montenegro	1.74<br>(1.47 – 2.09)	1.35<br>(1.18 – 1.59)	-22.28<br>(-36.14 – -9.12)
North Macedonia	2.11<br>(1.77 – 2.52)	1.91<br>(1.6 – 2.19)	-9.57<br>(-19.95 – 1.24)
Poland	161.07<br>(158.39 – 163.92)	134.09<br>(131.53 – 136.75)	-16.75<br>(-18.85 – -14.57)
Romania	107.31<br>(103.53 – 111.27)	142.17<br>(135.79 – 149.26)	32.49<br>(24.75 – 40.79)
Serbia	76.17<br>(40.05 – 169.87)	47.86<br>(29.1 – 103.37)	-37.16<br>(-68.33 – 28.92)
Slovakia	4.17<br>(3.09 – 5.43)	4.14<br>(3.44 – 4.94)	-0.76<br>(-20.76 – 21.79)
Slovenia	1.23<br>(1.16 – 1.3)	2.16<br>(2.03 – 2.3)	76.22<br>(62.33 – 92.36)
Belarus	378.93<br>(365.5 – 392.36)	293.71<br>(276.68 – 311.86)	-22.49<br>(-27.78 – -17.02)
Estonia	43.04<br>(41 – 45.3)	34.03<br>(31.9 – 36.18)	-20.92<br>(-27.24 – -14.45)

Latvia	131.39<br>(126.61 – 136.1)	117.88<br>(112.22 – 123.5)	-10.28<br>(-15.39 – -5.16)
Lithuania	89.18<br>(85.21 – 93.45)	60.74<br>(58.08 – 63.62)	-31.89<br>(-36.08 – -27.28)
Moldova	211.15<br>(203.58 – 219.16)	162.86<br>(152.58 – 172.85)	-22.87<br>(-28.36 – -17.11)
Russia	14256.48<br>(14175.23 – 14339.43)	18681.54<br>(18480.09 – 18876.36)	31.04<br>(29.51 – 32.65)
Ukraine	8989.85<br>(8762.46 – 9219.12)	6643.09<br>(6353.09 – 6948.76)	-26.1<br>(-30 – -22.01)
**High income**	**16796.28<br>(16716.96 – 16870.01)**	**13392.55<br>(13283.45 – 13502.11)**	**-20.26<br>(-21.02 – -19.55)**
Australia	82.57<br>(80.13 – 84.9)	70.65<br>(68.43 – 72.86)	-14.43<br>(-18.02 – -10.57)
New Zealand	15.16<br>(14.68 – 15.64)	8.7<br>(8.47 – 8.94)	-42.63<br>(-45.02 – -40.02)
Brunei	5.01<br>(4.67 – 5.34)	5.29<br>(4.63 – 6.01)	5.54<br>(-8.21 – 21.48)
Japan	214.63<br>(211.16 – 218.13)	160.69<br>(157.64 – 163.6)	-25.13<br>(-27 – -23.25)
South Korea	149.36<br>(142.86 – 156.05)	138.19<br>(128.53 – 148.87)	-7.48<br>(-15.26 – 0.14)
Singapore	38.09<br>(35.53 – 40.58)	35.99<br>(33.46 – 38.77)	-5.52<br>(-14.52 – 4.99)
Canada	329.62<br>(319.03 – 340.49)	253.56<br>(242.05 – 266.13)	-23.08<br>(-27.55 – -18.7)
Greenland	2.71<br>(2.34 – 3.02)	1.62<br>(1.29 – 1.97)	-40.24<br>(-51.71 – -29.15)
USA	9143.13<br>(9085.23 – 9205.03)	7052.74<br>(6970.28 – 7132.64)	-22.86<br>(-23.96 – -21.88)
Georgia	8.48<br>(8.1 – 8.92)	23.28<br>(22.19 – 24.5)	174.52<br>(156.54 – 195.18)
Argentina	1650.81<br>(1625.07 – 1677.73)	1776.98<br>(1737.57 – 1815.01)	7.64<br>(4.66 – 10.6)
Chile	451.11<br>(443.16 – 459.2)	492.01<br>(480.54 – 503.89)	9.07<br>(5.75 – 12.49)

Uruguay	195.63 (191.86 – 199.72)	177.38 (173.61 – 181.36)	-9.33 (-12.01 – -6.77)
Andorra	3.33 (0.25 – 13.55)	3.1 (0.21 – 14.26)	-6.89 (-51.31 – 57.21)
Austria	45.49 (43.59 – 47.55)	35.47 (33.73 – 37.25)	-22.04 (-27.08 – -17.02)
Belgium	82.51 (79.6 – 85.75)	55.97 (53.46 – 58.53)	-32.17 (-36.01 – -28.08)
Cyprus	3.72 (3.57 – 3.88)	4.63 (4.22 – 5.08)	24.52 (14.61 – 35.18)
Denmark	41.29 (39.51 – 43.04)	24.41 (23.31 – 25.54)	-40.87 (-44.43 – -36.89)
Finland	11.36 (10.84 – 11.98)	5.45 (5.22 – 5.7)	-51.99 (-55.06 – -48.75)
France	584.22 (569.03 – 601.83)	456.98 (434.35 – 480.32)	-21.78 (-26.16 – -17.24)
Germany	551.85 (536.96 – 566.91)	421.31 (402.16 – 442.48)	-23.65 (-27.62 – -19.34)
Greece	19.73 (18.63 – 20.79)	22.48 (21.28 – 23.68)	13.94 (6 – 22.88)
Iceland	1.15 (1.09 – 1.2)	0.88 (0.84 – 0.93)	-22.82 (-28.01 – -17.16)
Ireland	12.98 (12.38 – 13.63)	8.63 (8.19 – 9.08)	-33.53 (-38.08 – -28.7)
Israel	31.39 (30.19 – 32.61)	36.69 (35.15 – 38.29)	16.89 (10.34 – 23.47)
Italy	834.97 (823.18 – 847.5)	620.89 (606.95 – 634.56)	-25.64 (-27.62 – -23.5)
Luxembourg	2.54 (2.42 – 2.66)	2.49 (2.37 – 2.61)	-2.02 (-8.86 – 5.49)
Malta	1.77 (1.68 – 1.85)	1.41 (1.33 – 1.48)	-20.42 (-25.58 – -14.48)
Monaco	0.85 (0.26 – 2.36)	0.85 (0.21 – 2.58)	-0.12 (-40.05 – 42.15)
Netherlands	58.74 (56.45 – 61.26)	48.63 (46.44 – 50.99)	-17.2 (-22 – -11.75)

Norway	16.11 (15.73 – 16.47)	13.69 (13.41 – 13.99)	-14.99 (-17.44 – -12.21)
Portugal	732.36 (712.26 – 754.32)	482.07 (457.19 – 508.78)	-34.18 (-37.92 – -29.9)
San Marino	0.65 (0.19 – 1.77)	0.67 (0.16 – 2.08)	2.5 (-37.38 – 48.06)
Spain	1077.77 (1046.96 – 1109.63)	678.89 (640.2 – 717.56)	-37.01 (-40.91 – -33.1)
Sweden	23.14 (22.48 – 23.86)	20.41 (19.83 – 21.03)	-11.79 (-15.55 – -8.1)
Switzerland	48.1 (46.25 – 50.15)	36.89 (35.02 – 38.67)	-23.3 (-28.11 – -18.37)
UK	328.24 (326.09 – 330.31)	232.79 (231.26 – 234.28)	-29.08 (-29.74 – -28.39)
**Latin America and Caribbean**	**47910.51 (44674.3 – 51985.53)**	**41867.46 (39435.9 – 46127.73)**	**-12.61 (-18.36 – -6.92)**
Bolivia	733.65 (67.31 – 2711.3)	732.08 (40.53 – 4632.63)	-0.21 (-79.22 – 81.59)
Ecuador	1050.8 (1017.88 – 1083.09)	1207.31 (1128.86 – 1291.29)	14.9 (6.84 – 24.56)
Peru	2051.93 (1699.83 – 2523.75)	2401.05 (1393.51 – 3905.46)	17.01 (-21.37 – 61.79)
Antigua and Barbuda	8.1 (7.94 – 8.27)	7.36 (7.23 – 7.51)	-9.12 (-11.61 – -6.53)
The Bahamas	125.58 (123.32 – 128.11)	116.15 (113.85 – 118.48)	-7.51 (-10.06 – -4.91)
Barbados	31.41 (30.77 – 32.04)	24.76 (24.29 – 25.22)	-21.16 (-23.37 – -18.95)
Belize	76.17 (74.62 – 77.65)	80.03 (78.43 – 81.76)	5.07 (2.09 – 8.35)
Bermuda	7.3 (7.16 – 7.45)	6.25 (6.13 – 6.38)	-14.39 (-16.82 – -11.73)
Cuba	227.87 (223.03 – 232.82)	337.47 (329.71 – 345.73)	48.1 (43.37 – 52.75)
Dominica	5.75 (5.62 – 5.89)	5.01 (4.91 – 5.12)	-12.81 (-15.29 – -10.09)
Dominican Republic	2169.3 (1768.33 – 2798.24)	1362.08 (982.51 – 2015.46)	-37.21 (-52.65 – -17.59)

Grenada	6.59 (6.46 – 6.72)	5.46 (5.34 – 5.57)	-17.24 (-19.67 – -14.7)
Guyana	204.42 (200.84 – 208.01)	180.82 (177.53 – 184.07)	-11.55 (-13.85 – -9.31)
Haiti	10370.03 (8211.72 – 13163.69)	5908.15 (4655.48 – 7676.8)	-43.03 (-53.11 – -32.27)
Jamaica	617.6 (606.45 – 628.68)	418 (410.6 – 425.41)	-32.32 (-34.04 – -30.46)
Puerto Rico	372.3 (365.85 – 378.71)	231.43 (226.99 – 236.06)	-37.84 (-39.45 – -36.18)
Saint Kitts and Nevis	20.21 (4.57 – 46.68)	31.78 (5.34 – 83.98)	57.24 (-33.24 – 138.63)
Saint Lucia	7.99 (7.83 – 8.15)	7.24 (7.1 – 7.38)	-9.36 (-11.76 – -6.74)
Saint Vincent and the Grenadines	22.32 (21.87 – 22.8)	19.96 (19.54 – 20.35)	-10.61 (-12.99 – -7.92)
Suriname	128.13 (125.68 – 130.6)	100.43 (98.66 – 102.17)	-21.62 (-23.58 – -19.64)
Trinidad and Tobago	229.05 (224.88 – 233.26)	206.42 (202.23 – 210.3)	-9.88 (-12.32 – -7.26)
Virgin Islands	10.03 (9.84 – 10.24)	7.86 (7.7 – 8)	-21.69 (-23.9 – -19.46)
Colombia	2740.66 (2677.78 – 2801.38)	2449.69 (2334.69 – 2565.8)	-10.62 (-15.27 – -5.74)
Costa Rica	156.12 (150.85 – 161.71)	145.61 (138.67 – 152.31)	-6.73 (-12.5 – -1.02)
El Salvador	765.62 (499.85 – 1052.57)	680.25 (362.18 – 975.67)	-11.15 (-31.75 – 12.13)
Guatemala	707.7 (691.52 – 723.21)	668.77 (638.27 – 701.12)	-5.5 (-10.21 – -0.42)
Honduras	79.04 (63.91 – 104.87)	91.39 (68.24 – 121.53)	15.63 (-12.11 – 40.19)
Mexico	5694.39 (5649.6 – 5739.45)	5034.32 (4976.08 – 5091.25)	-11.59 (-12.79 – -10.38)
Nicaragua	274.51 (187.98 – 342.88)	561.15 (337.88 – 804.43)	104.42 (55.39 – 166.7)
Panama	567.64 (553.14 – 582.29)	540.43 (515.95 – 566.01)	-4.79 (-9.69 – 0.55)

Venezuela	1941.35<br>(1896.29 – 1989.12)	1850.07<br>(1763.03 – 1946.28)	-4.7<br>(-9.6 – 0.56)
Brazil	15501.66<br>(15363.3 – 15638.97)	15560.83<br>(15297 – 15837.29)	0.38<br>(-1.54 – 2.36)
Paraguay	488.98<br>(311.97 – 614.46)	570.3<br>(342.89 – 817.36)	16.63<br>(-8.99 – 46.68)
**North Africa and Middle East**	**7603.91<br>(4542.2 – 14139.93)**	**9433.34<br>(5505.64 – 18554.68)**	**24.06<br>(-14.03 – 81.8)**
Afghanistan	176.47<br>(12.04 – 694.23)	317.68<br>(5.31 – 1503.16)	80.02<br>(-76.54 – 275.11)
Algeria	329.47<br>(19.36 – 2025.1)	263.55<br>(17.6 – 1943.65)	-20.01<br>(-71.3 – 47.04)
Bahrain	12.45<br>(11.61 – 13.19)	7.36<br>(5.7 – 10.23)	-40.91<br>(-53.32 – -16.7)
Egypt	95.26<br>(82.3 – 107.81)	55.92<br>(45.19 – 72.38)	-41.3<br>(-49.53 – -29.43)
Iran	565.58<br>(486.14 – 646)	1176.65<br>(851.73 – 1714.5)	108.04<br>(52.23 – 189.15)
Iraq	68.23<br>(25.93 – 218.86)	82.18<br>(16.47 – 317.55)	20.44<br>(-39.08 – 58.68)
Jordan	15.9<br>(13.7 – 18.53)	27.35<br>(20.32 – 36.35)	71.97<br>(34.57 – 111.58)
Kuwait	3.22<br>(2.85 – 3.6)	2.87<br>(2.52 – 3.27)	-10.61<br>(-25.33 – 5.41)
Lebanon	60.14<br>(1.5 – 298.22)	61.11<br>(1.52 – 283.03)	1.61<br>(-62.55 – 146.49)
Libya	64.72<br>(1.57 – 331.3)	84.89<br>(1.43 – 471.03)	31.16<br>(-73.22 – 170.06)
Morocco	896.75<br>(14.67 – 4186.38)	494.28<br>(12.97 – 3033.72)	-44.88<br>(-79.57 – 6.16)
Oman	71.26<br>(37.81 – 107.97)	98.45<br>(55.48 – 151.31)	38.15<br>(-16.53 – 70.85)
Palestine	11.16<br>(10.34 – 11.97)	13.62<br>(11.63 – 15.99)	22.01<br>(10.29 – 36.75)
Qatar	4.29<br>(3.41 – 4.97)	4.29<br>(3.59 – 5.03)	-0.1<br>(-12.99 – 17.58)
Saudi Arabia	509.73<br>(325.36 – 1177.99)	683.18<br>(276.88 – 1810.87)	34.03<br>(-21.51 – 68.9)

Sudan	4110.25 (2284.94 – 7246.33)	5154.88 (2590.53 – 10324.19)	25.42 (-21.41 – 123.93)
Syria	23.04 (19.93 – 27.93)	14.46 (11.36 – 19.42)	-37.22 (-45.93 – -19.76)
Tunisia	109.66 (5.54 – 599.18)	147.79 (6.62 – 811.61)	34.78 (-50.21 – 104.74)
Turkey	112.02 (91.11 – 139.51)	226.22 (181.62 – 264.93)	101.95 (60.34 – 144.22)
United Arab Emirates	96 (2.71 – 467.65)	160.33 (2.91 – 928.84)	67.02 (-60.19 – 284.28)
Yemen	260.79 (7.05 – 1112.99)	346.7 (6.55 – 1776.31)	32.95 (-71.55 – 224.8)
**South Asia**	**130994.15 (113428.54 – 156655.86)**	**52070.06 (42176.16 – 82535.99)**	**-60.25 (-66.18 – -44.03)**
Bangladesh	246.34 (4.1 – 1305.84)	358.51 (3.61 – 2262.55)	45.53 (-64.15 – 195.48)
Bhutan	26.11 (0.55 – 124.73)	30.65 (0.54 – 207.13)	17.38 (-74.75 – 160.26)
India	127158.94 (112381.78 – 147091.52)	46297.75 (40986.6 – 53461.46)	-63.59 (-66.85 – -58.24)
Nepal	2318.33 (37.39 – 11532.03)	1433.8 (24.41 – 8641.66)	-38.15 (-80.23 – 12.58)
Pakistan	1244.43 (28.41 – 5574.51)	3949.35 (43.61 – 22919.25)	217.36 (-47.7 – 602.91)
**Southeast Asia, east Asia, and Oceania**	**75314.17 (67311.89 – 88092.88)**	**79803.96 (69355.78 – 93026.29)**	**5.96 (-8.69 – 18.17)**
China	16722.12 (15362.42 – 18480.49)	31746.08 (25775.17 – 37448.33)	89.84 (39.8 – 138.08)
North Korea	361.2 (9.44 – 2131.1)	760.96 (15.88 – 4064.56)	110.68 (-2.01 – 396.96)
Taiwan (province of China)	155.99 (148.87 – 163.95)	171.08 (156.95 – 186.27)	9.67 (-0.63 – 20.91)
American Samoa	0.57 (0.2 – 1.21)	0.48 (0.09 – 1.29)	-15.04 (-61.12 – 20.55)

Cook Islands	0.75<br>(0.03 – 3.58)	1.71<br>(0.02 – 8.12)	127.18<br>(-31.43 – 301.69)
Fiji	11.96<br>(9.24 – 15.94)	8.81<br>(5.99 – 13.75)	-26.38<br>(-38.72 – -10.87)
Guam	6.28<br>(3.51 – 11.34)	7.01<br>(2.96 – 15.01)	11.74<br>(-21.84 – 49.17)
Kiribati	1.7<br>(1.45 – 1.97)	1.6<br>(1.21 – 2.1)	-6.06<br>(-23.5 – 15.74)
Marshall Islands	2.16<br>(0.02 – 14.63)	3.95<br>(0.02 – 31.88)	82.45<br>(-60.99 – 536.48)
Federated States of Micronesia	16.7<br>(1.62 – 90.17)	45.36<br>(1.13 – 228.69)	171.58<br>(-35.96 – 537.79)
Nauru	0.38<br>(0.01 – 1.97)	0.82<br>(0.01 – 3.99)	115.94<br>(-33.71 – 287.91)
Niue	0.06<br>(0 – 0.3)	0.15<br>(0 – 0.73)	135.22<br>(-31.96 – 316.66)
Northern Mariana Islands	0.9<br>(0.38 – 1.83)	0.59<br>(0.09 – 1.4)	-34.41<br>(-76.82 – -10.06)
Palau	0.78<br>(0.03 – 3.94)	1.86<br>(0.03 – 8.98)	137.34<br>(-24.09 – 317.36)
Papua New Guinea	3520.94<br>(1089.1 – 8560.62)	3829.61<br>(1169.65 – 10720.02)	8.77<br>(-19.33 – 32.23)
Samoa	6.99<br>(0.06 – 47.65)	13.46<br>(0.07 – 107.02)	92.52<br>(-57.81 – 657.29)
Solomon Islands	22.69<br>(0.19 – 151.4)	40.57<br>(0.21 – 325.28)	78.77<br>(-60.96 – 512.24)
Tokelau	0.05<br>(0 – 0.23)	0.12<br>(0 – 0.57)	151.08<br>(-21.59 – 349.31)
Tonga	1.64<br>(0.67 – 3.57)	1.84<br>(0.36 – 5.02)	11.88<br>(-52.11 – 55.76)
Tuvalu	0.39<br>(0.01 – 1.93)	0.98<br>(0.02 – 4.76)	148.44<br>(-27.96 – 337.5)

Vanuatu	10.48<br>(0.08 – 73.43)	19.03<br>(0.1 – 156.14)	81.62<br>(-59.54 – 584.57)
Cambodia	3361.33<br>(2309.67 – 4544.54)	1415.55<br>(940.7 – 2006.02)	-57.89<br>(-69.54 – -42.2)
Indonesia	3498.75<br>(3082.71 – 4148.15)	5602.85<br>(4492.25 – 7429.45)	60.14<br>(38.5 – 87.55)
Laos	389.25<br>(2.5 – 2529.6)	373.86<br>(2.9 – 2708.03)	-3.95<br>(-71.69 – 130.86)
Malaysia	2144.79<br>(1787.63 – 2536.13)	1520.83<br>(997.26 – 2096.22)	-29.09<br>(-48.04 – -5.63)
Maldives	0.57<br>(0.52 – 0.63)	0.86<br>(0.78 – 0.95)	50.91<br>(31.18 – 75.34)
Mauritius	72.07<br>(67.92 – 76.12)	91.38<br>(85.14 – 98.01)	26.79<br>(15.25 – 39.85)
Myanmar	16109.22<br>(13343.1 – 19759.49)	4772.85<br>(3813.23 – 6048.05)	-70.37<br>(-75.41 – -62.82)
Philippines	5191.94<br>(5118.23 – 5271.71)	5222.48<br>(5136.05 – 5314.86)	0.59<br>(-1.43 – 2.57)
Seychelles	4.62<br>(4.24 – 5.18)	4.02<br>(3.09 – 5.18)	-13.02<br>(-34.87 – 5.6)
Sri Lanka	67.45<br>(58.87 – 77.13)	37.99<br>(26.78 – 58.32)	-43.68<br>(-59.06 – -17.42)
Thailand	16260.64<br>(12564.94 – 22508.32)	17214.73<br>(13128.19 – 25526.44)	5.87<br>(-17.7 – 23.66)
Timor-Leste	251.72<br>(2.89 – 1426.19)	237.58<br>(2.33 – 1420.3)	-5.62<br>(-71.15 – 177.88)
Vietnam	6847.86<br>(6039.65 – 7846.34)	6399.44<br>(4916.18 – 8629.51)	-6.55<br>(-18.75 – 13.6)
**Sub–Saharan Africa**	**1061225.31<br>(912572.02 – 1293232)**	**639554.52<br>(561276.56 – 766064.65)**	**-39.73<br>(-42.36 – -36.49)**
Angola	12035.67<br>(8368.24 – 17229.82)	16801.83<br>(11489.49 – 24344.12)	39.6<br>(17.79 – 68.65)
Central African Republic	9369.11<br>(7515.37 – 11574.82)	5546.61<br>(4439.29 – 7431.36)	-40.8<br>(-50.9 – -29.16)
Congo (Brazzaville)	5965.85<br>(4833.92 – 7458.47)	4274.98<br>(3509.99 – 5284.39)	-28.34<br>(-36.11 – -20.05)

DR Congo	34894.23 (27443.77 – 44311.27)	10237.54 (7790.54 – 13782.62)	-70.66 (-75.65 – -62.18)
Equatorial Guinea	1580.31 (1105.53 – 2212.13)	2117.65 (1318.14 – 3471.89)	34 (-6.25 – 94.79)
Gabon	1907.55 (1323.02 – 2694.82)	1115.21 (786.16 – 1600.26)	-41.54 (-58.41 – -18.48)
Burundi	7519.77 (6380.51 – 8980.98)	2047.56 (1681.88 – 2546.64)	-72.77 (-77.5 – -65.43)
Comoros	1.22 (0.01 – 9.01)	1.86 (0.02 – 13.85)	52.05 (-52.34 – 478.63)
Djibouti	1099.1 (826.97 – 1444.03)	903.02 (647.69 – 1296.4)	-17.84 (-32.6 – 0.13)
Eritrea	2413.9 (1738.31 – 3229.95)	1378.25 (982.51 – 1892.77)	-42.9 (-51.82 – -29.76)
Ethiopia	47037.51 (38815.71 – 57784.89)	26590.89 (21751.56 – 33029.4)	-43.47 (-49.35 – -37.13)
Kenya	80975.31 (72307.94 – 94361.47)	51134.61 (45036.35 – 58977.47)	-36.85 (-43.09 – -30.81)
Madagascar	1864.87 (1229.69 – 2748.81)	2529.73 (1645.6 – 3727.56)	35.65 (12.39 – 67.17)
Malawi	39192.49 (33792.79 – 48167.14)	14226.62 (12204.2 – 17576.51)	-63.7 (-67.51 – -59.65)
Mozambique	74461.39 (60090.05 – 94767.08)	66304.05 (53843.37 – 86579.82)	-10.96 (-23.86 – 4.18)
Rwanda	5745.1 (4752.82 – 7303.78)	3065.06 (2606.6 – 3735.32)	-46.65 (-55.43 – -38.7)
Somalia	4072.81 (2880.07 – 5597.48)	3188.21 (2332.2 – 4335)	-21.72 (-32.41 – -8.13)
South Sudan	6302.96 (3295.96 – 10999.73)	4390.88 (1896.72 – 8709.59)	-30.34 (-56.63 – 13.87)
Uganda	61949.03 (51568.04 – 75813.18)	20762.27 (16892.62 – 26840.11)	-66.48 (-70.92 – -61.16)
Tanzania	80829.61 (68397.33 – 98572.87)	27125.17 (22630.13 – 33798.61)	-66.44 (-70.64 – -61.31)

Zambia	31937.17 (27141.13 – 40314.12)	22540.27 (19287.01 – 27224.58)	-29.42 (-39.64 – -19.61)
Botswana	7330.33 (6157.22 – 9571.41)	5612.49 (4639.66 – 7197.49)	-23.43 (-35.58 – -10.06)
eSwatini	7345.54 (6016.06 – 9392.76)	3321.36 (2892.32 – 3991.05)	-54.78 (-62.27 – -45.95)
Lesotho	11834.06 (9894.29 – 15178.94)	10792.65 (9013.96 – 13646.02)	-8.8 (-15.19 – -1.87)
Namibia	5175.82 (4330.87 – 6605.68)	3772.47 (3273.18 – 4434.59)	-27.11 (-36.93 – -19.81)
South Africa	240023.99 (207318.11 – 298800.64)	143850.63 (123860.99 – 177382.31)	-40.07 (-44.01 – -35.49)
Zimbabwe	57069.34 (50491.31 – 68188.89)	20722.24 (18852.9 – 23072.47)	-63.69 (-68.04 – -59.55)
Benin	2538.02 (1978.4 – 3328.43)	2033.47 (1494.34 – 2748.98)	-19.88 (-33.28 – -6.16)
Burkina Faso	6384.18 (5308.88 – 7816.66)	2958.91 (2388.71 – 3703.64)	-53.65 (-58.84 – -48.1)
Cape Verde	106.92 (50.54 – 184.78)	54.57 (27.62 – 123.01)	-48.96 (-66.91 – -19.08)
Cameroon	34776.96 (27726.13 – 43597.65)	23172.25 (19920.18 – 28094.46)	-33.37 (-39.66 – -24.8)
Chad	4460.89 (2976.32 – 6493.63)	4695.32 (3225.4 – 6822.24)	5.26 (-14.13 – 31.09)
The Gambia	1116.57 (729.87 – 1645.53)	1078.27 (708.18 – 1576.95)	-3.43 (-18.58 – 18.47)
Ghana	20587.25 (17029.4 – 25374.43)	14620.93 (11988.57 – 18358.14)	-28.98 (-33.91 – -23.7)
Guinea	4332.66 (3188.06 – 5964.72)	4037.04 (2810.43 – 5857.31)	-6.82 (-23.24 – 16.82)
Guinea–Bissau	1462.63 (912.02 – 2208.24)	1002.61 (586.88 – 1647.47)	-31.45 (-43.24 – -15.63)
Liberia	2256.58 (1643.64 – 3024.88)	1651.95 (1277.25 – 2203.36)	-26.79 (-41.67 – -6.77)
Mali	5077.16 (3891.85 – 6535.59)	4495.76 (3328.85 – 5870.16)	-11.45 (-28.54 – 7.46)

Mauritania	22.25<br>(0.49 – 182.95)	20.62<br>(0.51 – 157.22)	-7.3<br>(-52.45 – 109.64)
Niger	2459.03<br>(1915.16 – 3117.22)	1423.11<br>(1023.12 – 1962.63)	-42.13<br>(-53.86 – -28.17)
Nigeria	87236.33<br>(66972.33 – 116142.88)	82269.51<br>(64383.37 – 103942.82)	-5.69<br>(-15.05 – 5.89)
São Tomé & Príncipe	0.28<br>(0.16 – 0.49)	0.29<br>(0.15 – 0.48)	2.96<br>(-26.11 – 41.35)
Senegal	2374.07<br>(1756.45 – 3240.86)	1602.89<br>(1242.65 – 2132.73)	-32.48<br>(-44.79 – -19.27)
Sierra Leone	3099.44<br>(2470.92 – 3964.62)	2798.23<br>(2199.78 – 3757.8)	-9.72<br>(-26.22 – 13.81)
Togo	7753.06<br>(5941.32 – 9871.28)	3447.66<br>(2580.1 – 4561.94)	-55.53<br>(-61.93 – -46.9)

Table S2. Number of HIV incident cases and percent change between 2010 and 2019 for GBD super–regions and 204 countries and territories

**Location**	**2010 (95% UI)**	**2019 (95% UI)**	**Percent Change (%)**
**Global**	**2370918.14<br>(2161430.34 – 2702317.7)**	**1989282.21<br>(1760906.83 – 2259348.18)**	**-16.1<br>(-22.31 – -8.07)**
**Central Europe, eastern Europe, and central Asia**	**79538.38<br>(67164.1 – 94473.15)**	**165005.29<br>(133488.11 – 206380.51)**	**107.45<br>(72.66 – 139.13)**
Armenia	85.3<br>(62.81 – 115.16)	127.9<br>(101.33 – 166.94)	49.94<br>(13.26 – 114)
Azerbaijan	159.77<br>(103.12 – 205.25)	304.82<br>(204.41 – 490.6)	90.79<br>(32.97 – 190.98)
Georgia	477.14<br>(327.89 – 697.13)	269.12<br>(174.56 – 460.87)	-43.6<br>(-53.53 – -28.82)
Kazakhstan	694.41<br>(541.63 – 878.53)	2381.56<br>(1571.7 – 3192.79)	242.96<br>(125.18 – 318.08)
Kyrgyzstan	458.45<br>(321.18 – 651.19)	932.01<br>(614.89 – 1375.89)	103.3<br>(31.05 – 184.74)
Mongolia	35.39<br>(13.1 – 74.8)	38.97<br>(10.86 – 80.37)	10.12<br>(-33.87 – 89.93)
Tajikistan	243.91<br>(183.86 – 293.38)	527.35<br>(276.94 – 735.1)	116.21<br>(37.99 – 197.99)

Turkmenistan	158.41<br>(135.87 – 184.05)	270.07<br>(214.51 – 332.48)	70.49<br>(35.44 – 112.93)
Uzbekistan	803.64<br>(120.97 – 1292.08)	2536.9<br>(841.7 – 4197.25)	215.68<br>(26.82 – 1924.41)
Albania	2.34<br>(1.93 – 2.99)	2.69<br>(2.02 – 3.72)	15.18<br>(1.24 – 31.37)
Bosnia and Herzegovina	8.86<br>(5.34 – 11.8)	9.86<br>(5.95 – 14.51)	11.28<br>(-11.83 – 43.4)
Bulgaria	95.13<br>(85.08 – 107.55)	173.82<br>(103.28 – 252.53)	82.71<br>(9.83 – 166)
Croatia	53.43<br>(35.34 – 71.48)	41.87<br>(22.68 – 57.68)	-21.64<br>(-48.3 – 2.72)
Czech Republic	98.04<br>(65.11 – 134.02)	88.59<br>(44.65 – 128.48)	-9.64<br>(-43.32 – 14.86)
Hungary	96.95<br>(75.28 – 115.53)	181.07<br>(109.79 – 243.52)	86.77<br>(43.8 – 135.14)
Montenegro	4.86<br>(3.69 – 5.94)	4.96<br>(3.86 – 6.33)	2.14<br>(-17.66 – 20.61)
North Macedonia	7.29<br>(5.53 – 8.81)	7.25<br>(5.51 – 9.46)	-0.65<br>(-20.26 – 15.9)
Poland	658.55<br>(362.36 – 1167.31)	705.76<br>(319.51 – 1354.31)	7.17<br>(-27.11 – 32.59)
Romania	908.78<br>(723.08 – 1144.04)	648.34<br>(524.55 – 807.08)	-28.66<br>(-40.07 – -13.98)
Serbia	137.88<br>(96.32 – 188.16)	92.78<br>(63 – 144.41)	-32.71<br>(-47.45 – -9.4)
Slovakia	22.75<br>(15.92 – 28.58)	19.55<br>(14.04 – 28.85)	-14.08<br>(-30.62 – 14.57)
Slovenia	16.04<br>(10.27 – 25.72)	14.02<br>(8.77 – 23.69)	-12.58<br>(-28.76 – 14.5)
Belarus	810.56<br>(684.34 – 998.09)	1987.61<br>(1483.16 – 2693.52)	145.21<br>(92.11 – 211.87)
Estonia	224.95<br>(182.58 – 277.13)	168.43<br>(133.02 – 207.85)	-25.12<br>(-45.28 – -2.15)
Latvia	258.49<br>(227.49 – 309.02)	422.19<br>(329.81 – 529.55)	63.33<br>(13.3 – 104.54)
Lithuania	159.38<br>(116.37 – 193.56)	164.84<br>(53.95 – 252.17)	3.42<br>(-62.18 – 54.33)
Moldova	527.98<br>(377.31 – 726.6)	635.14<br>(482.82 – 879.56)	20.3<br>(1.39 – 40.65)
Russia	62384.2<br>(50239 – 76611.85)	118627.76<br>(95416.37 – 150267.07)	90.16<br>(54.5 – 131.46)
Ukraine	9945.51<br>(7145.16 – 11622.72)	33620.08<br>(24871.59 – 44917.29)	238.04<br>(155.71 – 374.99)

**High income**	**87223.34 (56106.69 – 122797.43)**	**118319.39 (69141.69 – 168843.74)**	**35.65 (3.38 – 55.91)**
Australia	1028.79 (624.54 – 1561.34)	1487.28 (870.79 – 2202.79)	44.57 (29.61 – 62.87)
New Zealand	109.18 (59.82 – 164.39)	152.62 (70.7 – 230.21)	39.79 (-10.59 – 95.79)
Brunei	27.34 (16.46 – 41.77)	41.09 (22.93 – 66.35)	50.27 (22.26 – 68.53)
Japan	2582.1 (1584.55 – 3699.98)	2722.69 (1215.13 – 4204.44)	5.44 (-34.89 – 28.1)
South Korea	1442.47 (687.54 – 2735.57)	1257.55 (317.94 – 2409.23)	-12.82 (-76.31 – 47)
Singapore	480.72 (277.2 – 790.33)	547.95 (329.12 – 922.15)	13.98 (-3.62 – 49.95)
Canada	2085.68 (1200.25 – 3209.94)	3323.99 (1576 – 5246.28)	59.37 (8.98 – 103.8)
Greenland	7.22 (4.34 – 11.2)	11.93 (5.32 – 20.36)	65.19 (3.02 – 104.36)
USA	48175.23 (28880.52 – 70971.07)	67134.02 (28606.92 – 104893.11)	39.35 (-24.7 – 68.84)
Argentina	8522.11 (4327.59 – 14724.25)	11633.45 (5956.67 – 19366.34)	36.51 (23.82 – 57.45)
Chile	2550.22 (1486.86 – 4109.72)	4080.78 (2283.5 – 6527.98)	60.02 (47.96 – 73.66)
Uruguay	382.83 (276.21 – 529.91)	859.18 (633.43 – 1113.72)	124.43 (72.74 – 205.18)
Andorra	5.61 (0.26 – 30.15)	7.38 (0.27 – 35.09)	31.57 (-31.78 – 177.14)
Austria	643.76 (347.4 – 1006.73)	671.08 (365.15 – 1011.84)	4.24 (-16.86 – 40.55)
Belgium	1010 (475.93 – 1743.02)	935 (411.48 – 1479.8)	-7.43 (-32.92 – 34.87)
Cyprus	10.64 (7.58 – 15.03)	11.44 (7.76 – 17.54)	7.46 (-5.41 – 22.83)
Denmark	253.19 (157.58 – 363.52)	234.15 (129.1 – 358.8)	-7.52 (-41.11 – 17.31)
Finland	60.53 (36.03 – 89.07)	63.53 (32.09 – 100.99)	4.96 (-43.09 – 49.44)
France	2284.97 (1375.37 – 3490.66)	2570.21 (1514.34 – 3941.88)	12.48 (1.41 – 27.49)
Germany	2096.91 (1466.74 – 3060.16)	2311.84 (1475.53 – 3457.05)	10.25 (-7.96 – 31.76)
Greece	209 (144.37 – 356.85)	145.17 (101.54 – 239.25)	-30.54 (-42.66 – -17.1)

Iceland	4.95<br>(3.55 – 9.41)	8.66<br>(5.46 – 13.67)	75.03<br>(7.56 – 127.38)
Ireland	69.17<br>(38.98 – 114.76)	130.95<br>(60.98 – 207.18)	89.32<br>(28.12 – 179.26)
Israel	600.55<br>(326.16 – 923.23)	650.66<br>(348.56 – 912.2)	8.34<br>(-21.67 – 53.29)
Italy	2976.22<br>(1835.12 – 4405.12)	3850.88<br>(2385.76 – 6145.02)	29.39<br>(-6.38 – 82.65)
Luxembourg	10.27<br>(6 – 16.43)	23.58<br>(12.05 – 37.04)	129.66<br>(74.22 – 208.69)
Malta	19.45<br>(11.68 – 27.61)	26.88<br>(15.24 – 39.97)	38.17<br>(23.9 – 54.36)
Monaco	1.32<br>(0.23 – 4.48)	1.5<br>(0.29 – 6.2)	13.96<br>(-26.7 – 147.3)
Netherlands	851.36<br>(567.63 – 1164.06)	519.24<br>(267.45 – 772.12)	-39.01<br>(-66.76 – -1.23)
Norway	171.22<br>(107.97 – 257.33)	252.25<br>(142.19 – 378.03)	47.32<br>(13.05 – 87.4)
Portugal	931.21<br>(677.3 – 1228.5)	1687.91<br>(1174.97 – 2347.84)	81.26<br>(54.81 – 121.35)
San Marino	1.21<br>(0.23 – 3.77)	1.37<br>(0.27 – 5.17)	13.76<br>(-26.1 – 135.22)
Spain	1856.42<br>(1363.46 – 2828.58)	4663.37<br>(2826.56 – 7021.84)	151.2<br>(61.35 – 263.64)
Sweden	180.18<br>(105.91 – 262.87)	238.54<br>(119.56 – 352.51)	32.39<br>(-1.48 – 58.98)
Switzerland	534.56<br>(277.74 – 784.92)	578.35<br>(235.9 – 871.77)	8.19<br>(-21.31 – 27.32)
UK	5028.11<br>(2841.06 – 7392.78)	5459.11<br>(3137.11 – 7958.94)	8.57<br>(-0.88 – 20.42)
**Latin America and Caribbean**	**120500.41<br>(106877.07 – 133033.62)**	**134658.93<br>(116974.4 – 157243.42)**	**11.75<br>(-1.82 – 23.47)**
Bolivia	1542.83<br>(177.57 – 6697.02)	1597.64<br>(160.69 – 7318.31)	3.55<br>(-34.85 – 58.68)
Ecuador	2609.47<br>(2029.72 – 3458.37)	3556.29<br>(2785.02 – 4653.28)	36.28<br>(18.47 – 53.31)
Peru	6330.02<br>(5026.8 – 7905.15)	6168.13<br>(4401.24 – 9666.44)	-2.56<br>(-25.02 – 33.4)
Antigua and Barbuda	18.95<br>(14.82 – 22.17)	28.02<br>(18.36 – 35.84)	47.87<br>(15.74 – 81.29)
The Bahamas	216.63<br>(197.09 – 243.62)	273.16<br>(236.67 – 332.29)	26.1<br>(13.45 – 45.48)
Barbados	62.62<br>(53.18 – 81.57)	77.69<br>(60.89 – 97.55)	24.07<br>(-2.41 – 54.99)

Belize	134.67<br>(119.86 – 154.28)	210.37<br>(175.56 – 267.42)	56.21<br>(33.9 – 89.02)
Bermuda	12.88<br>(9.57 – 14.93)	15.42<br>(9.82 – 20.8)	19.66<br>(-4.98 – 47.72)
Cuba	1715.18<br>(1092.46 – 2596.07)	2016.85<br>(1102.06 – 3232.09)	17.59<br>(-1.13 – 42.36)
Dominica	16.32<br>(12.57 – 19.17)	19.55<br>(14.35 – 23.94)	19.77<br>(4 – 37.15)
Dominican Republic	4079.67<br>(2869.9 – 5582.13)	2856.47<br>(1027.22 – 6321.88)	-29.98<br>(-67.13 – 28.73)
Grenada	21.3<br>(15.42 – 25.44)	20.21<br>(14.06 – 25.05)	-5.08<br>(-18.54 – 11.98)
Guyana	721.35<br>(484.66 – 942.83)	629.01<br>(451.95 – 780.59)	-12.8<br>(-27.55 – 4.66)
Haiti	13379.18<br>(10045.73 – 17478.72)	8186.01<br>(3893.09 – 14418.22)	-38.82<br>(-64.57 – -2.55)
Jamaica	1014.88<br>(863.72 – 1252.99)	1077.4<br>(836.26 – 1429.41)	6.16<br>(-12.5 – 24.89)
Puerto Rico	465.22<br>(292.09 – 579.97)	716.3<br>(428.93 – 909.01)	53.97<br>(28.92 – 85.58)
Saint Kitts and Nevis	37.37<br>(8.54 – 95.05)	64.34<br>(14.3 – 162.71)	72.15<br>(20.07 – 137.23)
Saint Lucia	24.78<br>(18.76 – 29.96)	25.4<br>(19.56 – 32.29)	2.51<br>(-11.92 – 22.62)
Saint Vincent and the Grenadines	35.33<br>(31.38 – 40.04)	39.08<br>(31.73 – 49.45)	10.6<br>(-8.87 – 35.71)
Suriname	202.43<br>(177.83 – 234.58)	234.23<br>(195.66 – 291.51)	15.71<br>(-0.3 – 34.76)
Trinidad and Tobago	523.12<br>(455.71 – 623.57)	437.07<br>(364.49 – 522)	-16.45<br>(-26.58 – -5.69)
Virgin Islands	16.02<br>(13.24 – 18.79)	19.62<br>(13.42 – 27.43)	22.48<br>(-4.04 – 52.03)
Colombia	5441.86<br>(4616.76 – 6630.4)	9023.16<br>(7068.51 – 11720.46)	65.81<br>(47.7 – 93.36)
Costa Rica	398.05<br>(340.88 – 503.19)	411.29<br>(331.57 – 524.17)	3.33<br>(-7.78 – 22.06)
El Salvador	1921.69<br>(1168.21 – 2565.87)	1112.88<br>(765.16 – 1479.05)	-42.09<br>(-56.33 – -18.92)
Guatemala	1163<br>(700.86 – 2248.99)	1973.36<br>(950.79 – 4068.26)	69.68<br>(-1.08 – 158.5)
Honduras	497.92<br>(402.39 – 617.59)	558.43<br>(398.02 – 750.8)	12.15<br>(-18.85 – 52.71)
Mexico	13063.3<br>(11131.05 – 15424.22)	16893.46<br>(13382.96 – 21306.65)	29.32<br>(19.17 – 42.83)

Nicaragua	1194.46 (923.63 – 1460.41)	1496.71 (1102.05 – 1885.29)	25.3 (1.83 – 51.64)
Panama	1054.83 (863.41 – 1360.61)	1893.76 (1482.09 – 2556.04)	79.53 (62.04 – 98.53)
Venezuela	5506.76 (4638.35 – 7108.14)	6353.95 (4964.49 – 7948.13)	15.38 (-10.78 – 42.01)
Brazil	54817.99 (42490.23 – 65454.91)	64763.23 (50311.14 – 78784.97)	18.14 (2.15 – 29.34)
Paraguay	1459.87 (1008.73 – 1830.3)	1316.26 (936.2 – 1678.21)	-9.84 (-21.98 – 8.96)
**North Africa and Middle East**	**15838.21 (8355.61 – 29911.69)**	**24137.59 (9817.51 – 63607.34)**	**52.4 (-2.87 – 172.14)**
Afghanistan	301.32 (23.94 – 1253.92)	849.66 (38.85 – 3407.13)	181.98 (-1.8 – 558.62)
Algeria	828.43 (90.77 – 3726.32)	675.62 (5.39 – 3202.03)	-18.45 (-98.94 – 123.8)
Bahrain	15.48 (14.15 – 17.5)	15.67 (12.43 – 21.2)	1.22 (-20.43 – 38.03)
Egypt	253.98 (211.94 – 298.64)	506.41 (366.45 – 648.58)	99.39 (44.46 – 161.01)
Iran	1713.13 (1399.7 – 2063.82)	3608.73 (2451.16 – 5064.03)	110.65 (43.85 – 196.91)
Iraq	163.95 (61.21 – 463.63)	261.77 (97.91 – 614.76)	59.67 (10.67 – 273.23)
Jordan	40.36 (32.54 – 54.7)	51.33 (36.21 – 65.56)	27.16 (-12.89 – 71.29)
Kuwait	9.2 (7.66 – 10.93)	13.42 (11.01 – 16.83)	45.8 (29.59 – 75.99)
Lebanon	72.5 (6.1 – 304.12)	121.64 (5.14 – 495.55)	67.77 (-41.35 – 352.32)
Libya	104.81 (6.69 – 536.9)	171.26 (6.77 – 801.62)	63.4 (-28.3 – 406.62)
Morocco	1217.81 (72.5 – 5084.96)	932.98 (35.21 – 4145.76)	-23.39 (-66.19 – 120.57)
Oman	121.57 (6.88 – 221.73)	263.7 (170.17 – 368.76)	116.92 (30.25 – 4005.26)
Palestine	17.25 (13.18 – 24.42)	23.73 (15.53 – 36.71)	37.55 (14.13 – 65.35)
Qatar	4.43 (3.85 – 5.14)	8.27 (6.67 – 10.16)	86.63 (62.67 – 112.05)
Saudi Arabia	729.49 (221.93 – 2146.68)	1036.93 (394.09 – 2468.7)	42.14 (-3.29 – 327.45)
Sudan	8902.35 (3152.32 – 18856.67)	13701.46 (2078.84 – 52440.87)	53.91 (-45.82 – 230.6)

Syria	54.2<br>(42.51 – 76.27)	35.1<br>(22.34 – 53.75)	-35.23<br>(-48.79 – -19.52)
Tunisia	254.02<br>(28.66 – 1076.12)	295.9<br>(40.39 – 1238.5)	16.49<br>(-37.23 – 139.2)
Turkey	389.26<br>(326.75 – 497.61)	444.87<br>(320.38 – 623.35)	14.29<br>(-4.65 – 35.08)
United Arab Emirates	168.14<br>(7.58 – 911.75)	203.3<br>(8.16 – 1008.42)	20.91<br>(-42.95 – 338.38)
Yemen	460.83<br>(31.82 – 1927.1)	891.32<br>(34.11 – 3985.22)	93.42<br>(-31.2 – 354.59)
**South Asia**	**101947.55**<br>**(68983.01 – 148138.43)**	**87909.61**<br>**(50076.57 – 165037.66)**	**-13.77**<br>**(-40.18 – 33.7)**
Bangladesh	754.14<br>(32.86 – 3385.56)	641.76<br>(21.61 – 3526.37)	-14.9<br>(-59.11 – 57.06)
Bhutan	62.89<br>(2.6 – 302.33)	74.85<br>(2.36 – 402.87)	19.01<br>(-41.53 – 116.09)
India	92788.58<br>(63898.64 – 129766.81)	73445.33<br>(44494.79 – 115564.79)	-20.85<br>(-44.18 – 14.11)
Nepal	2650.92<br>(118.67 – 11402.24)	1495.78<br>(50.09 – 8159.18)	-43.57<br>(-73.21 – -1.26)
Pakistan	5691.02<br>(248.5 – 26224.57)	12251.89<br>(1849.88 – 59517.18)	115.28<br>(21.58 – 642.69)
**Southeast Asia, east Asia, and Oceania**	**160841.05**<br>**(139393.68 – 201412.93)**	**168738.92**<br>**(133912.19 – 224341.48)**	**4.91**<br>**(-7.4 – 16.76)**
China	47347.59<br>(35619.48 – 70531.46)	31876.91<br>(15357.06 – 51455.89)	-32.67<br>(-59.2 – -22.48)
North Korea	926.91<br>(15.94 – 5501.18)	973.54<br>(7.95 – 6319.22)	5.03<br>(-72.35 – 110.26)
Taiwan (province of China)	2379<br>(1711.55 – 3425.24)	1517.05<br>(687.56 – 2350.45)	-36.23<br>(-65.86 – -20.94)
American Samoa	1.13<br>(0.43 – 2.47)	1.33<br>(0.54 – 2.7)	17.3<br>(-16.58 – 68.92)
Cook Islands	2.17<br>(0.12 – 9.77)	4.75<br>(0.2 – 19.67)	118.84<br>(5.9 – 338.03)
Fiji	30.14<br>(23.01 – 39.46)	43.29<br>(32.74 – 55.72)	43.63<br>(27.52 – 61.27)
Guam	10.24<br>(4.29 – 21.25)	12.17<br>(5.35 – 24.92)	18.84<br>(-15.03 – 65.7)
Kiribati	3.25<br>(2.45 – 4.03)	4.03<br>(2.67 – 6.41)	23.67<br>(-13.42 – 95.57)
Marshall Islands	5.45<br>(0.08 – 39.8)	9.73<br>(0.14 – 67.4)	78.39<br>(-14.64 – 304.51)

Federated States of Micronesia	55.23<br>(0.71 – 409.37)	92.15<br>(1.31 – 613.96)	66.86<br>(-27.42 – 373.52)
Nauru	1.19<br>(0.07 – 5.61)	2.79<br>(0.12 – 11.88)	134.93<br>(14.37 – 377.93)
Niue	0.17<br>(0.01 – 0.77)	0.42<br>(0.02 – 1.74)	148.62<br>(18.05 – 407.92)
Northern Mariana Islands	1.27<br>(0.47 – 2.76)	1.33<br>(0.54 – 2.57)	4.5<br>(-29.34 – 55.9)
Palau	2.19<br>(0.12 – 10.13)	4.88<br>(0.21 – 20.35)	122.73<br>(5.69 – 359.08)
Papua New Guinea	6212.38<br>(66.15 – 18520.56)	6863.85<br>(30.65 – 23680.32)	10.49<br>(-58.49 – 57.58)
Samoa	17.8<br>(0.25 – 129.61)	35.85<br>(0.55 – 240.9)	101.4<br>(-13.33 – 380.61)
Solomon Islands	52.13<br>(0.73 – 396.97)	102.07<br>(1.58 – 710.3)	95.79<br>(-10.97 – 340.82)
Tokelau	0.14<br>(0.01 – 0.63)	0.35<br>(0.02 – 1.43)	157.59<br>(23.81 – 421.37)
Tonga	3.97<br>(1.33 – 9.46)	4.5<br>(1.67 – 9.95)	13.6<br>(-20.72 – 64.29)
Tuvalu	1.17<br>(0.07 – 5.4)	3.14<br>(0.13 – 12.93)	169.18<br>(24.73 – 446.96)
Vanuatu	31.34<br>(0.38 – 205.6)	48.64<br>(0.7 – 344.36)	55.18<br>(-69.11 – 362.03)
Cambodia	2771.81<br>(861.79 – 5178.63)	898.88<br>(164.52 – 2134.95)	-67.57<br>(-93.22 – -30.81)
Indonesia	11838.99<br>(9966.94 – 15023.82)	14027.39<br>(11519.91 – 18307.39)	18.48<br>(5.36 – 35.26)
Laos	736.91<br>(16.17 – 3844.16)	597.85<br>(10.5 – 3306.39)	-18.87<br>(-66.07 – 55.69)
Malaysia	3532.01<br>(2703.23 – 4158.09)	5155.32<br>(3177.82 – 6449.83)	45.96<br>(11.29 – 74.74)
Maldives	1.03<br>(0.84 – 1.32)	1.28<br>(0.99 – 1.77)	24.82<br>(5.56 – 47.28)
Mauritius	257.42<br>(189.6 – 301.94)	197.31<br>(156.46 – 253.56)	-23.35<br>(-40.83 – 16.32)
Myanmar	15460.1<br>(11127.75 – 21173.48)	8969.79<br>(6623.1 – 12178.79)	-41.98<br>(-47.52 – -34.13)
Philippines	19792.37<br>(12363.26 – 31667.62)	53104.33<br>(29365.65 – 86158.24)	168.31<br>(132.14 – 202.25)
Seychelles	8.06<br>(7.24 – 9.16)	6.07<br>(5.24 – 7.17)	-24.69<br>(-30.16 – -18.88)
Sri Lanka	115.84<br>(90.59 – 142.72)	226.54<br>(142.46 – 331.9)	95.57<br>(13.33 – 184.51)

Thailand	30686.19<br>(24362.3 – 43285.16)	28006.75<br>(21315.68 – 40733.41)	-8.73<br>(-24.16 – 10.69)
Timor-Leste	372.75<br>(5.57 – 2534.9)	342.9<br>(4.89 – 2435.96)	-8.01<br>(-53.56 – 100.48)
Vietnam	17693.58<br>(14896.34 – 21820.73)	15076.98<br>(12113.55 – 19922.54)	-14.79<br>(-25.97 – 2.35)
**Sub-Saharan Africa**	**1805029.2<br>(1585215.7 – 2124300.69)**	**1290512.48<br>(1076399.09 – 1560803.63)**	**-28.5<br>(-35.43 – -19.58)**
Angola	29676.91<br>(20058.87 – 41861.52)	43510.58<br>(22406.3 – 74719.37)	46.61<br>(-8.87 – 114.64)
Central African Republic	11158.71<br>(7701.96 – 15332.31)	6973.66<br>(3010.48 – 14033.11)	-37.5<br>(-66.71 – 3.9)
Congo (Brazzaville)	7182.2<br>(4859.8 – 10129.83)	6620.48<br>(2536.59 – 14671.71)	-7.82<br>(-54.38 – 58.37)
DR Congo	32019.34<br>(22940.11 – 45172.42)	13826.22<br>(7914.24 – 22600.91)	-56.82<br>(-68.84 – -42.35)
Equatorial Guinea	4890.32<br>(3342.7 – 6794.67)	8161.39<br>(3152.83 – 17841.35)	66.89<br>(-16.11 – 209.28)
Gabon	3425.43<br>(2444.63 – 4690.93)	2479.99<br>(977.53 – 5167.37)	-27.6<br>(-62.23 – 25.52)
Burundi	4767.45<br>(3597.2 – 6407.64)	1885.29<br>(1182.62 – 2959.21)	-60.45<br>(-70.29 – -46.79)
Comoros	2.31<br>(0.04 – 13.48)	12.58<br>(0.34 – 64.59)	444.2<br>(112.11 – 2291.7)
Djibouti	1191.04<br>(674.58 – 1980.14)	867.88<br>(317.59 – 1980.53)	-27.13<br>(-58.74 – 11.56)
Eritrea	2063.32<br>(1283.47 – 3157)	1061.08<br>(470.69 – 2083.46)	-48.57<br>(-66.39 – -28.58)
Ethiopia	51456.2<br>(41937.26 – 63980.26)	27977.31<br>(21136.83 – 37149.73)	-45.63<br>(-53.22 – -36.33)
Kenya	103810.93<br>(88518.94 – 121444.67)	67620.99<br>(50604.23 – 88256.79)	-34.86<br>(-45.2 – -22.22)
Madagascar	3661.49<br>(1885.16 – 5979.95)	5616.92<br>(2227.96 – 10719.27)	53.41<br>(-3.6 – 134.61)
Malawi	65043.24<br>(55714.45 – 78452.92)	32295.12<br>(20743.77 – 46924.98)	-50.35<br>(-66.29 – -31.1)
Mozambique	189433.32<br>(164238.53 – 221112.48)	182455.13<br>(100283.86 – 316100.45)	-3.68<br>(-45.62 – 67.26)
Rwanda	10540.61<br>(8360.46 – 13249.19)	5032.6<br>(2942.48 – 8094.28)	-52.26<br>(-67.94 – -31.15)
Somalia	4130.97<br>(2477.67 – 6338.99)	3002.79<br>(1547.94 – 5301.07)	-27.31<br>(-49.27 – 1.25)
South Sudan	10071.83<br>(3844.06 – 19374.54)	8936.73<br>(1553.49 – 26628.9)	-11.27<br>(-63.73 – 72.24)

Uganda	109082.08 (88305.81 – 134634.33)	64692.14 (37837.2 – 102413.33)	-40.69 (-61.28 – -10.9)
Tanzania	104699.94 (82761.06 – 131699.46)	55988.86 (30332.73 – 92084.59)	-46.52 (-67.46 – -17.49)
Zambia	67345.28 (58169.6 – 79417.95)	48808.51 (28262.2 – 77013.85)	-27.52 (-56.25 – 14.88)
Botswana	15011.87 (11255.09 – 19267.31)	9251.09 (4409.51 – 16853.11)	-38.37 (-64.77 – -1.7)
eSwatini	14721.2 (12886.11 – 17164.54)	6899.52 (4079.19 – 11052.85)	-53.13 (-71.35 – -26.02)
Lesotho	25502.85 (22122.79 – 29946.56)	20631.48 (14014.95 – 28536.02)	-19.1 (-45.11 – 13.37)
Namibia	12222.55 (10588.53 – 14451.35)	6348.28 (4417.11 – 8746.14)	-48.06 (-61.8 – -30.31)
South Africa	514837.3 (454321.56 – 598756.38)	366880.48 (286119.75 – 464312.35)	-28.74 (-39.99 – -15.32)
Zimbabwe	79063.56 (67167.8 – 95116.28)	31509.07 (20309.93 – 46651.24)	-60.15 (-72.48 – -43.89)
Benin	5233.89 (3986.5 – 6765.04)	3102.35 (1625.99 – 5437.1)	-40.73 (-62.49 – -11.41)
Burkina Faso	5618.76 (4029.4 – 7582.84)	3003.23 (1929.27 – 4807.54)	-46.55 (-59.24 – -28.75)
Cape Verde	196.37 (89.6 – 393.42)	126.59 (34.52 – 384.73)	-35.54 (-66.03 – 5.41)
Cameroon	48530.79 (37763.62 – 62636.56)	30423.27 (19337.15 – 43691.69)	-37.31 (-56.92 – -14.36)
Chad	10296.18 (7113.62 – 14550.7)	9153.32 (5165.07 – 15504.61)	-11.1 (-42.63 – 31.83)
The Gambia	2213.75 (1346.13 – 3277.69)	2224.59 (1102.85 – 3997.26)	0.49 (-34.7 – 49.07)
Ghana	25473.79 (19205.45 – 33721.47)	17458.82 (10958.66 – 26526.12)	-31.46 (-49.08 – -10.61)
Guinea	10175.14 (7085.19 – 13911.21)	10164.45 (5022.11 – 18279.96)	-0.11 (-36.31 – 50.74)
Guinea–Bissau	2448.28 (1291.85 – 4022.94)	1501.75 (522.22 – 2946.72)	-38.66 (-62.02 – -11.83)
Liberia	3340.2 (2326.79 – 4695.48)	2412.12 (1166.25 – 4590.87)	-27.79 (-52.99 – 7.96)
Mali	8841.64 (6033.17 – 12014.34)	6712.5 (3335.09 – 11834.94)	-24.08 (-50.89 – 12.71)
Mauritania	76.41 (2.29 – 425.23)	66.25 (1.89 – 365.02)	-13.29 (-54.32 – 80.99)
Niger	3076.33 (1997.05 – 4447.46)	1784.62 (778.61 – 3299.15)	-41.99 (-63.71 – -14.84)

Nigeria	148377.1 (120421.8 – 185148.67)	144399.32 (112197.59 – 187486.06)	-2.68 (-16.54 – 18.41)
São Tomé & Príncipe	1.14 (0.36 – 2.33)	1.52 (0.48 – 3.07)	34.17 (-41.22 – 225.66)
Senegal	4045.03 (2977.35 – 5584.41)	2288.18 (1290.11 – 3884.45)	-43.43 (-62.29 – -19.67)
Sierra Leone	6807.22 (5313.47 – 8733.37)	7121.81 (3721.53 – 13171.47)	4.62 (-37.48 – 69.39)
Togo	7921.22 (5581.71 – 10978.39)	4117 (2385.98 – 6897.63)	-48.03 (-63.07 – -28.47)

Table S3: Number of prevalent cases of HIV in 2019 for GBD super–regions and 204 countries and territories

**Location**	**Prevalent cases of HIV (95% UI)**
**Global**	**36848153.96 (35149001.88 – 38856666.01)**
**Central Europe, eastern Europe, and central Asia**	**1539667.32 (1267868.51 – 1893286.31)**
Armenia	1389.42 (1111.43 – 1824.16)
Azerbaijan	3355.87 (2490.91 – 4697.71)
Georgia	4510.62 (3074.2 – 6794.12)
Kazakhstan	16742.87 (13231.76 – 21456.06)
Kyrgyzstan	6191.71 (4364.42 – 9005.16)
Mongolia	418.59 (163.84 – 836.54)
Tajikistan	4528.95 (3441.58 – 5548.36)
Turkmenistan	3755.76 (2978.44 – 4963.33)
Uzbekistan	22344.82 (13096.14 – 31621.82)
Albania	31.98 (20.06 – 50.58)
Bosnia and Herzegovina	132.81 (92.47 – 172.96)
Bulgaria	1775.78 (1575.2 – 1983.07)
Croatia	900.83 (710.11 – 1102.54)

Czech Republic	1671.27<br>(1224.86 – 2210.93)
Hungary	2069 (1654.04 – 2404.22)
Montenegro	87.16<br>(70.6 – 107.73)
North Macedonia	113.95<br>(90.53 – 144.81)
Poland	15674.11<br>(9560.67 – 27520.79)
Romania	14583.45<br>(12823.62 – 16688.09)
Serbia	2302.65<br>(1805.78 – 3192.2)
Slovakia	345.79<br>(261.72 – 456.78)
Slovenia	274.48<br>(155.2 – 458.57)
Belarus	17047.4<br>(12752.63 – 22347.05)
Estonia	3212.75<br>(2781.86 – 3659.83)
Latvia	3794.85<br>(3140.66 – 4566.77)
Lithuania	1879.34<br>(1283.73 – 2380.19)
Moldova	8289.32<br>(5813 – 11576.34)
Russia	1137793.59<br>(938202.14 – 1401106.67)
Ukraine	264448.2<br>(203327.88 – 335088.72)
**High income**	**2869672.97<br>(1681008.14 – 4079542.22)**
Australia	17305.78<br>(11205.1 – 23732.69)
New Zealand	2561.76<br>(1579.06 – 3524.63)
Brunei	542.54<br>(288.61 – 881.87)
Japan	45514.43<br>(24022.55 – 70332.23)
South Korea	29976.86<br>(17559.23 – 47045.64)
Singapore	4650.52<br>(3049.78 – 6865.71)

Canada	92282.82<br>(61219.05 – 127156.15)
Greenland	190.39<br>(97.23 – 306.89)
USA	1743127.93<br>(917751.32 – 2593838.54)
Georgia	4510.62<br>(3074.2 – 6794.12)
Argentina	188657.25<br>(110526.54 – 283951.37)
Chile	55504.71<br>(32762.7 – 85378.2)
Uruguay	11896.96<br>(8723.05 – 15638.47)
Andorra	109.29<br>(6.96 – 453.02)
Austria	17405.05<br>(9761.48 – 25652.72)
Belgium	23813.75<br>(12953.8 – 36400.98)
Cyprus	192.56<br>(113.2 – 302.53)
Denmark	6519.08<br>(4570.91 – 8912.36)
Finland	1361.99<br>(908.37 – 1913.06)
France	102792.77<br>(68949.31 – 141153.58)
Germany	80178.59<br>(58182.93 – 105157.53)
Greece	4407.02<br>(3334.74 – 6731.84)
Iceland	193.4<br>(158.98 – 255.26)
Ireland	2224.39<br>(1389.21 – 3283.12)
Israel	13364.74<br>(7299.85 – 19312)
Italy	96494.94<br>(67739.98 – 131230.26)
Luxembourg	557.89<br>(345.46 – 845.2)
Malta	405.48<br>(247.46 – 580.98)

Monaco	27.61<br>(6.88 – 82.48)
Netherlands	21047.54<br>(14264.25 – 27074.39)
Norway	3454.35<br>(2264.21 – 5034.09)
Portugal	39359.77<br>(32072.88 – 49547.42)
San Marino	23.36<br>(5.39 – 68.68)
Spain	103330.41<br>(87643.79 – 121126.98)
Sweden	5099.08<br>(2682.36 – 7597.2)
Switzerland	22508.06<br>(14420.08 – 30746.17)
UK	131957.31<br>(74519.36 – 189985.03)
**Latin America and Caribbean**	**1880939.77<br>(1636860.98 – 2184447.77)**
Bolivia	16491.88<br>(3194.72 – 57065.2)
Ecuador	39422.75<br>(29255.28 – 54698.16)
Peru	83192.53<br>(65458.4 – 113567.04)
Antigua and Barbuda	279.15<br>(239 – 316.72)
The Bahamas	4114.2<br>(3570.03 – 4951.17)
Barbados	1507.43<br>(1330.42 – 1725.37)
Belize	2657.73<br>(2149.67 – 3328.93)
Bermuda	180.05<br>(157.88 – 198.78)
Cuba	24596.43<br>(15479.89 – 35982.32)
Dominica	212.11<br>(182.96 – 249.09)
Dominican Republic	59038.17<br>(45273.89 – 76699.4)
Grenada	250.03<br>(209.85 – 299.75)

Guyana	9394.47<br>(7997.78 – 11018.03)
Haiti	166904.29<br>(135935.61 – 206367.54)
Jamaica	15823.58<br>(12693.99 – 19650.11)
Puerto Rico	8073.09<br>(6432.74 – 9165.37)
Saint Kitts and Nevis	508.41<br>(153.97 – 1145.1)
Saint Lucia	364.83<br>(313.07 – 453.18)
Saint Vincent and the Grenadines	437.27<br>(371.36 – 534.62)
Suriname	3542.16<br>(3045.9 – 4273.62)
Trinidad and Tobago	9388.78<br>(8351.14 – 10908.44)
Virgin Islands	235.73<br>(208.82 – 264.74)
Colombia	103026.65<br>(81298.88 – 130865.16)
Costa Rica	6763.89<br>(5540.15 – 8458.71)
El Salvador	20679.28<br>(16954.81 – 24551.9)
Guatemala	29371.29<br>(19712.5 – 46967.74)
Honduras	9252.56<br>(7947.3 – 10976.85)
Mexico	219624.5<br>(176922.56 – 272317.39)
Nicaragua	14404.45<br>(11377.18 – 17441.43)
Panama	20996.73<br>(16764.16 – 27477.35)
Venezuela	92235.14<br>(72734.72 – 110791.61)
Brazil	891346.98<br>(765069.1 – 1044866.74)
Paraguay	15841.16<br>(13013.73 – 19375.59)
**North Africa and Middle East**	**203878.67<br>(106281.69 – 407533.7)**

Afghanistan	5125.3<br>(455.35 – 19470.24)
Algeria	9485.27<br>(1533.3 – 33141.14)
Bahrain	243.66<br>(206.95 – 299.83)
Egypt	4438.19<br>(3717.86 – 5258.31)
Iran	27608.06<br>(21290.42 – 34582.99)
Iraq	2449.81<br>(1178.77 – 5161.48)
Jordan	611.7<br>(497.9 – 812.96)
Kuwait	297.04<br>(246.15 – 351.78)
Lebanon	1202.09<br>(136.77 – 4143.43)
Libya	1471.75<br>(224.21 – 7039.1)
Morocco	13334.1<br>(1475.76 – 43095.84)
Oman	2695.04<br>(1946.62 – 3515.21)
Palestine	190.82<br>(108.59 – 339.08)
Qatar	149.98<br>(125.91 – 183.54)
Saudi Arabia	10595.04<br>(4358.22 – 24950.72)
Sudan	106199.62<br>(32755.69 – 292417)
Syria	383.82<br>(276.07 – 589.62)
Tunisia	3031.64<br>(578.94 – 11265.6)
Turkey	5281.18<br>(3881.45 – 7520.94)
United Arab Emirates	2299.28<br>(207.77 – 10041.26)
Yemen	6578.16<br>(706.85 – 25548.67)
**South Asia**	**1937497.17**<br>**(1691087.52 – 2394805.67)**

Bangladesh	8314.63<br>(3680.71 – 29140.41)
Bhutan	724.42<br>(47.87 – 3401.19)
India	1826474.48<br>(1632416.53 – 2051258.53)
Nepal	27605.46<br>(2238.16 – 105538.74)
Pakistan	74378.19<br>(6584.61 – 359119.89)
**Southeast Asia, east Asia, and Oceania**	**2382704.58**<br>**(1856627.63 – 3365035.15)**
China	551426.02<br>(286453.52 – 1027215.4)
North Korea	13630.92<br>(2166.63 – 79674.43)
Taiwan (province of China)	5136.79<br>(2473.09 – 8887.18)
American Samoa	13.96<br>(6.71 – 26.91)
Cook Islands	28.26<br>(1.92 – 110.99)
Fiji	472.09<br>(396.61 – 561.85)
Guam	116.19<br>(52.4 – 237.85)
Kiribati	39.67<br>(28.39 – 54.06)
Marshall Islands	71.57<br>(2.36 – 454.72)
Federated States of Micronesia	898.55<br>(25.42 – 6516.64)
Nauru	15.21<br>(1.05 – 61.94)
Niue	2.48<br>(0.17 – 9.75)
Northern Mariana Islands	14.19<br>(6.73 – 26.99)
Palau	29.56<br>(1.99 – 120.72)
Papua New Guinea	89585.44<br>(2467.16 – 264840.32)
Samoa	253.61<br>(13.96 – 1603.74)

Solomon Islands	722.1<br>(34.95 – 4436.58)
Tokelau	1.99<br>(0.14 – 7.94)
Tonga	41.79<br>(17.86 – 88.55)
Tuvalu	17.68<br>(1.27 – 71.72)
Vanuatu	346.39<br>(24.17 – 2170.66)
Cambodia	70832.73<br>(50386.56 – 90691.28)
Indonesia	290263.09<br>(252122.16 – 339060.58)
Laos	8575.81<br>(3068.55 – 36825.71)
Malaysia	56215.47<br>(47718.02 – 65428.83)
Maldives	16.34<br>(12.44 – 23.58)
Mauritius	3400.37<br>(2903.32 – 4211.69)
Myanmar	215613.5<br>(194444.22 – 238029.34)
Philippines	287664.05<br>(147105.34 – 500581.5)
Seychelles	105.74<br>(86.72 – 137.63)
Sri Lanka	2023.43<br>(1713.4 – 2360.78)
Thailand	527171.9<br>(463153.1 – 613722.19)
Timor-Leste	3632.73<br>(176.57 – 20621.85)
Vietnam	247483.93<br>(202240.19 – 326645.78)
**Sub-Saharan Africa**	**26033793.47**<br>**(25234165.15 – 26850165.67)**
Angola	383909.75<br>(256109.12 – 531017.18)
Central African Republic	114176.74<br>(88127.37 – 150654.53)
Congo (Brazzaville)	96895.21<br>(66388.72 – 143898.43)

DR Congo	379897.3 (307560.62 – 471196.06)
Equatorial Guinea	71378.53 (44945.49 – 109715.8)
Gabon	49558.56 (38133.7 – 65185.69)
Burundi	85495.43 (75575.65 – 98394.06)
Comoros	118.6 (67.86 – 395.47)
Djibouti	13334.83 (7387.76 – 23734.59)
Eritrea	25050.18 (18334.41 – 34114.98)
Ethiopia	791550.25 (714482.82 – 881452.18)
Kenya	1671885.95 (1484084.24 – 1878561.47)
Madagascar	42078.57 (20597.6 – 72319.51)
Malawi	1006346.08 (935370.58 – 1078897.49)
Mozambique	2355345.93 (2031689.97 – 2778668.78)
Rwanda	207718.9 (185780.29 – 229835.97)
Somalia	39195.12 (23368.1 – 63465.69)
South Sudan	112694.11 (41715.2 – 239723.33)
Uganda	1366481.42 (1212028.13 – 1518281.65)
Tanzania	1503310.3 (1366722.03 – 1660045.81)
Zambia	1223914.46 (1129052.95 – 1331778.47)
Botswana	350982.81 (307488.48 – 400883.26)
eSwatini	213547.41 (197725.9 – 231546.31)
Lesotho	374039.93 (351294.84 – 399651.16)
Namibia	205367.31 (192574.27 – 218770.87)

South Africa	7921791.53<br>(7450923.15 – 8394891.07)
Zimbabwe	1227095.76<br>(1148849.07 – 1311033.63)
Benin	70465.1<br>(56692.91 – 84859.74)
Burkina Faso	93881.34<br>(79977.96 – 111594.56)
Cape Verde	2807.05<br>(1557.59 – 4911.21)
Cameroon	587763.36<br>(535496.61 – 642506.58)
Chad	134079.93<br>(103761.69 – 171922.98)
The Gambia	25410.14<br>(16636.78 – 36472.08)
Ghana	306961.88<br>(259942.21 – 364559.89)
Guinea	123874.73<br>(89847.69 – 168216.9)
Guinea–Bissau	31191.26<br>(17877.49 – 47984.86)
Liberia	37782.43<br>(27232.39 – 53080.03)
Mali	110557.99<br>(81758.48 – 146836.68)
Mauritania	1201.03<br>(518.59 – 4948.71)
Niger	38892.96<br>(28225.87 – 53101.26)
Nigeria	1963043.74<br>(1758881.02 – 2206517.82)
São Tomé & Príncipe	27.8<br>(21.97 – 42.43)
Senegal	47356.4<br>(38429.67 – 59331.63)
Sierra Leone	81465.21<br>(60138.45 – 110792.36)
Togo	109713.66<br>(89445.94 – 133932.98)

Table S4. Incidence to prevalence ratio in 2019 for GBD super-regions and 204 countries and territories

Location	Incidence to Prevalence Ratio
**Global**	**0.05 (0.05 to 0.06)**
**Central Europe, eastern Europe, and central Asia**	**0.11 (0.09 to 0.12)**
Armenia	0.09 (0.08 to 0.1)
Azerbaijan	0.09 (0.08 to 0.11)
Georgia	0.06 (0.05 to 0.07)
Kazakhstan	0.14 (0.11 to 0.16)
Kyrgyzstan	0.15 (0.11 to 0.18)
Mongolia	0.09 (0.06 to 0.12)
Tajikistan	0.12 (0.08 to 0.14)
Turkmenistan	0.07 (0.05 to 0.1)
Uzbekistan	0.11 (0.06 to 0.14)
Albania	0.08 (0.07 to 0.11)
Bosnia and Herzegovina	0.07 (0.06 to 0.09)
Bulgaria	0.1 (0.06 to 0.13)
Croatia	0.05 (0.03 to 0.05)
Czech Republic	0.05 (0.03 to 0.06)
Hungary	0.09 (0.07 to 0.11)
Montenegro	0.06 (0.05 to 0.06)
North Macedonia	0.06 (0.06 to 0.07)
Poland	0.05 (0.03 to 0.06)
Romania	0.04 (0.04 to 0.05)
Serbia	0.04 (0.03 to 0.05)
Slovakia	0.06 (0.05 to 0.07)
Slovenia	0.05 (0.04 to 0.07)
Belarus	0.12 (0.1 to 0.13)
Estonia	0.05 (0.04 to 0.06)
Latvia	0.11 (0.08 to 0.14)
Lithuania	0.09 (0.04 to 0.13)
Moldova	0.08 (0.07 to 0.09)
Russia	0.1 (0.09 to 0.12)
Ukraine	0.13 (0.1 to 0.14)
**High income**	**0.04 (0.03 to 0.05)**
Australia	0.09 (0.07 to 0.1)
New Zealand	0.06 (0.04 to 0.07)
Brunei	0.08 (0.06 to 0.09)
Japan	0.06 (0.04 to 0.07)
South Korea	0.04 (0.01 to 0.06)
Singapore	0.12 (0.09 to 0.14)
Canada	0.04 (0.02 to 0.05)
Greenland	0.06 (0.04 to 0.08)

USA	0.04 (0.02 to 0.05)
Georgia	0.06 (0.05 to 0.07)
Argentina	0.06 (0.05 to 0.07)
Chile	0.07 (0.07 to 0.08)
Uruguay	0.07 (0.06 to 0.08)
Andorra	0.07 (0.02 to 0.12)
Austria	0.04 (0.03 to 0.05)
Belgium	0.04 (0.02 to 0.05)
Cyprus	0.06 (0.05 to 0.07)
Denmark	0.04 (0.02 to 0.04)
Finland	0.05 (0.03 to 0.06)
France	0.03 (0.02 to 0.03)
Germany	0.03 (0.02 to 0.04)
Greece	0.03 (0.03 to 0.04)
Iceland	0.04 (0.03 to 0.06)
Ireland	0.06 (0.04 to 0.07)
Israel	0.05 (0.04 to 0.06)
Italy	0.04 (0.03 to 0.05)
Luxembourg	0.04 (0.03 to 0.05)
Malta	0.07 (0.06 to 0.07)
Monaco	0.05 (0.03 to 0.09)
Netherlands	0.02 (0.02 to 0.03)
Norway	0.07 (0.06 to 0.08)
Portugal	0.04 (0.03 to 0.05)
San Marino	0.06 (0.03 to 0.1)
Spain	0.05 (0.03 to 0.06)
Sweden	0.05 (0.03 to 0.06)
Switzerland	0.03 (0.01 to 0.03)
UK	0.04 (0.04 to 0.04)
**Latin America and Caribbean**	**0.07 (0.06 to 0.08)**
Bolivia	0.1 (0.05 to 0.13)
Ecuador	0.09 (0.08 to 0.1)
Peru	0.07 (0.06 to 0.09)
Antigua and Barbuda	0.1 (0.07 to 0.12)
The Bahamas	0.07 (0.06 to 0.07)
Barbados	0.05 (0.04 to 0.06)
Belize	0.08 (0.07 to 0.09)
Bermuda	0.09 (0.06 to 0.11)
Cuba	0.08 (0.07 to 0.1)
Dominica	0.09 (0.07 to 0.11)
Dominican Republic	0.05 (0.02 to 0.08)
Grenada	0.08 (0.07 to 0.09)
Guyana	0.07 (0.05 to 0.09)
Haiti	0.05 (0.03 to 0.07)

Jamaica	0.07 (0.06 to 0.08)
Puerto Rico	0.09 (0.07 to 0.1)
Saint Kitts and Nevis	0.13 (0.08 to 0.16)
Saint Lucia	0.07 (0.06 to 0.08)
Saint Vincent and the Grenadines	0.09 (0.07 to 0.12)
Suriname	0.07 (0.06 to 0.08)
Trinidad and Tobago	0.05 (0.04 to 0.05)
Virgin Islands	0.08 (0.06 to 0.11)
Colombia	0.09 (0.08 to 0.1)
Costa Rica	0.06 (0.06 to 0.07)
El Salvador	0.05 (0.04 to 0.06)
Guatemala	0.07 (0.04 to 0.09)
Honduras	0.06 (0.05 to 0.07)
Mexico	0.08 (0.07 to 0.08)
Nicaragua	0.1 (0.09 to 0.12)
Panama	0.09 (0.08 to 0.1)
Venezuela	0.07 (0.06 to 0.08)
Brazil	0.07 (0.06 to 0.08)
Paraguay	0.08 (0.07 to 0.1)
**North Africa and Middle East**	**0.12 (0.08 to 0.17)**
Afghanistan	0.17 (0.07 to 0.24)
Algeria	0.07 (0 to 0.12)
Bahrain	0.06 (0.05 to 0.09)
Egypt	0.11 (0.09 to 0.14)
Iran	0.13 (0.1 to 0.16)
Iraq	0.11 (0.08 to 0.14)
Jordan	0.08 (0.06 to 0.11)
Kuwait	0.05 (0.04 to 0.05)
Lebanon	0.1 (0.03 to 0.17)
Libya	0.12 (0.03 to 0.18)
Morocco	0.07 (0.02 to 0.13)
Oman	0.1 (0.08 to 0.11)
Palestine	0.12 (0.1 to 0.15)
Qatar	0.06 (0.05 to 0.07)
Saudi Arabia	0.1 (0.07 to 0.15)
Sudan	0.13 (0.06 to 0.19)
Syria	0.09 (0.08 to 0.1)
Tunisia	0.1 (0.06 to 0.13)
Turkey	0.08 (0.08 to 0.09)
United Arab Emirates	0.09 (0.03 to 0.14)
Yemen	0.14 (0.04 to 0.2)
**South Asia**	**0.05 (0.03 to 0.07)**
Bangladesh	0.08 (0.01 to 0.13)
Bhutan	0.1 (0.04 to 0.14)

India	0.04 (0.03 to 0.06)
Nepal	0.05 (0.02 to 0.09)
Pakistan	0.16 (0.12 to 0.29)
**Southeast Asia, east Asia, and Oceania**	**0.07 (0.06 to 0.08)**
China	0.06 (0.03 to 0.09)
North Korea	0.07 (0 to 0.13)
Taiwan (province of China)	0.3 (0.17 to 0.45)
American Samoa	0.1 (0.08 to 0.11)
Cook Islands	0.17 (0.09 to 0.22)
Fiji	0.09 (0.08 to 0.11)
Guam	0.1 (0.09 to 0.12)
Kiribati	0.1 (0.08 to 0.13)
Marshall Islands	0.14 (0.04 to 0.18)
Federated States of Micronesia	0.1 (0.04 to 0.17)
Nauru	0.18 (0.1 to 0.24)
Niue	0.17 (0.09 to 0.23)
Northern Mariana Islands	0.09 (0.08 to 0.11)
Palau	0.17 (0.09 to 0.22)
Papua New Guinea	0.08 (0.01 to 0.11)
Samoa	0.14 (0.04 to 0.19)
Solomon Islands	0.14 (0.04 to 0.18)
Tokelau	0.18 (0.09 to 0.23)
Tonga	0.11 (0.09 to 0.13)
Tuvalu	0.18 (0.08 to 0.23)
Vanuatu	0.14 (0.03 to 0.18)
Cambodia	0.01 (0 to 0.02)
Indonesia	0.05 (0.04 to 0.06)
Laos	0.07 (0 to 0.12)
Malaysia	0.09 (0.06 to 0.11)
Maldives	0.08 (0.07 to 0.09)
Mauritius	0.06 (0.05 to 0.07)
Myanmar	0.04 (0.03 to 0.05)
Philippines	0.18 (0.17 to 0.2)
Seychelles	0.06 (0.05 to 0.07)
Sri Lanka	0.11 (0.08 to 0.15)
Thailand	0.05 (0.04 to 0.07)
Timor-Leste	0.09 (0.02 to 0.14)
Vietnam	0.06 (0.05 to 0.08)
**Sub-Saharan Africa**	**0.05 (0.04 to 0.06)**
Angola	0.11 (0.08 to 0.15)
Central African Republic	0.06 (0.03 to 0.1)
Congo (Brazzaville)	0.07 (0.04 to 0.1)
DR Congo	0.04 (0.02 to 0.05)
Equatorial Guinea	0.11 (0.07 to 0.17)

Gabon	0.05 (0.03 to 0.08)
Burundi	0.02 (0.02 to 0.03)
Comoros	0.11 (0 to 0.19)
Djibouti	0.07 (0.04 to 0.1)
Eritrea	0.04 (0.02 to 0.06)
Ethiopia	0.04 (0.03 to 0.04)
Kenya	0.04 (0.03 to 0.05)
Madagascar	0.13 (0.09 to 0.18)
Malawi	0.03 (0.02 to 0.04)
Mozambique	0.08 (0.05 to 0.11)
Rwanda	0.02 (0.02 to 0.04)
Somalia	0.08 (0.05 to 0.1)
South Sudan	0.08 (0.03 to 0.12)
Uganda	0.05 (0.03 to 0.07)
Tanzania	0.04 (0.02 to 0.06)
Zambia	0.04 (0.02 to 0.06)
Botswana	0.03 (0.01 to 0.04)
eSwatini	0.03 (0.02 to 0.05)
Lesotho	0.06 (0.04 to 0.07)
Namibia	0.03 (0.02 to 0.04)
South Africa	0.05 (0.04 to 0.06)
Zimbabwe	0.03 (0.02 to 0.04)
Benin	0.04 (0.03 to 0.07)
Burkina Faso	0.03 (0.02 to 0.05)
Cape Verde	0.05 (0.02 to 0.08)
Cameroon	0.05 (0.03 to 0.07)
Chad	0.07 (0.05 to 0.1)
The Gambia	0.09 (0.06 to 0.12)
Ghana	0.06 (0.04 to 0.08)
Guinea	0.08 (0.05 to 0.11)
Guinea-Bissau	0.05 (0.03 to 0.07)
Liberia	0.06 (0.04 to 0.09)
Mali	0.06 (0.04 to 0.08)
Mauritania	0.06 (0 to 0.1)
Niger	0.05 (0.03 to 0.07)
Nigeria	0.07 (0.06 to 0.09)
São Tomé & Príncipe	0.05 (0.02 to 0.09)
Senegal	0.05 (0.03 to 0.07)
Sierra Leone	0.09 (0.06 to 0.13)
Togo	0.04 (0.02 to 0.05)

Table S5. Incidence to mortality ratio in 2019 for GBD super-regions and 204 countries and territories

Location	Incidence to Mortality Ratio
**Global**	**1.94 (1.76 to 2.12)**
**Central Europe, eastern Europe, and central Asia**	**4.76 (3.96 to 5.78)**
Armenia	5.08 (4.16 to 6.52)
Azerbaijan	6.27 (4.27 to 11.02)
Georgia	4.06 (2.96 to 6.34)
Kazakhstan	7.23 (4.99 to 9.51)
Kyrgyzstan	4.68 (3.15 to 6.69)
Mongolia	1.78 (1.25 to 3.2)
Tajikistan	6.77 (4.14 to 8.55)
Turkmenistan	2.18 (1.71 to 2.69)
Uzbekistan	3.69 (1.34 to 5.75)
Albania	1.5 (1.26 to 1.8)
Bosnia and Herzegovina	3.31 (2.36 to 5.38)
Bulgaria	2.92 (1.79 to 3.99)
Croatia	2.6 (1.55 to 3.72)
Czech Republic	2.77 (1.56 to 4.1)
Hungary	3 (2.02 to 3.93)
Montenegro	2.44 (1.98 to 3.15)
North Macedonia	2.84 (2.24 to 3.8)
Poland	3.2 (1.49 to 5.5)
Romania	2.93 (2.38 to 3.69)
Serbia	1.45 (1 to 1.92)
Slovakia	3.19 (2.38 to 4.59)
Slovenia	3.7 (2.43 to 5.55)
Belarus	5.44 (4.11 to 7.22)
Estonia	3.87 (3.03 to 4.9)
Latvia	3.15 (2.48 to 3.9)
Lithuania	2.31 (0.78 to 3.48)
Moldova	3.08 (2.4 to 4.09)
Russia	4.74 (3.94 to 5.75)
Ukraine	3.99 (3.03 to 5.22)
**High income**	**3.91 (2.65 to 4.98)**
Australia	8.62 (5.7 to 11.83)
New Zealand	8.1 (4.4 to 11.43)
Brunei	5.84 (3.83 to 8.03)
Japan	4.97 (2.82 to 6.35)
South Korea	3.54 (1.02 to 5.56)
Singapore	8.96 (5.66 to 13.87)
Canada	4.42 (2.27 to 6.64)

Greenland	4.01 (2.18 to 5.72)
USA	3.24 (1.47 to 4.57)
Georgia	4.06 (2.96 to 6.34)
Argentina	4.62 (2.54 to 7.19)
Chile	5.89 (3.5 to 8.72)
Uruguay	3.63 (2.65 to 4.7)
Andorra	2.14 (0.48 to 5.25)
Austria	5.21 (3.36 to 7.98)
Belgium	3.86 (2 to 6.05)
Cyprus	2.14 (1.57 to 2.98)
Denmark	3.42 (2.02 to 4.96)
Finland	4.58 (2.37 to 6.55)
France	2.38 (1.57 to 3.43)
Germany	2.26 (1.49 to 3.33)
Greece	3.58 (2.59 to 5.12)
Iceland	6.13 (3.97 to 9.24)
Ireland	9.01 (4.46 to 13.34)
Israel	6.65 (4.1 to 10.09)
Italy	4.01 (2.61 to 5.92)
Luxembourg	4.72 (2.67 to 6.71)
Malta	10.33 (6.6 to 13.99)
Monaco	1.44 (0.76 to 3.21)
Netherlands	2.68 (1.5 to 3.99)
Norway	10.74 (6.48 to 14.68)
Portugal	2.68 (1.9 to 3.73)
San Marino	1.78 (0.98 to 3.95)
Spain	4.43 (2.72 to 6.6)
Sweden	5.9 (3.29 to 7.88)
Switzerland	3.97 (1.9 to 5.52)
UK	6.41 (4.87 to 8.16)
**Latin America and Caribbean**	**2.61 (2.31 to 3.04)**
Bolivia	1.99 (1.32 to 5.42)
Ecuador	2.64 (2.05 to 3.41)
Peru	2.31 (1.92 to 2.92)
Antigua and Barbuda	3.3 (2.26 to 4.1)
The Bahamas	1.93 (1.68 to 2.34)
Barbados	2.23 (1.77 to 2.79)
Belize	2.29 (1.91 to 2.86)
Bermuda	2.2 (1.43 to 2.9)
Cuba	4.1 (2.53 to 5.77)
Dominica	3.13 (2.46 to 3.69)
Dominican Republic	1.68 (0.77 to 2.93)
Grenada	2.89 (2.17 to 3.61)
Guyana	2.3 (1.81 to 2.78)

Haiti	1.15 (0.66 to 1.77)
Jamaica	2.06 (1.58 to 2.72)
Puerto Rico	2.66 (1.65 to 3.33)
Saint Kitts and Nevis	1.81 (1.34 to 2.76)
Saint Lucia	2.82 (2.24 to 3.68)
Saint Vincent and the Grenadines	1.75 (1.44 to 2.14)
Suriname	1.94 (1.62 to 2.42)
Trinidad and Tobago	1.7 (1.42 to 2.05)
Virgin Islands	1.95 (1.38 to 2.59)
Colombia	3.14 (2.49 to 4.09)
Costa Rica	2.33 (1.87 to 3)
El Salvador	1.32 (1.07 to 1.8)
Guatemala	2.37 (1.18 to 4.56)
Honduras	3.43 (2.54 to 5.42)
Mexico	2.8 (2.19 to 3.55)
Nicaragua	2.42 (1.9 to 3.51)
Panama	3.15 (2.44 to 4.23)
Venezuela	2.65 (2.12 to 3.29)
Brazil	3.12 (2.54 to 3.72)
Paraguay	2.05 (1.71 to 2.71)
**North Africa and Middle East**	**2.37 (1.48 to 4.19)**
Afghanistan	2.44 (1.64 to 7.37)
Algeria	2.24 (0.07 to 6.88)
Bahrain	1.97 (1.29 to 3.12)
Egypt	5.58 (4.3 to 6.98)
Iran	2.88 (2.46 to 3.27)
Iraq	2.93 (1.74 to 5.49)
Jordan	1.73 (1.43 to 2.13)
Kuwait	3.7 (3 to 4.69)
Lebanon	1.78 (0.92 to 4.02)
Libya	1.9 (1.17 to 5.5)
Morocco	1.66 (0.69 to 4.85)
Oman	2.46 (1.95 to 3.79)
Palestine	1.72 (1.3 to 2.27)
Qatar	1.78 (1.61 to 2.12)
Saudi Arabia	1.43 (1.11 to 2.43)
Sudan	2.48 (0.71 to 5.18)
Syria	2.24 (1.73 to 2.77)
Tunisia	1.87 (1.1 to 5.8)
Turkey	1.8 (1.49 to 2.26)
United Arab Emirates	1.13 (0.62 to 4.24)
Yemen	2.33 (1.33 to 7.35)
**South Asia**	**1.4 (0.9 to 1.94)**
Bangladesh	1.67 (0.95 to 5.95)

Bhutan	2.24 (1.24 to 6.08)
India	1.29 (0.81 to 1.81)
Nepal	0.93 (0.36 to 3.03)
Pakistan	2.85 (1.77 to 28.35)
**Southeast Asia, east Asia, and Oceania**	**1.83 (1.6 to 2.19)**
China	0.87 (0.53 to 1.13)
North Korea	1.1 (0.23 to 2.17)
Taiwan (province of China)	7.74 (3.63 to 11.64)
American Samoa	2.33 (1.6 to 4.31)
Cook Islands	2.51 (1.41 to 5.47)
Fiji	3.55 (2.9 to 4.33)
Guam	1.57 (1.14 to 2.06)
Kiribati	2.09 (1.66 to 2.84)
Marshall Islands	2.13 (1.22 to 6.04)
Federated States of Micronesia	1.63 (0.52 to 2.95)
Nauru	2.85 (1.69 to 5.43)
Niue	2.49 (1.4 to 5.4)
Northern Mariana Islands	2 (1.37 to 4.06)
Palau	2.29 (1.26 to 4.62)
Papua New Guinea	1.37 (0.02 to 1.97)
Samoa	2.36 (1.33 to 7.21)
Solomon Islands	2.18 (1.26 to 6.05)
Tokelau	2.59 (1.45 to 5.47)
Tonga	2.11 (1.38 to 3.95)
Tuvalu	2.83 (1.61 to 5.98)
Vanuatu	2.22 (1.11 to 6.17)
Cambodia	0.45 (0.09 to 0.84)
Indonesia	2.31 (2.03 to 2.69)
Laos	1.39 (0.71 to 5.06)
Malaysia	2.98 (2.2 to 3.62)
Maldives	1.43 (1.15 to 1.84)
Mauritius	1.8 (1.41 to 2.31)
Myanmar	1.47 (1.1 to 1.84)
Philippines	8 (4.8 to 11.85)
Seychelles	1.3 (1.12 to 1.58)
Sri Lanka	4.86 (3.22 to 6.08)
Thailand	1.41 (1.25 to 1.67)
Timor-Leste	1.32 (0.5 to 3.26)
Vietnam	2.12 (1.73 to 2.49)
**Sub-Saharan Africa**	**1.63 (1.44 to 1.82)**
Angola	2.34 (1.5 to 3.35)
Central African Republic	1.02 (0.51 to 1.67)
Congo (Brazzaville)	1.35 (0.51 to 2.86)
DR Congo	1.09 (0.69 to 1.49)

Equatorial Guinea	3.48 (1.82 to 5.66)
Gabon	1.79 (0.9 to 3.12)
Burundi	0.68 (0.44 to 1)
Comoros	5.15 (2.84 to 15.19)
Djibouti	0.9 (0.43 to 1.56)
Eritrea	0.67 (0.38 to 1.04)
Ethiopia	0.92 (0.77 to 1.09)
Kenya	1.12 (0.91 to 1.33)
Madagascar	2.04 (1.19 to 3.06)
Malawi	1.65 (1.12 to 2.24)
Mozambique	2.27 (1.43 to 3.44)
Rwanda	1.15 (0.71 to 1.68)
Somalia	0.85 (0.56 to 1.21)
South Sudan	1.83 (0.67 to 3.35)
Uganda	2.31 (1.53 to 3.29)
Tanzania	1.61 (0.96 to 2.36)
Zambia	1.65 (1.03 to 2.47)
Botswana	1.2 (0.63 to 1.9)
eSwatini	1.35 (0.83 to 2.06)
Lesotho	1.37 (0.98 to 1.84)
Namibia	1.28 (0.92 to 1.71)
South Africa	2 (1.67 to 2.35)
Zimbabwe	1.02 (0.67 to 1.44)
Benin	1.26 (0.82 to 1.84)
Burkina Faso	0.81 (0.55 to 1.13)
Cape Verde	1.91 (0.84 to 3.1)
Cameroon	1.15 (0.79 to 1.54)
Chad	1.62 (1.07 to 2.32)
The Gambia	1.83 (1.18 to 2.62)
Ghana	1.07 (0.77 to 1.4)
Guinea	2.09 (1.35 to 3.02)
Guinea-Bissau	1.23 (0.69 to 1.77)
Liberia	1.31 (0.8 to 1.99)
Mali	1.29 (0.82 to 1.87)
Mauritania	2.35 (0.73 to 7.41)
Niger	1.06 (0.6 to 1.59)
Nigeria	1.56 (1.35 to 1.95)
São Tomé & Príncipe	2.9 (1.22 to 4.97)
Senegal	1.22 (0.81 to 1.69)
Sierra Leone	2.27 (1.4 to 3.43)
Togo	0.99 (0.65 to 1.37)

Authors' Contributions

Managing the estimation or publications process

Deepa Jahagirdar, Amanda Novotney, Molly Biehl, Stephen S Lim, Simon Hay, Christopher J L Murray, and Hmwe H Kyu.

Writing the first draft of the manuscript

Deepa Jahagirdar, Magdalene Walters, Jeffrey W Eaton, Joshua Salomon, Amanda Novotney, and Hmwe H Kyu.

Primary responsibility for applying analytical methods to produce estimates

Deepa Jahagirdar, Magdalene Walters, Tahvi D Frank, and Austin Carter.

Primary responsibility for seeking, cataloguing, extracting, or cleaning data; designing or coding figures and tables

Deepa Jahagirdar, Edmond Brewer, Magdalene Walters.

Providing data or critical feedback on data sources

Edmond D Brewer, Tahvi D Frank, Hedayat Abbastabar, E Abhilash, Laith Abu-Raddad, Victor Adekanmbi, Qorinah Adnani, Saira Afzal, Soodabeh Aghababaei, Bright Ahinkorah, Sajjad Ahmad, Sepideh Ahmadi, Muktar Ahmed, Tarik Ahmed Rashid, Yusra Ahmed Salih, Addis Aklilu, Chisom Akunna, Hanadi Al Hamad, Fares Alahdab, Fahad Alanezi, Kefyalew Alene, Liaqat Ali, Sami Almustanyir, Nelson Alvis-Guzman, Hubert Amu, Catalina Liliana Andrei, Davood Anvari, Jalal Arabloo, Olatunde Aremu, Desta Atnafu, Beatriz Paulina Ayala Quintanilla, Muluken Ayza, Samad Azari, Maciej Banach, Fabio Barra, Amadou Barrow, Sanjay Basu, Habtamu Gebrehana Belay, Tezera Berheto, Woldesellassie Bezabhe, Till Bärnighausen, Sadia Bibi, Obasanjo Bolarinwa, Archith Boloor, Azizbek Boltaev, Danilo Buonsenso, Souranshu Chatterjee, Soosanna Kumary Chattu, Vijay Kumar Chattu, Dinh-Toi Chu, Rosa Couto, Xiaochen Dai, Lalit Dandona, Rakhi Dandona, José das Neves, Louisa Degenhardt, Feleke Demeke, Govinda Dhungana, Mostafa Dianatinasab, Linh Phuong Doan, Andem Effiong, Maysaa El Sayed Zaki, Rajesh Elayedath, Aman Endries, Sharareh Eskandarieh, Simone Ferrero, Lorenzo Ferro Desideri, Takeshi Fukumoto, Mohamed Gad, Tushar Garg, Hailay Gesesew, Keyghobad Ghadiri, Ahmad Ghashghaee, Syed Amir Gilani, Themba Ginindza, Mahaveer Golechha, Harish Gugnani, Bhawna Gupta, Veer Bala Gupta, Vivek Gupta, Senad Handanagic, Shafiul Haque, Soheil Hassanipour, Kamal Hezam, Praveen Hoogar, Mehdi Hosseinzadeh, Segun Ibitoye, Bulat Idrisov, Kevin Ikuta, Seyed Sina Irvani, Nahlah Elkudssiah Ismail, Vardhmaan Jain, Zubair Kabir, Rohollah Kalhor, Patrick Katoto, Gbenga Kayode, Himanshu Khajuria, Nauman Khalid, Ejaz Khan, Khaled Khatab, Min Seo Kim, Yun Jin Kim, Adnan Kisa, Sezer Kisa, Sindhura Lakshmi Koulmane Laxminarayana, Kewal Krishan, Barthelemy Kuate Defo, G Anil Kumar, Manasi Kumar, Dharmesh Lal, Savita Lasrado, Kate LeGrand, Xuefeng Liu, Hassan Magdy Abd El Razek, Alaa Makki, Wondimu Manamo, Mohammad Ali Mansournia, Francisco Martins-Melo, Amir Masoud Rahmani, Ziad Memish, Ritesh Menezes, Endalkachew Mengesha, Nour Mheidly, Babak Moazen, Mokhtar Mohammadi, Salahuddin Mohammed, Shafiu Mohammed, Ali H Mokdad, Mariam Molokhia, Mohammad Ali Moni, Ghobad Moradi, Sumaira Mubarik, Jean Nachega, Ahamarshan Nagarajan, Aparna Narayana, Georges Nguefack-Tsague, Trang Nguyen, Chukwudi Nnaji, Jean Jacques Noubiap, Thomas Nyirenda, Onome Oghenetega, Andrew Olagunju, Mahesh P A,Jagadish Rao Padubidri, Fatemeh Pashazadeh Kan, Shrikant Pawar, Veincent Christian Pepito, Marina Pinheiro, Khem Pokhrel, Richard Pollok, Maarten Postma, Zahiruddin Quazi Syed, Raghu Radhakrishnan, Vafa Rahimi-Movaghar, Chhabi

Ranabhat, Chythra Rao, Sowmya J Rao, Priya Rathi, David L Rawaf, Salman Rawaf, Lemma Regassa, Andre Renzaho, Nima Rezaei, Rezaul Ripon, Voilet Rodrigues, Godfrey Rwegerera, Umar Saeed, Rajesh Sagar, Marwa Salem, Mehrnoosh Samaei, Abdallah Samy, Milena Santric-Milicevic, Satish Saroshe, Brijesh Sathian, Maheswar Satpathy, Monika Sawhney, Allen Seylani, Masood Shaikh, Mohammed Shaka, Morteza Shamsizadeh, Mohammed Shannawaz, Adithi Shetty, Jae Il Shin, K Shivakumar, Jasvinder Singh, Valentin Skryabin, Anna Skryabina, Sergey Soshnikov, Emma Spurlock, Animut Tamiru, Elvis Tarkang, Nuno Taveira, Gizachew Tessema, Giang Thu Vu, Marcos Tovani-Palone, Bach Tran, Alexander Tsai, Tudorel Andrei, Saif Ullah, Bhaskaran Unnikrishnan, Sahel Valadan Tahbaz, Jorge Villafañe, Sergey Vladimirov, Bay Vo, Avina Vongpradith, Yasir Waheed, Richard Wamai, Ronny Westerman, Seyed Hossein Yahyazadeh Jabbari, Siyan Yi, Birhanu Yirdaw, Naohiro Yonemoto, Chuanhua Yu, Ismaeel Yunusa, Mikhail Zastrozhin, Zhi-Jiang Zhang, Alimuddin Zumla, Jeffrey W Eaton, Mohsen Naghavi, Haidong Wang, Stephen S Lim, and Hmwe H Kyu.

Developing methods or computational machinery

Deepa Jahagirdar, Magdalene Walters, Tahvi D Frank, Austin Carter, Saira Afzal, Soodabeh Aghababaei, Muktar Ahmed, Tarik Ahmed Rashid, Yusra Ahmed Salih, Davood Anvari, Habtamu Gebrehana Belay, Xiaochen Dai, Mostafa Dianatinasab, Linh Phuong Doan, Mehdi Hosseinzadeh, Ramaiah Itumalla, Adnan Kisa, Sezer Kisa, Alaa Makki, Amir Masoud Rahmani, Endalkachew Mengesha, Mokhtar Mohammadi, Salahuddin Mohammed, Ali H Mokdad, Zahiruddin Quazi Syed, Fakher Rahim, Chhabi Ranabhat, Abdallah Samy, Maheswar Satpathy, Animut Tamiru, Bay Vo, Ronny Westerman, Jeffrey W Eaton, Mohsen Naghavi, and Haidong Wang.

Providing critical feedback on methods or results

Deepa Jahagirdar, Tahvi D Frank, Austin Carter, Hedayat Abbastabar, E Abhilash, Eman Abu-Gharbieh, Laith Abu-Raddad, Victor Adekanmbi, Daniel Adeyinka, Qorinah Adnani, Saira Afzal, Soodabeh Aghababaei, Bright Ahinkorah, Sajjad Ahmad, Keivan Ahmadi, Ehsan Ahmadpour, Muktar Ahmed, Tarik Ahmed Rashid, Yusra Ahmed Salih, Addis Aklilu, Tayyaba Akram, Chisom Akunna, Hanadi Al Hamad, Fares Alahdab, Fahad Alanezi, Ekaterina Aleksandrova, Kefyalew Alene, Liaqat Ali, Vahid Alipour, Sami Almustanyir, Nelson Alvis-Guzman, Edward Ameyaw, Hubert Amu, Catalina Liliana Andrei, Davood Anvari, Jalal Arabloo, Olatunde Aremu, Judie Arulappan, Beatriz Paulina Ayala Quintanilla, Muluken Ayza, Samad Azari, Darshan B, Maciej Banach, Fabio Barra, Amadou Barrow, Sanjay Basu, Shahrzad Bazargan-Hejazi, Habtamu Gebrehana Belay, Tezera Berheto, Woldesellassie Bezabhe, Yihienew Bezabih, Akshaya Bhagavathula, Nikha Bhardwaj, Pankaj Bhardwaj, Krittika Bhattacharyya, Till Bärnighausen, Sadia Bibi, Ali Bijani, Archith Boloor, Danilo Buonsenso, Katrin Burkart, Zahid Butt, Chao Cao, Jaykaran Charan, Souranshu Chatterjee, Soosanna Kumary Chattu, Vijay Kumar Chattu, Sonali Choudhari, Dinh-Toi Chu, Richard Cowden, Berihun Dachew, Omid Dadras, Amare Dagnew, Saad Dahlawi, Xiaochen Dai, Lalit Dandona, Rakhi Dandona, José das Neves, Louisa Degenhardt, Feleke Demeke, Abebaw Desta, Keshab Deuba, Deepak Dhamnetiya, Govinda Dhungana, Mostafa Dianatinasab, Daniel Diaz, Shirin Djalalinia, Fariba Dorostkar, Hisham Edinur, Andem Effiong, Sahar Eftekharzadeh, Maysaa El Sayed Zaki, Rajesh Elayedath, Muhammed Elhadi, Ziad El-Khatib, Aisha Elsharkawy, Aklilu Endalamaw, Aman Endries, Sharareh Eskandarieh, Ifeanyi Ezeonwumelu, Mohammad Farahmand, Emerito Jose Faraon, Abidemi Fasanmi, Simone Ferrero, Lorenzo Ferro Desideri, Irina Filip, Florian Fischer, Morenike Folayan, Masoud Foroutan, Takeshi Fukumoto, Mohamed Gad, Muktar Gadanya, Abhay Gaidhane, Tushar Garg, Reta Gayesa, Eyob Alemayehu Gebreyohannes, Hailay Gesesew, Ahmad Ghashghaee, Mahaveer Golechha, Harish Gugnani, Bhawna Gupta, Sapna Gupta, Veer Bala Gupta, Vivek Gupta, Samer Hamidi, Senad Handanagic, Shafiul Haque, Harapan Harapan, Arief Hargono, Ahmed Hasaballah, Abdiwahab Hashi, Shoaib Hassan, Soheil Hassanipour, Khezar Hayat, Ileana Heredia-Pi, Ramesh Holla, Praveen Hoogar, Mohammad Hoque, Mostafa Hosseini, Mehdi Hosseinzadeh, Mohamed

Hsairi, Rabia Hussain, Segun Ibitoye, Bulat Idrisov, Kevin Ikuta, Olayinka Ilesanmi, Irena Ilic, Milena Ilic, Seyed Sina Irvani, M Mofizul Islam, Nahlah Elkudssiah Ismail, Ramaiah Itumalla, Ihoghosa Iyamu, Roxana Jabbarinejad, Vardhmaan Jain, Ravi Jha, Nitin Joseph, Ali Kabir, Zubair Kabir, Rohollah Kalhor, Feroze Kaliyadan, Ashwin Kamath, Tanuj Kanchan, Himal Kandel, Getinet Kassahun, Patrick Katoto, Gbenga Kayode, Ermiyas Kebede, Hafte Kebede, Himanshu Khajuria, Nauman Khalid, Ejaz Khan, Gulfaraz Khan, Khaled Khatab, Min Seo Kim, Yun Jin Kim, Adnan Kisa, Sezer Kisa, Sonali Kochhar, Parvaiz Koul, Sindhura Lakshmi Koulmane Laxminarayana, Ai Koyanagi, Kewal Krishan, Barthelemy Kuate Defo, G Anil Kumar, Manasi Kumar, Nithin Kumar, Dharmesh Lal, Savita Lasrado, Zohra Lassi, Jane Lee, Yeong Yeh Lee, Kate LeGrand, Xuefeng Liu, Hassan Magdy Abd El Razek, Phetole Mahasha, Azeem Majeed, Alaa Makki, Ahmad Malik, Wondimu Manamo, Mohammad Ali Mansournia, Francisco Martins-Melo, Amir Masoud Rahmani, Seyedeh Zahra Masoumi, Ritesh Menezes, Endalkachew Mengesha, Hayimro Merie, Amanual Mersha, Tomislav Mestrovic, Peter Meylakhs, Nour Mheidly, Ted Miller, Andreea Mirica, Babak Moazen, Yousef Mohammad, Mokhtar Mohammadi, Arif Mohammed, Salahuddin Mohammed, Shafiu Mohammed, Modhurima Moitra, Ali H Mokdad, Mariam Molokhia, Mohammad Ali Moni, Ghobad Moradi, Yousef Moradi, Christine Mpundu-Kaambwa, Sumaira Mubarik, Sandra Munro, Lillian Mwanri, Jean Nachega, Ahamarshan Nagarajan, Aparna Narayana, Muhammad Naveed, Biswa Nayak, Sabina Nduaguba, Sandhya Neupane Kandel, Georges Nguefack-Tsague, Trang Nguyen, Chukwudi Nnaji, Jean Jacques Noubiap, Onome Oghenetega, Andrew Olagunju, Oluwatomi Owopetu, Mahesh P A, Dr. Jagadish Rao. Padubidri, Smita Pakhale, Tarang Parekh, Shrikant Pawar, Veincent Christian Pepito, Emmanuel Peprah, Richard Pollok, Maarten Postma, Zahiruddin Quazi Syed, Amir Radfar, Raghu Radhakrishnan, Fakher Rahim, Vafa Rahimi-Movaghar, Shadi Rahimzadeh, Mosiur Rahman, Pradhum Ram, Chhabi Ranabhat, Priyanga Ranasinghe, Chythra Rao, Sowmya J Rao, Priya Rathi, David L Rawaf, Salman Rawaf, Lemma Regassa, Inayat ur Rehman, Andre Renzaho, Nima Rezaei, Omid Rezahosseini, Mohammad sadegh Rezai, Aziz Rezapour, Rezaul Ripon, Voilet Rodrigues, Denis Roshchin, Godfrey Rwegerera, Umar Saeed, Sahar Saeedi Moghaddam, Rajesh Sagar, KM Saif-Ur-Rahman, Mehrnoosh Samaei, Abdallah Samy, Milena Santric-Milicevic, Brijesh Sathian, Maheswar Satpathy, Monika Sawhney, Aletta Schutte, Allen Seylani, Masood Shaikh, Mohammed Shaka, Hina Shamshad, Mohammed Shannawaz, Jae Il Shin, K Shivakumar, Jasvinder Singh, Valentin Skryabin, Anna Skryabina, Ranjani Somayaji, Mu'awiyyah Sufiyan, Hooman Tadbiri, Birkneh Tadesse, Eyayou Tadesse, Animut Tamiru, Elvis Tarkang, Nuno Taveira, Yohannes Tekalegn, Gizachew Tessema, Rekha Thapar, Giang Thu Vu, Marcos Tovani-Palone, Eugenio Traini, Bach Tran, Alexander Tsai, Tudorel Andrei, Biruk tusa, Saif Ullah, Chukwuma Umeokonkwo, Bhaskaran Unnikrishnan, Sahel Valadan Tahbaz, Jorge Villafañe, Bay Vo, Yasir Waheed, Richard Wamai, Guan Wang, Yanzhong Wang, Paul Ward, Ronny Westerman, Andrea Winkler, Seyed Hossein Yahyazadeh Jabbari, Taklo Yazie, Siyan Yi, Vahit Yiğit, Birhanu Yirdaw, Naohiro Yonemoto, Chuanhua Yu, Ismaeel Yunusa, Mikhail Zastrozhin, Zhi-Jiang Zhang, Alimuddin Zumla, Jeffrey W Eaton, Joshua Salomon, Mohsen Naghavi, Laura Dwyer-Lindgren, Haidong Wang, Stephen S Lim, Simon Hay, Christopher J L Murray, and Hmwe H Kyu.

Drafting the work or revising it critically for important intellectual content

Deepa Jahagirdar, Magdalene Walters, Amanda Novotney, Eman Abu-Gharbieh, Victor Adekanmbi, Daniel Adeyinka, Saira Afzal, Soodabeh Aghababaei, Bright Ahinkorah, Sepideh Ahmadi, Muktar Ahmed, Fares Alahdab, Kefyalew Alene, Liaqat Ali, Sami Almustanyir, Nelson Alvis-Guzman, Hubert Amu, Catalina Liliana Andrei, Jalal Arabloo, Olatunde Aremu, Beatriz Paulina Ayala Quintanilla, Muluken Ayza, Darshan B, Maciej Banach, Amadou Barrow, Sanjay Basu, Habtamu Gebrehana Belay, Tezera Berheto, Woldesellassie Bezabhe, Yihienew Bezabih, Akshaya Bhagavathula, Krittika Bhattacharyya, Till Bärnighausen, Sadia Bibi, Catherine Bisignano, Obasanjo Bolarinwa, Azizbek Boltaev, Nikolay Briko, Danilo Buonsenso, Katrin Burkart, Chao Cao, Souranshu Chatterjee, Soosanna Kumary Chattu, Vijay Kumar Chattu, Dinh-Toi Chu, Richard Cowden, José das Neves, Louisa Degenhardt, Feleke Demeke,

Deepak Dhamnetiya, Govinda Dhungana, Mostafa Dianatinasab, Daniel Diaz, Linh Phuong Doan, Andem Effiong, Sahar Eftekharzadeh, Maysaa El Sayed Zaki, Muhammed Elhadi, Shaimaa El-Jaafary, Ziad El-Khatib, Aisha Elsharkawy, Aklilu Endalamaw, Aman Endries, Sharareh Eskandarieh, Sayeh Ezzikouri, Emerito Jose Faraon, Irina Filip, Florian Fischer, Morenike Folayan, Masoud Foroutan, Takeshi Fukumoto, Mohamed Gad, Muktar Gadanya, Tushar Garg, Reta Gayesa, Eyob Alemayehu Gebreyohannes, Hailay Gesesew, Abera Getachew Obsa, Ahmad Ghashghaee, Ionela-Roxana Glăvan, Ekterina Glushkova, Bhawna Gupta, Sapna Gupta, Veer Bala Gupta, Vivek Gupta, Harapan Harapan, Ahmed Hasaballah, Abdiwahab Hashi, Ileana Heredia-Pi, Kamal Hezam, Ramesh Holla, Mohammad Hoque, Mostafa Hosseini, Segun Ibitoye, Bulat Idrisov, Olayinka Ilesanmi, Irena Ilic, Milena Ilic, Seyed Sina Irvani, Nahlah Elkudssiah Ismail, Ramaiah Itumalla, Ihoghosa Iyamu, Vardhmaan Jain, Ranil Jayawardena, Ravi Jha, Nitin Joseph, Ali Kabir, Feroze Kaliyadan, Himal Kandel, Getinet Kassahun, Patrick Katoto, Gbenga Kayode, Ermiyas Kebede, Himanshu Khajuria, Ejaz Khan, Gulfaraz Khan, Khaled Khatab, Yun Jin Kim, Adnan Kisa, Sezer Kisa, Sonali Kochhar, Vladimir Korshunov, Parvaiz Koul, Sindhura Lakshmi Koulmane Laxminarayana, Ai Koyanagi, Kewal Krishan, Barthelemy Kuate Defo, Manasi Kumar, Alexander Kwarteng, Iván Landires, Savita Lasrado, Jeffrey Lazarus, Yeong Yeh Lee, Kate LeGrand, Hassan Magdy Abd El Razek, Phetole Mahasha, Azeem Majeed, Ahmad Malik, Wondimu Manamo, Francisco Martins-Melo, Ziad Memish, Ritesh Menezes, Endalkachew Mengesha, Hayimro Merie, Tomislav Mestrovic, Nour Mheidly, Ted Miller, Babak Moazen, Yousef Mohammad, Arif Mohammed, Shafiu Mohammed, Ali H Mokdad, Mariam Molokhia, Mohammad Ali Moni, Ghobad Moradi, Sandra Munro, Lillian Mwanri, Jean Nachega, Ahamarshan Nagarajan, Biswa Nayak, Sandhya Neupane Kandel, Georges Nguefack-Tsague, Trang Nguyen, Molly Nixon, Jean Jacques Noubiap, Virginia Nuñez-Samudio, Onome Oghenetega, Andrew Olagunju, Babayemi Olakunde, Mahesh P A,Jagadish Rao Padubidri, Smita Pakhale, Tarang Parekh, Shrikant Pawar, Veincent Christian Pepito, Khem Pokhrel, Roman Polibin, Richard Pollok, Maarten Postma, Zahiruddin Quazi Syed, Amir Radfar, Fakher Rahim, Vafa Rahimi-Movaghar, Pradhum Ram, Chhabi Ranabhat, Priyanga Ranasinghe, Chythra Rao, Sowmya J Rao, David L Rawaf, Salman Rawaf, Lemma Regassa, Inayat ur Rehman, Andre Renzaho, Nima Rezaei, Rezaul Ripon, Voilet Rodrigues, Godfrey Rwegerera, Umar Saeed, Rajesh Sagar, KM Saif-Ur-Rahman, Marwa Salem, Mehrnoosh Samaei, Abdallah Samy, Milena Santric-Milicevic, Maheswar Satpathy, Aletta Schutte, Allen Seylani, Mohammed Shaka, Mohammed Shannawaz, K Shivakumar, Jasvinder Singh, Valentin Skryabin, Anna Skryabina, Ranjani Somayaji, Sergey Soshnikov, Dan Stein, Mu'awiyyah Sufiyan, Hooman Tadbiri, Eyayou Tadesse, Animut Tamiru, Elvis Tarkang, Nuno Taveira, Fisaha Tesfay, Giang Thu Vu, Marcos Tovani-Palone, Bach Tran, Alexander Tsai, Tudorel Andrei, Saif Ullah, Bhaskaran Unnikrishnan, Sahel Valadan Tahbaz, Jorge Villafañe, Yasir Waheed, Richard Wamai, Yanzhong Wang, Paul Ward, Ronny Westerman, Andrea Winkler, Lalit Yadav, Seyed Hossein Yahyazadeh Jabbari, Vahit Yiğit, Naohiro Yonemoto, Mikhail Zastrozhin, Anasthasia Zastrozhina, Joshua Salomon, Jeffrey W Eaton, Mohsen Naghavi, Laura Dwyer-Lindgren, Simon Hay, Christopher J L Murray, and Hmwe H Kyu.

Extracting, cleaning, or cataloging data; designing or coding figures and tables

Deepa Jahagirdar, Magdalene Walters, Edmond D Brewer, Soodabeh Aghababaei, Muktar Ahmed, Liaqat Ali, Hubert Amu, Obasanjo Bolarinwa, Takeshi Fukumoto, Tushar Garg, Ahmed Hasaballah, Christine Lin, Emilie Maddison, Modhurima Moitra, Ali H Mokdad, Shrikant Pawar, Zahiruddin Quazi Syed, Chhabi Ranabhat, Abdallah Samy, Maheswar Satpathy, Emma Spurlock, Avina Vongpradith, Vahit Yiğit, Mikhail Zastrozhin, Anasthasia Zastrozhina, , and Mohsen Naghavi.

Managing the overall research enterprise

Deepa Jahagirdar, Amanda Novotney, Molly Biehl, Lalit Dandona, Ali H Mokdad, Molly Nixon, Mohsen Naghavi, Simon Hay, Christopher J L Murray, and Hmwe H Kyu.
